# Evolution and Epidemic Spread of SARS-CoV-2 in Colombia: A Year into the Pandemic

**DOI:** 10.3390/vaccines9080837

**Published:** 2021-07-30

**Authors:** Sergio Castañeda, Luz H. Patiño, Marina Muñoz, Nathalia Ballesteros, Enzo Guerrero-Araya, Daniel Paredes-Sabja, Carolina Flórez, Sergio Gomez, Carolina Ramírez-Santana, Gustavo Salguero, Juan E. Gallo, Alberto E. Paniz-Mondolfi, Juan David Ramírez

**Affiliations:** 1 Centro de Investigaciones en Microbiología y Biotecnología-UR (CIMBIUR), Facultad de Ciencias Naturales, Universidad del Rosario, Bogotá 111221, Colombia; sergio.castaneda@urosario.edu.co (S.C.); luzh.patino@urosario.edu.co (L.H.P.); claudia.munoz@urosario.edu.co (M.M.); nathalia.ballesteros@urosario.edu.co (N.B.); 2 ANID—Millennium Science Initiative Program—Millennium Nucleus in the Biology of the Intestinal Microbiota, Santiago 7510689, Chile; e.guerreroaraya@uandresbello.edu (E.G.-A.); dparedes-sabja@bio.tamu.edu (D.P.-S.); 3 Microbiota-Host Interactions and Clostridia Research Group, Facultad de Ciencias de la Vida, Universidad Andrés Bello, Santiago 7510689, Chile; 4 Department of Biology, Texas A&M University, College Station, TX 77843, USA; 5 Instituto Nacional de Salud, Bogotá 111321, Colombia; aflorez@ins.gov.co (C.F.); sgomez@ins.gov.co (S.G.); 6 Centro de Estudio de Enfermedades Autoinmunes (CREA), Escuela de Medicina y Ciencias de la Salud, Universidad del Rosario, Bogotá 111221, Colombia; heily.ramirez@urosario.edu.co; 7 Instituto Distrital de Ciencia, Biotecnología e Innovación en Salud (IDCBIS), Bogotá 111611, Colombia; gsalguero@idcbis.org.co; 8 Genoma Ces Biotechnologies, Universidad CES, Medellin 050021, Colombia; jegallo@ces.edu.co; 9 Icahn School of Medicine at Mount Sinai, New York, NY 10029, USA; Alberto.Paniz-mondolfi@mountsinai.org

**Keywords:** SARS-CoV-2, epidemic models, genomic surveillance, Colombia

## Abstract

Current efforts to understand the epidemiology, transmission dynamics and emergence of novel SARS-CoV-2 variants worldwide has enabled the scientific community to generate critical information aimed at implementing disease surveillance and control measures, as well as to reduce the social, economic and health impact of the pandemic. Herein, we applied an epidemic model coupled with genomic analysis to assess the SARS-CoV-2 transmission dynamics in Colombia. This epidemic model allowed to identify the geographical distribution, *Rt* dynamics and predict the course of the pandemic considering current implementation of countermeasures. The analysis of the incidence rate per 100,000 inhabitants carried out across different regions of Colombia allowed visualizing the changes in the geographic distribution of cases. The cumulative incidence during the timeframe March 2020 to March 2021 revealed that Bogotá (8063.0), Quindío (5482.71), Amazonas (5055.68), Antioquia (4922.35) and Tolima (4724.41) were the departments with the highest incidence rate. The highest median *Rt* during the first period evaluated was 2.13 and 1.09 in the second period; with this model, we identified improving opportunities in health decision making related to controlling the pandemic, diagnostic testing capacity, case registration and reporting, among others. Genomic analysis revealed 52 circulating SARS-CoV-2 lineages in Colombia detected from 774 genomes sequenced throughout the first year of the pandemic. The genomes grouped into four main clusters and exhibited 19 polymorphisms. Our results provide essential information on the spread of the pandemic countrywide despite implementation of early containment measures. In addition, we aim to provide deeper phylogenetic insights to better understand the evolution of SARS-CoV-2 in light of the latent emergence of novel variants and how these may potentially influence transmissibility and infectivity.

## 1. Introduction

Coronavirus disease 2019 (COVID-19), caused by Severe Acute Respiratory Syndrome Coronavirus 2 (SARS-CoV-2), represents a global challenge, with over 185 million infections and close to 4 million deaths reported worldwide [1]. Although COVID-19 is now considered a global disease, the virus has spread in a heterogeneous fashion across different countries, with certain regions throughout the globe being particularly affected. Such is the case for South America, with over 33,885,519 cases and 1,033,800 deaths reported as of 11 July 2021 [1]. Poverty, a lack of critical-care resources, a crippled public healthcare infrastructure, and distrust in public governance have made South America as well as other developing countries disproportionally susceptible to the pandemic [2,3]. In addition, multiple emerging variants now circulating globally have raised concerns about increased transmissibility, infectivity and ability to evade vaccine immunity [4]. Amongst these is the Gamma variant (P.1 lineage), originating and linked to multiple outbreaks across Manaus, the capital of the state of Amazonas, and now spreading globally as one of the main lineages of epidemiological concern worldwide [4]. 

The aggressive transmission dynamics witnessed across highly endemic regions has prompted interest to investigate emerging trends in SARS-CoV-2 genetic diversity [5,6] and implement genomic surveillance studies that could provide the basis to build and apply epidemic models, estimate viral spread (*R_t_* value), and facilitate the rapid identification of positive cases to help mitigate virus dispersion as exercised in other countries [6,7,8,9,10,11,12], including several in South America [2,13]. *R_t_* represents the effective reproduction rate of the virus calculated for each region in a specific time. In general, when *R_t_* is greater than 1, there is a high potential for epidemic spread in the population, and the higher the *R_t_*, the more aggressive the public health interventions are required to control the epidemic [14]. 

**Figure 1:**
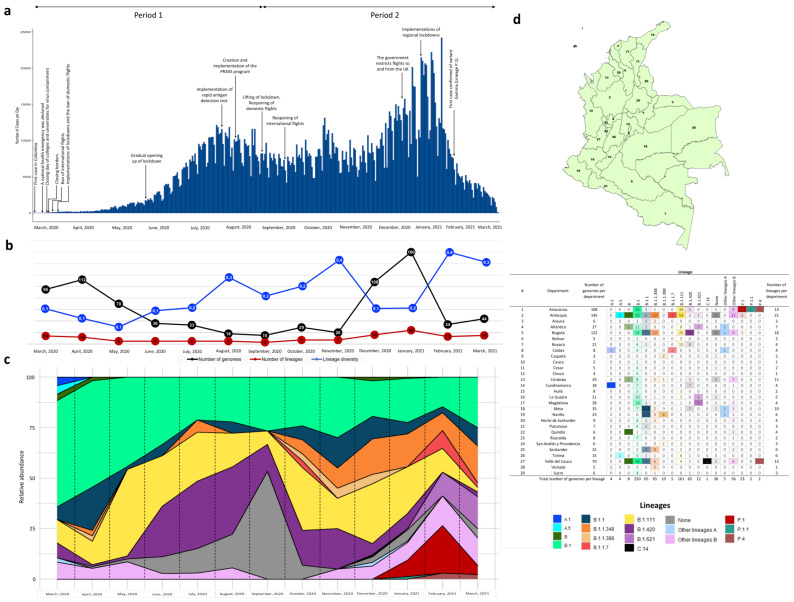
Time series of cases and genomic surveillance in Colombia during the first year of the COVID-19 pandemic (since 6 March 2020 to 6 March 2021). (**a**) Number of cases reported through time. This graph refers to 2,273,245 cases based on the notification date. Two time periods are shown in relation to the dynamics of the cases and the behavior of the control measures taken by the national government. The first period was from 6 March to 10 August 2020. The second period was from 11 August 2020 to 6 March 2021. The main measures and strategies implemented by the national and local governments are also identified. (**b**) Genomic sampling through time at the national level. Black line corresponds to the total number of genomes sequenced per month, the red line to the total number of lineages identified monthly and the blue line is an index of the estimated lineage diversity calculated in this work using the number of lineages detected with respect to the number of genomes sequenced during the same monthly window; the numbers are included in the circles of the line graphs. (**c**) Relative abundance of the lineages circulating in Colombia through the months. (**d**) Geographical distribution of the lineages identified in Colombia distributed by department. The number included inside of the table indicate the number of genomes identified for each lineage.

To date, Colombia is the country in South America with the second highest number of COVID-19 cases [15,16]. It is important to highlight that during the first year of the pandemic in the country, control strategies have been implemented based mainly on restricting mobility, social distancing and closing schools and airports, among other non-pharmaceutical interventions, which have shown effectiveness in controlling events of this nature in other countries [17,18,19,20]. Only on 17 February 2021, the vaccination process started in Colombia. The timing of the application of these strategies and the moment that these restrictions were lifted, in relation to the behavior of the pandemic in Colombia, have shown many shortcomings and negative results (Figure 1a). However, it is important to highlight that the population’s compliance with the measures has been variable and often insufficient. The socioeconomic difficulties inherent to the country’s situation and the inadequate education and understanding of the public health context by the community, which results in an inappropriate perception of risk, are factors that have a negative impact on the management and effectiveness of the measures taken, which has facilitated the dispersion of COVID-19 [19,20,21,22]. Several epidemic modelling studies based on *R_t_* estimations have been performed in an attempt to decipher the current infection dynamics, with the caveat that most of these studies have been purely descriptive and restricted to certain geographical regions at the beginning of the pandemic [3,23,24,25,26,27]. This has precluded an accurate assessment on the impact of control strategies across wide settings throughout the pandemic period. On the other hand, genomic surveillance of SARS-CoV-2 in Colombia has been restricted to a limited number of genomes sequenced to date (less than 1% of the total number of confirmed cases) [15,28,29,30].

In this study, we used a publicly available dataset [31] to characterize the epidemiological landscape of SARS-CoV-2 and apply an epidemic model coupled with genetic data to evaluate the genomic diversity, evolution and epidemiological behavior at a regional and countrywide level throughout the first year of the pandemic. This combined genetic and epidemiological approach will allow us to understand the transmission dynamics since the first introduction and how the pandemic has evolved before and after the different public health interventions. In addition, the analysis framework implemented here, based on a novel epidemic model and genomic surveillance, may provide an outline that could be applied to other South American nations, where there have been major challenges in response to the ongoing pandemic.

## 2. Materials and Methods

### 2.1. Epidemiological Data

We analyzed case counts from the *Instituto Nacional de Salud* database, which can be accessed at: https://www.datos.gov.co/Salud-y-Protecci-n-Social/Casos-positivos-de-COVID-19-en-Colombia/gt2j-8ykr/data (accessed on 7 March 2021) [32]. This database is available for public consultation and contains the variables that we used in our research, such as the notification date, notification region, age, sex and deaths. Notification date corresponds to the date on which a suspect case was identified and reported to the Public Health System, and posteriorly, confirmed as positive. Additionally, we use a public database that reports the number of processed tests of COVID-19 in Colombia by region. This database was downloaded from https://www.datos.gov.co/Salud-y-Protecci-n-Social/Pruebas-PCR-procesadas-de-COVID-19-en-Colombia-Dep/8835-5baf (accessed on 7 March 2021) [33]. It is important to note that the data source from which the information was taken makes daily adjustments to the number of cases and the confirmation of deaths by COVID-19, so these data may vary slightly depending on the date of consultation, which is why the absolute values described here correspond to the cut-off date on which the information was downloaded, in this case, as of 6 March 2021. The population data were obtained from *Departamento Administrativo Nacional de Estadística* (DANE). We downloaded data related to the population projections for 2020, available at https://www.dane.gov.co/index.php/estadisticas-por-tema/demografia-y-poblacion/estimaciones-del-cambio-demografico (accessed on 7 March 2021) [34]. We used these data to calculate the incidence and mortality rates per 100,000 people, reported as such in the present research. 

### 2.2. Geographical Incidence Distribution 

As we described previously, we calculated the incidence rate per 100,000 people from positive cases of COVID-19 reported by the Instituto Nacional de Salud [35] and from the population projection for 2020 obtained from DANE [34]. We calculated these values and created maps to show the bimestrial dynamics of the incidence rate: March–April, May–June, July–August, September–October, November–December and January–February (2021). Maps were made using ArcGIS 10 software. 

### 2.3. Effective Reproduction Number

R0 (“R-nothing”) describes the reproduction factor of a disease; that is, to how many more people an infected person transmits the disease. However, in this value, the countermeasures that are being implemented to slow the spread of the virus are not considered. Therefore, the most critical measure to follow is *Rt*, which considers the effective reproduction factor over time. Thus, *Rt* represents the effective virus reproduction rate calculated for a particular region over a given time. *Rt* represents the effective reproduction rate of the virus calculated for each region over a specific time. In general terms, when *Rt* is greater than 1, the infection can spread in the population, and the higher *Rt*, the more and better measures are necessary to control the epidemic. They allow us to estimate how many secondary infections are likely to occur from a single infection in a specific area and is useful to track the effectiveness of the control measures and other factors affecting the spread of the epidemic [14].

We have implemented a model Rt.live, a widely followed online resource that tracks COVID-19 spread and provides real-time estimates of *Rt*. Details on the methodology used to calculate *Rt* are publicly available online in https://github.com/rtcovidlive/covid-model (accessed on 24 February 2021) [36]. This model has been used mainly in the United States to calculate and follow the *Rt* dynamics in different regions, and likewise, to evaluate control measures [18,37]. According to what is described in the GitHub repository of the Rt.Live model, first, this model uses a simple generative logic to explain how an initial pool of infected people spreads the disease at each timepoint, according to the current reproduction factor. Here we are showing the model proposed to identify, on day *t*, the number of newly infected people:

(1) $$y_{t}=y_{t\;-\;1}\cdot \;R_{e\;}(t)$$

However, this assumption in the generative process above is that an infected person is only infectious for a single day and that it then takes just one day to infect other people [36]. Nevertheless, the time it takes the main person to infect others follows a distribution. This person can infect another one the next day, two days later, or the next, etc. This delay distribution is officially known as “generation time” and this model presents it as a probability distribution [36,38]. To include this effect in the generative model, Rt.live, instead of determining the new cases of day t depending only on the new cases of day *t* − 1, the model considers the potentially new cases in all the previous days because it could have been 5 days between when one person got infected and infected another person. Therefore, it is necessary to take into account all these previously infected people and their probability of infecting other people today. The Rt.live model accomplishes this by weighting the number of newly infected people *i* days ago—$y_{t\;-\;i}$—by the generation time $g_{i}$ for that particular delay, as well as the effective reproduction number on that day $R_{e}$ (t − *i*):

(2) $$y_{t}=\sum_{i=1}^{M}y_{t\;-\;i}R_{e\;}(t-i)g_{i}$$

In accordance with the above, the generative model allows estimating how people transmit the disease from one person to another. However, in the data collected through public health, we do not have data on when people transmitted the disease, merely data on who tested positive. Therefore, the model must further delay this function when an infected patient appears as a positive test in the evaluated data. To make this adjustment, the model implements a distribution of the delay between infection and the confirmed positive test, also known as the delay distribution [39]. Given that the previously defined considerations are considered, the model performs a convolution of the functions of how many people were infected each day with the distribution of the onset delay, in order to estimate how many people will appear with a positive test in one day specific. Then, the noise is scaled and added based on known test volumes through a negative binomial with an exposure parameter for a given day to retrieve an observed series [36]. 

Additionally, the model also considers that the number of positive tests performed depends on the number of people who were tested, since as more tests are performed, more positive cases will be identified. The foregoing is fundamental within modeling since there is great variability in the number of tests that are carried out over time, which is related to the operational capacity in the different regions and in the country, as well as to the fact that fewer tests are generally performed on weekends, thus skewing the estimate. Therefore, in the model the test exposure, $e_{t}$, which corresponds to a normalized quantity proportional to the number of tests performed, is multiplied by the number of positive tests of the generative process. Thus, the expected number of positive tests, ${\tilde{z}}_{t}$, will be

(3) $${\tilde{z}}_{t}=z_{t}\cdot e_{t}$$

where ${\tilde{z}}_{t}$ is the output of the generative model with the delays applied [36].

The model implemented here handles quite a few issues that most methods to calculate $R_{e}$ (t) models do not. Due to its generative design and by sampling it with PyMC3, the model makes inferences about the underlying infection (incidence) rates that are not distorted by changes in test exposure. By accounting for the number of performed tests, the model can make a “fair” comparison not only between regions, but also between countries [36]. In summary, the *generation time* and testing the *delay distribution* are at the core of the model. It is important to consider that the Rt.live model assumes that the *probability of getting tested* is independent of being infected or not, and only depends on the total number of tests performed. That means that this model assumes the following: (A) strategies to focus testing on exposed contact persons do not work very well. We use this model because we consider that particularly with high incidences this is a reasonable assumption. (B) Testing is done by more or less randomly sampling from the population [36]. 

The use of the delay distribution for the calculation of *Rt* in the Rt.live model is the main reason that there is a displacement of the peaks of *Rt* with respect to the methodology used by the WHO model, which estimate the rate of transmission of COVID-19 using the number of reported cases on specific dates according to the R package “EpiEstim” [40,41]. Therefore, in addition to executing the model based on *Rt* live, we decided to simultaneously execute the model proposed by the WHO and thus obtain the value of *Rt* by both methods.

### 2.4. Phylogenomic Inference and Nucleotide Diversity

A total of 1849 publicly available SARS-CoV-2 genomes, generated worldwide, were downloaded from GISAID (Global Initiative on Sharing All Influenza Data) [42] for comparative phylogenetic analysis. This dataset was selected based in three criteria; first, considering a temporal window previously established to Colombian genomes analyzed here (6 March 2020 until 6 March 2021); second; selecting only those genomes with a maximum percentage of Ns of 20%; and finally, for each category (South America and Other regions) the first member per pangolin lineage was included. The complete data set was assembled, aligned and manually curated to remove the 5′- and 3′-untranslated regions, and later the SNPs were extracted using SNP-sites v.2.5.1 [43] and used to build a maximum likelihood (ML) tree using IQtree2 v.1.6.1, following the parameters previously described [15]. Monophyletic groups of genomes constituted mainly (>90.0%) by genomes of Colombian origin were defined clusters and were subjected to the subsequent analysis.

Finally, a single-nucleotide polymorphism (SNPs) analysis was conducted, for which 774 Colombian genomes were download of GISAID (ranged from 6 March 2020, until 21 March 2021) and compared with the Wuhan reference sequence (NC_045512), using the UGENE v.33.0 software [44].

### 2.5. Statistical Analysis

A descriptive analysis of the sociodemographic variables collated in the databases was performed. The quantitative variables were summarized in terms of means or medians and standard deviation or interquartile range, depending on their distribution. Qualitative variables were summarized in frequencies and proportions. Statistical analyses were carried out using R software [45]. For continuous values, normality hypotheses were evaluated using the Shapiro–Wilk test. All tests of significance, parametric or non-parametric tests, were two-tailed, and *p*-values <  0.05 were considered statistically significant. 

## 3. Results

### 3.1. Epidemiology and Geographical Distribution of SARS-CoV-2 

Epidemiological and genomic analysis was performed starting from the date the first case was detected in Colombia on 6 March 2020, through 6 March 2021. As of 6 March 2021, 2,273,245 positive cases were reported, of which 48.56% were males and 51.44% females, with a median age of 38 years (IQR 27–53). There was higher transmissibility amongst the young and middle-aged (20 to 39 years old, 44.39%), with an age-specific death tendency among persons aged 60 to 89 years (72.33%). Elderly patients (≥60 years old) had a cumulative mortality rate of 5188.70 individuals per 100,000 (Supplementary Table S1). According to the dynamics of COVID-19 cases observed and considering the dates on which certain measures and strategies were lifted and implemented by the Colombian government, two time periods were defined (Figure 1a). The first period (6 March to 10 August 2020) covers the period from the identification of the first case in the country and the adoption of the respective measures, until the lifting of the lockdowns and restriction measures. This date was chosen due to new public health measures that were taken by the national government to manage the pandemic in the country. On 10 August, the Colombian government decreed the PRASS strategy (Program for Testing, Traceback and Sustainable Selective Isolation), which was designed to improve the epidemiological containment of the pandemic, allowing to implement an economic reopening involving the lifting and easing of certain measures and restrictions. Thus, for example, within these new measures, different airports reactivated domestic flights and in subsequent days, the blockade and mobility restrictions in Colombia were lifted. This period also includes the first peak of cases presented in Colombia [35,46,47,48]. The second period (11 August 2020, to 6 March 2021) is delimited between the moment of the lifting of the measures and restrictions until 6 March 2021, which corresponds to the cut-off date, one year after the beginning of the epidemic in the country. This second period includes the second peak of cases presented in Colombia (Figure 1a).

Based on the analysis of currently available SARS-CoV-2 genome assemblies, we identified a greater number of genomes reported throughout March and April 2020 and at the beginning of the second peak of the pandemic countrywide (December and January 2021) (Figure 1b), overlapping with a higher number of identified lineages (Figure 1c). Although the number of genomes reported during the first peak of the pandemic was low, we noted that the diversity of lineages was probably higher, regarding other periods evaluated (Figure 1b); however, it is important to highlight the low number of genomes analyzed in this period and how this number may be underestimating or overestimating this diversity. Most of the lineages reported fell into the major lineage B (43/52), and B.1 was the most frequently reported (*n* = 250; 32%). An additional lineage of interest was B.1.420, which ranked third in frequency (*n* = 65; 8%) together with the lineages B.1.1 and B.1.1.348. Likewise, we observed that the largest populated centers in the country (Bogotá, Valle del Cauca and Antioquia) presented the highest number of reported genomes (*n* = 122, 70 and 145, respectively), as well as the highest diversity of lineages (16, 21 and 15, respectively) (Figure 1d). An exception to this was the Amazonian region, for which 108 genomes have been completed thus far, identifying 13 lineages, one of them the Gamma variant (P.1) with 19 genomes informed to date. 

**Figure 2:**
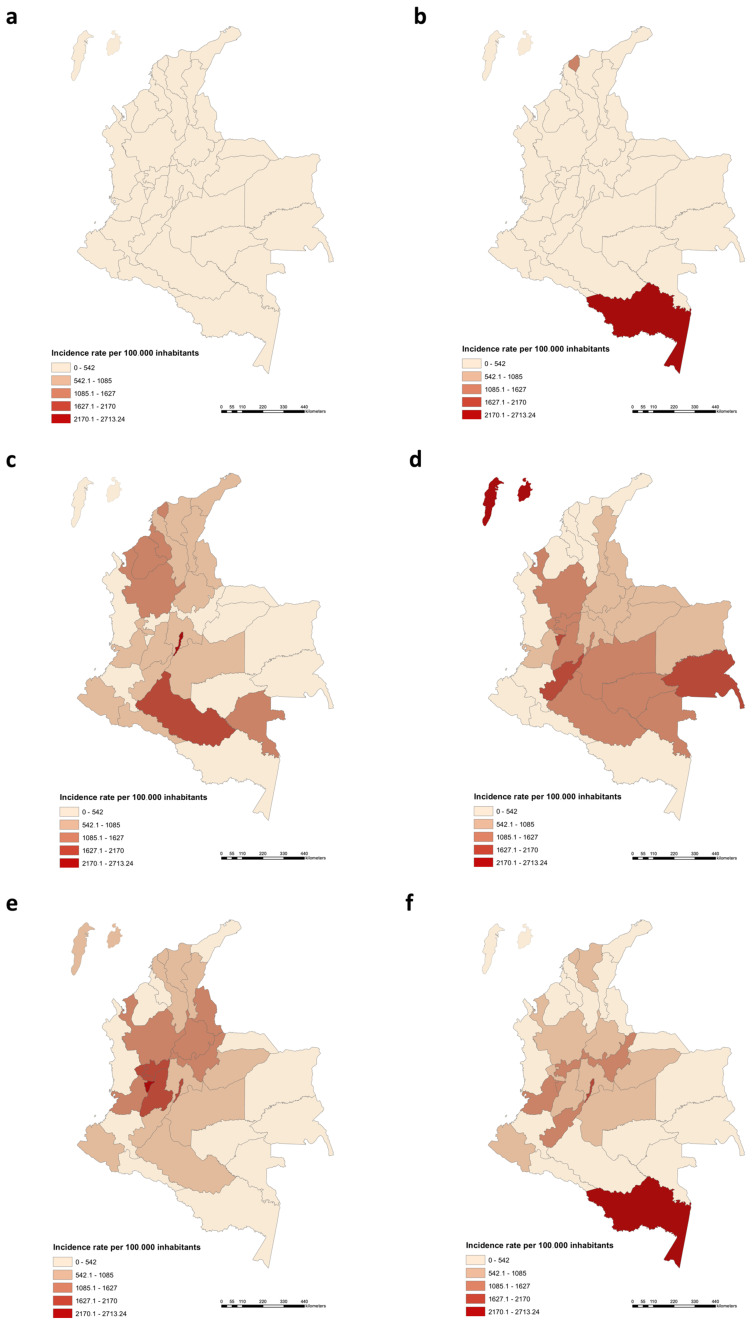
Geographical distribution of confirmed cases of COVID-19 in Colombia. Data was officially reported until 6 March 2021 (*n* = 2,273,245). We present results of the incidence per 100,000 inhabitants; this incidence value corresponds to the color intensity in the 2-month periods (**a**) March-April, (**b**) May–June, (**c**) July–August, (**d**) September-October, (**e**) November–December and (**f**) January–February (2021).

The analysis of the incidence rate per 100,000 inhabitants carried out across different regions of Colombia allowed visualizing the changes in the geographic distribution of cases as well as to evaluate how local and regional containment measures had a positive or negative impact on the behavior of the pandemic at a departmental level (Figure 2). The local governments decided to take measures such as confinements, mobility restrictions, prohibitions of entry and exit of individuals to the different regions, among others, which were decreed independently of what was established by the national government. The differential impact that these regional measures had can be seen in the dynamics and distribution of the cases in the different regions of the country (Figure 2 and Supplementary Data S1). The cumulative incidence during the timeframe March 2020 to March 2021 revealed that Bogotá (8063.0), Quindío (5482.71), Amazonas (5055.68), Antioquia (4922.35) and Tolima (4724.41) were the departments with the highest incidence rate per 100,000 people (Supplementary Data S1).

### 3.2. Phylogenomic Analysis and Nucleotide Diversity

**Figure 3:**
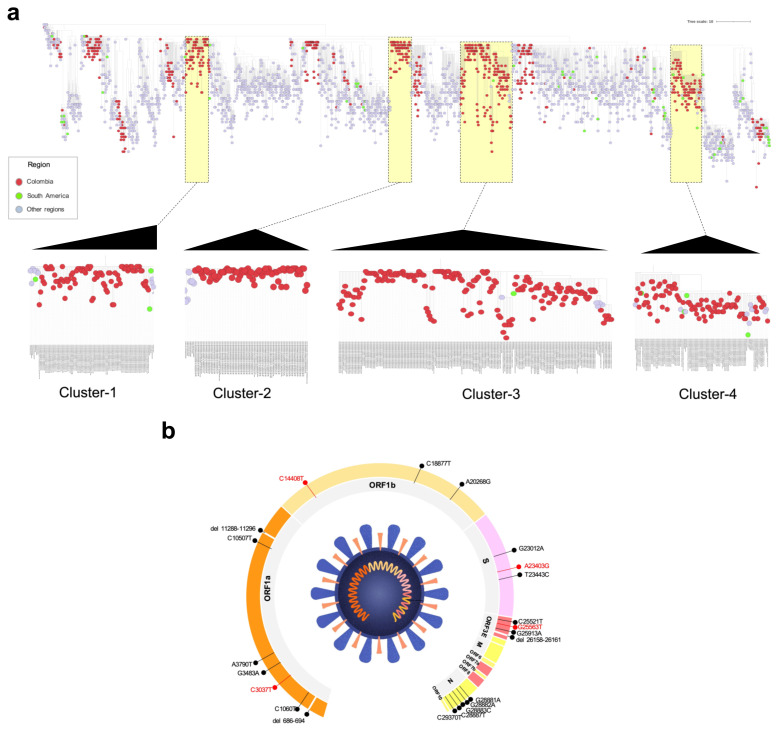
Phylogenetic analysis of global SARS-CoV-2 isolates and nucleotide diversity of the Colombian genomes. (**a**) Phylogenetic relationships between the 2623 genome sequences analyzed, which include the 774 Colombian genomes. (**b**) Position of each of SNP within the SARS-CoV-2 genome, to compare the 774 Colombian isolates and the Wuhan reference sequence (NC_045512). The SNPs found in more than 50% of the genomes analyzed are highlighted in red.

The phylogenomic relationships of the SARS-CoV-2 genomes from Colombia were analyzed in this study (*n*: 774) in context of a representative worldwide genome dataset (*n* = 1849) that included the first genome per lineage. The maximum likelihood reconstruction revealed a heterogeneous distribution of Colombian genomes (Figure 3a). Still, most of the Colombian genomes identified fell into four main clusters (C1–C4), defined as monophyletic clusters constituted, mainly by genomes of Colombian origin. C1 included 93 genomes, 86 from Colombia (92.5%), and the other 7 genomes from USA, Turkey, Sweden, Saint Martin and Bolivia. This cluster was closely related to genomes from other American countries, such as Argentina, Chile, Mexico and Australia, as well as European countries, such as France, Germany and Spain. C2 comprised 77 genomes, predominantly from Colombia (*n*: 71; 92.2%), with 6 genomes from the USA. C3 included 185 genomes where 179 were from Colombia (96.7%) and the remaining 6 from other origins (USA, Chile, Germany and Switzerland). Lastly, C4, with 109 genomes, incorporated 95 genomes from Colombia (92.5%) and 14 genomes from different countries, such as USA, Spain and others.

Finally, all 774 publicly available SARS-CoV-2 genomes from Colombia were used for single-nucleotide polymorphism (SNP) analysis (Figure 3b). Nineteen (19) shared polymorphic sites in more than 10 percent of the genomes analyzed were identified. Four mutations were the most representative, two located in ORF1b at positions 3037 (C/T) and 14,408 (C/T), one in the S gene at position 23,403 (A/G) and four in the ORF3 at position 25,563 (G/T). Additionally, two 9nt deletions (686–694, 11,288–11,296) and one 4nt deletion (26,158–26,161) were identified.

### 3.3. Effective Reproductive Number (R_t_) Estimation Model and Infection Dynamics

**Figure 4:**
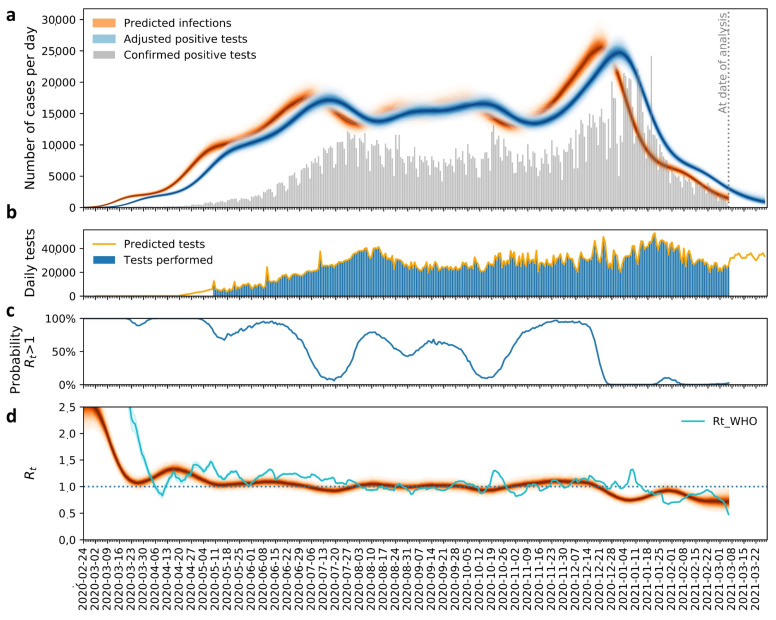
Epidemic model results. (**a**) Positive tests and infections: the bars represent the positive tests per day. The blue line corresponds to the positive tests corrected by the model, which adjusts for the number of daily tests. The red line is the infection events (cases) that will later become positive tests. The difference between the bars and the blue line corresponds to the positive tests inferred by the model that was not able to diagnose. The model has errors, so the blue and red lines are blurred. This graph predicts the evolution at 21 days. (**b**) Daily test: the blue bars correspond to the number of total tests performed. The yellow line are the tests that the model predicts will be performed in the future. (**c**) Probability *Rt* > 1: the blue line corresponds to the probability that the *Rt* is greater than 1. (**d**) *Rt*: Here, *Rt* is plotted for each point in time. The model has errors, so the red line is blurred. The *Rt* estimation from the use of the WHO model (EpiEstim) is also plotted in light blue.

Implementation of the *R_t_* estimation model [36] revealed several features pertaining to the dynamics of the pandemic in Colombia (Supplementary Data S2). The number of tests per day and predicted infections were calculated. The model also inferred the number of actual cases adjusted based on the volume of tests performed (Figure 4a and Figure S1). In addition, the predicted infection curve showed a delay when compared to the adjusted predicted positive, as expected based on the time window between time of infection and test reporting. We also observed an important shortcoming: the underreporting of positive tests due to operational constraints and case-capture oversight. Along these lines, the available data showed a median number of positive tests of 1300 (during the first period), while for the second period it was 7896, clearly depicting a higher number during the latter period (Mann–Whitney–Wilcoxon test, *p* < 0.05).

As described above, our model allowed estimating the adjusted positive tests. In this sense, it was evident that the median of the adjusted positive tests inferred was 6461, while for the second period it was 28,784, clearly higher than in the latter period (Mann–Whitney–Wilcoxon test, *p* < 0.05). Based on this and as predicted for the first period, Colombia had approximately a median of 5161 unperformed tests, while for the second period it summed up to a 20,888-test deficit. Furthermore, by using the number of tests per day, our model also permitted estimating prospectively the number of tests to be performed. Looking at the number of cases processed between 6 March 2020, and 6 March 2021, a total of 8,148,715 tests were completed countrywide with 21.5% performed during the first period and 78.5% throughout the second period. Viewed this way, our model estimated an average of 33,510 (±1787) tests per day that will be performed between 7 March and 27 March 2021 (Figure 4b and Figure S1).

By measuring the basic reproduction number value, our model made it possible to predict the *R_t_* dynamics at different timepoints (Figure 4d), as well as to determine the probability that the *R_t_* value may be greater than 1 at any given time (Figure 4c), which is important when assessing population heterogeneity. In order to compare the performance of our model against that implemented by the WHO [41], a data analysis was carried out also under this model (Figure 4d and Figure S1 and Supplementary Data S2 and S3). Interestingly, the results showed only slight variations in the *R_t_* dynamics among both models, which is mainly due to the application of the delayed distribution for the *R_t_* calculation in the Rt.Live model. By using the Rt.Live model, we estimated that the *R_t_* for Colombia throughout the first period of the pandemic had a median of 1.07 (0.97–1.19 CI 95%). Median *R_t_* values ranged from 2.13 (1.82–2.52 CI 95%) at the beginning of the pandemic through March 18, to a median of 1.27 (1.14–1.42 CI 95%) between 7 April and 29 April. Both time-periods coincided with the model’s highest probability estimation for obtaining an *R_t_* greater than 1 (Figure 4c,d). For the second period, the median was 0.99 (0.88–1.10 CI 95%). The higher values were observed throughout 19 November to 11 December, where the *R_t_* scores were above the epidemic threshold, with a median of 1.09 (0.98–1.20 CI 95%), to later decline. Over this time range, the highest probability estimation for obtaining an *R_t_* above 1 was also recorded (Figure 4c,d). After 20 December, the *R_t_* remained steady below 1. Statistically significant differences relating to *R_t_* were observed among both periods (Mann–Whitney–Wilcoxon test, *p* < 0.05) (Figure 4d). We also determined the *R_t_* and assessed the dynamics for all the different departments and regions in Colombia, where we can see specific patterns (Figure S1 and Supplementary Data S2 and S3). As expected, variation amongst regions was evident, highlighting the potential differences in local–regional containment efforts (as an obligatory lockdowns and quarantines), social precariousness and variance in healthcare infrastructure. Based on this, it becomes evident that in vulnerable regions such as Chocó, Amazonas and Guainía, among others, testing capacity is limited, therefore leading to a significant underreporting of cases (Figure S1 and Supplementary Data S2 and S3).

## 4. Discussion

In this study, we provide an assessment of the epidemiology of SARS-CoV-2 in Colombia, following the report of the first COVID-19 case detected in early March 2020 and its progression throughout the pandemic based on publicly available data. We have implemented a novel epidemic model combining genomic surveillance interpreted in the context of available epidemiological data.

Similar to other Latin American countries, such as Brazil, Chile and Ecuador, [2,13,49], our results confirm that the highest rate of SARS-CoV-2 infection in Colombia occurs mainly in younger age groups (median age of 38 years), and that most fatal cases affect the elder population. These results contribute to the understanding of the epidemiological behavior of SARS-CoV-2 across different population groups and emphasize the need for prioritizing vaccination in particularly vulnerable at-risk groups, such as the elderly (Table S1) [34]. In addition, our analyses on the geographic distribution of SARS-CoV-2 revealed significant heterogeneity, with some regions of Colombia being heavily affected, particularly after lifting restrictions on August 2020 (Figure 2 and Supplementary Data S1). The most likely contributing factors influencing the high density of cases in these regions include links to travelers returning from other epidemic areas and implementation of ineffective intervention measures as well as poor social compliance [10,12,18,29]. Other causes include the highly porous nature of the borders with neighboring countries (Brazil and Peru)—favoring illegal migration—social inequality, and poor access to healthcare services [3,29,50]. 

Like other Latin American countries, a large proportion of Colombia’s population is employed in the informal sector, relying on jobs that cannot be performed at home, and which would undoubtedly favor from the re-opening of the economy. However, lifting mobility restriction policies in the midst of the pandemic may prove deleterious in containing local epidemics and potentially fueling dissemination to other regions. In this context, we consider that reopening should be a gradual process in which clear strategies should be defined in order to decrease the impact of the outbreak and prevent similar scenarios as those observed in some European countries [51], where transmission has reignited in many areas.

Results obtained from analyzing all SARS-CoV-2 genome assemblies revealed an exponential growth in the number of circulating lineages across the country; this since the last large-scale genomic study performed in Colombia [15]. Of the 774 available sequences, 52 PANGOLIN lineages were recorded, including the newly emergent variant Gamma (lineage P.1) recently identified in Brazil (Figure 1c,d), which has been linked to increased transmissibility and infectivity [4]. In Colombia, the Gamma (P.1) and B.1.195 lineages have been found to circulate exclusively in the Department of Amazonas, which shares borders with Peru and Brazil (in the year period analyzed). Additionally, we found that some departments harbored a greater diversity of lineages coinciding with those having the highest population rates across the country (Figure 1), probably due to increased traveling and enhanced local community transmission. Altogether, our results along with the identification of other shared lineages (B.1, B.1.111 and B.1.129), the presence of different clusters and the close phylogenetic relationship amongst different countries (e.g., Colombia, Chile, USA and Europe) strongly suggest multiple SARS-CoV-2 introductions into Colombia from other countries. It is possible that virus importation occurred because of lifting of restrictions and re-opening of domestic and international flights (Figure 1a), as well as migratory mobility favoring dissemination from neighboring regions.

Further, the epidemic model implemented in this study highlighted some important aspects associated with the dynamics of the pandemic in Colombia. One of the most important features was unveiling the country’s diagnostic response (Figure 4a and Figure S1). During the initial epidemic wave, the total tests per confirmed case performed was 3.55, increasing to 3.58 throughout the second wave. This is in sharp contrast with other South American countries, such as Uruguay, where their initial high testing capacity (233.7 people tested for each confirmed case of COVID-19) allowed a timely implementation of measures preventing viral spread and possibly more variants into its healthcare system [52]. Although Colombia has expanded its daily testing capacity, utilizing other non-PCR-based tests, these are still insufficient and with limited sensitivity [53,54], leading to underreporting and affecting estimates inferred by epidemic models that impact decision-making to inform policy recommendations (Figure 4b and Figure S1).

Another relevant finding had to do with the estimation of the Effective Reproductive Number (*R_t_*) and its relationship with the different public health and non-pharmaceutical interventions implemented to control virus spread. Studies on epidemic models carried out in Europe and several Asian countries throughout the first months of the pandemic provide ample evidence that non-pharmaceutical measures, such as school closures and social distancing, have been fundamental in reducing viral transmission and the *R_t_* value [17]. Early in the pandemic, the SARS-CoV-2 epidemic in Spain displayed an *R_t_* of 2.83 to 2.9, meaning that each case spread to an average of 2 to 3 other exposed persons [17]. Similar findings were reported for Latin America where early evaluations on the impact of transmission through the first 10 days of the pandemic revealed an exponential growth with an *R_t_* greater than 2 [16,55]. Amongst these, Brazil was the country that exhibited the highest *R_t_* at the beginning of the pandemic (*R_t_* of 2.96), with Colombia scoring an *R_t_* of 2.67, consistent with the results obtained in this study, which is now starting to level-off throughout specific regions (Figure 4c,d and Figure S1) [16].

We observed a gradual decrease in the number of cases towards the end of each period as well as a decline in intensive care unit bed occupation through the end of January 2021. However, it was evident that during the reopening periods (August 2020 and December 2020), relaxing of measures, such as home confinement (quarantine), social distancing and travel restrictions, translated into an increase in the number of cases and circulating lineages. Additionally, and as predicted by our model, variations in *R_t_* during these periods coincided with the highest probabilities of values greater than 1, increasing the chances for potential epidemic spread (Figure 4c). Nevertheless, the effectiveness of strict public health interventions implemented by local and regional governments, particularly during mid-late January 2021, had a positive effect on reducing the rate of transmission as observed with the reduction in the number of positive cases (Figure 1a) and a decline in *R_t_* value (Figure 4a and Figure S1).

Still, these results should be put into context considering additional factors, such as COVID-19 mortality, ICU admission and occupancy, as well as the overall number and type of tests performed, amongst others. For example, the number of COVID-19 cases have steadily increased following the peak of cases observed during the second period to date [56]. In addition, some degree of interpretation bias should be considered when examining a decrease in cases. For instance, an increase in antigen testing carries the intrinsic risk of generating false-negatives, mainly in asymptomatic and pre-symptomatic people, thus shadowing the number of true-positive cases. Another factor to consider is the delay in case reporting and other lagging epidemic indicators, which make it difficult to model and infer transmission dynamics. 

Determining the effectiveness of containment measures, for example, from the construction and evaluation of epidemic models, such as the one implemented in the present study, is crucial for public health decision making. Considering the above, it is essential to comprehend that an implementation of control measures not only requires timeliness from the government’s point of view, but also compliance by the population. It is evident that in Colombia the lack of compliance of the population has contributed to a greater negative impact, as evidenced in other countries [17,18,19], which is why better regulation, verification and control by government entities is required. Control strategies implemented in Colombia during the first year of the pandemic have been based on non-pharmaceutical measures since vaccination started only on 17 February 2021. These measures have been based primarily on mobility restrictions and social distancing [35,47]. However, the observed pandemic dynamics allow correlating the increase in identified cases and cases inferred by the model with the lifting of distancing measures, opening of airports and general relaxation of restrictions. Relaxation of control events may lead to a subsequent increase in new cases, facilitating spread and potential emergence and dissemination of novel genetic variants and the presentation of new peaks of greater magnitude [12,17,57]. Likewise, it is very likely that the population’s perception of risk in relation to the magnitude of the pandemic, which has been shown to be positively correlated with compliance with the measures, will vary over time [19,21,22,37]. For this reason, in certain periods of time, the low compliance with the measures defined by the government has become more evident, which can be reflected in the increase in the number of cases (Figure 1a and Figure 4). 

The complexity in the response of the government, added to the low compliance of the measures by the population, play a fundamental role in the dynamics of the pandemic, which is evident in the model implemented [12,16,18,37,58]. Until massive vaccination is widely fulfilled, key preventive measures, such as social distancing, use of personal protective devices, universal symptom survey and home confinement for positive cases, must continue in order to keep *R_t_* < 1, until a significant portion of the population achieves immunity. Measures taken by the different governments have made it possible to reduce the impact of transmission. However, many low- to middle-income countries in Latin America are still facing difficulties due to their fragile health systems [3,59,60,61]. Colombia has been experiencing great challenges given its political and social crisis, marked by social inequality, internal violence, and a lack of investment in healthcare infrastructure, all of which have steered to a delayed vaccination campaign. 

There is a limited number of studies combining the use of genomic analysis and epidemiological data to assess transmission dynamics. In this study, we followed this coupled approach in order to provide new insights into the introduction and spread of SARS-CoV-2 in Colombia. We report circulation of 52 lineages. In general, B/B.1.195 seems to have established and driving most of local transmission throughout the country. The observed peak in lineage diversity through the months of July and September coincides with the relaxing of contention measures and possible introduction of variants from international travel arrivals. In addition, the increase in sequencing capacity nationwide could be an additional contributing factor, which without a doubt has served to reveal a striking regional lineage diversity. A potential limitation to this study is the significant sample bias due to uneven genome sampling and/or overrepresentation of targeted study areas. 

The severe impact and time course of the SARS-CoV-2 pandemic has been highly variable across the globe. As the pandemic continues to unfold, some countries have succeeded in their efforts to mitigate spread. Others, like many South American countries, including Colombia, are still facing many challenges. For example, the emergence and rapid spread of variants such as Gamma (P.1), Alpha (B.1.1.7) and Delta (B.1.617) in South America has translated into a new raise in case numbers and a prolonged epidemic course. Linking genomic and epidemiologic data has proven to be the most useful tool to dissect the intricacies of transmission dynamics. However, we must emphasize that there are always limitations to epidemiological data and that no model can integrate all essential details, particularly given the multidimensional nature of an evolving pandemic. Herein, we have framed an epidemic model to serve as a basis for estimating the incidence of infection, and which mathematically demonstrates how modeled transmission dynamics translates into effective infection indicators by incorporating probability distributions for indicator-specific time lags from infection. However, we must not overlook the heterogeneity and the many other potential drivers influencing specific contexts, such emergence of novel variants. Therefore, it is necessary to apply epidemic models that help characterize and anticipate the course of epidemics by carrying out efficient genomic surveillance to facilitate our understanding of the transmission dynamics as well as to help implement an integrated approach to mitigate transmission and reduce the burden of morbidity and mortality associated with COVID-19. 

This is the first study from Colombia integrating both genomic and epidemiologic contexts in an effort to provide a comprehensive assessment to guide public health actions and help prepare for potential future pandemics. The information obtained here is highly relevant for understanding the dynamics of the pandemic in Colombia from an epidemic model and genomic surveillance during the first year of the pandemic. This study allows identifying the difficulties and limitations that the country has had in response to COVID-19, from diagnostic, public health, and health infrastructure perspectives, among others, providing information that will be useful not only to adapt the system to this pandemic but also potential future events. Likewise, this scheme can serve as a basis for evaluating the behavior of the pandemic based on previously implemented measures and also to monitor the impact of these measures together with the vaccination process. Our results should be a first step for deploying future strategies to improve the control and prevention of the pandemic. In light of this, some aspects that the Colombian government has improved so far are their genomic surveillance, diagnostic capacity and vaccination program. It is clear how various studies have sought to understand how the measures for the control of the pandemic are efficient to different degrees according to the social, economic and health contexts [12,16,19,20,37,59,62]; therefore, models, such as the one implemented here, provide essential data for decision making by national governments. As in any epidemic model, there are limitations related to the quality and timeliness of the data, mainly in the last few weeks reported. However, the methodological approach carried out shows great consistency with other models, as with the one compared in the present study, and allows its reproducibility.

## 5. Conclusions

In this study, we have implemented an epidemic and genomic surveillance model analysis, which together provide new insights into the introduction and spread of SARS-CoV-2 in Colombia during the first year of the pandemic. Within this period of time, the circulation of 52 lineages was identified, B/B.1.195 possibly being the one responsible for most of the local transmission throughout the country; however, a probably greater diversity of lineages was evidenced in periods of time when the relaxation and lifting of containment measures and of restriction of international travelers occurred. A possible limitation of this study in this regard is the significant sample bias due to unequal genome sampling and/or overrepresentation of the selected study areas. Likewise, it is clear how linking genomic and epidemiological data has proven to be the most useful tool to dissect the intricacies of transmission dynamics. Despite the inherent limitations that are always present in any model related to epidemiological data, an epidemic model has been implemented that serves as a basis for estimating the incidence of infection and mathematically demonstrates how the modeled transmission dynamics translate into effective indicators of infection that provide essential information for decision making. This model allowed determining broad difficulties and deficiencies in the management of the pandemic in Colombia, also demonstrating that the inappropriate lifting of restrictions and the relaxation of containment measures can lead to the presentation of subsequent peaks of greater magnitude and severity. Therefore, it is essential to apply epidemic models that help to make projections of the course of a pandemic, such as the current one. This, strengthened by the information obtained through efficient genomic surveillance, will facilitate the understanding of the dynamics of transmission and will contribute to the direction and implementation of effective measures aimed at mitigating transmission and reducing the burden of disease and mortality associated with COVID-19. This is the first study in Colombia that integrates the genomic and epidemiological contexts in an effort to provide a comprehensive assessment to guide public health actions and help prepare for potential future pandemics.

## Data Availability

All data are available in the main text or the Supplementary Materials.

## Supplementary Materials

The following are available online at https://www.mdpi.com/article/10.3390/vaccines9080837/s1, Figure S1 (Zip) Rt.live graphic model results for each region of Colombia, Table S1: Characteristics of laboratory-confirmed COVID-19 cases, Supplementary Data S1: Incidence rates per 100,000 population by region, Supplementary Data S2: Results of the Rt.live model, Supplementary Data S3: WHO model results.
