# Supplementary figures and images for: Evolution and Epidemic Spread of SARS-CoV-2 in Colombia: A Year into the Pandemic
Source: Vaccines (Basel). 2021 Jul 30;9(8):837. doi: 10.3390/vaccines9080837 (PMC8402472; doi:10.3390/vaccines9080837)

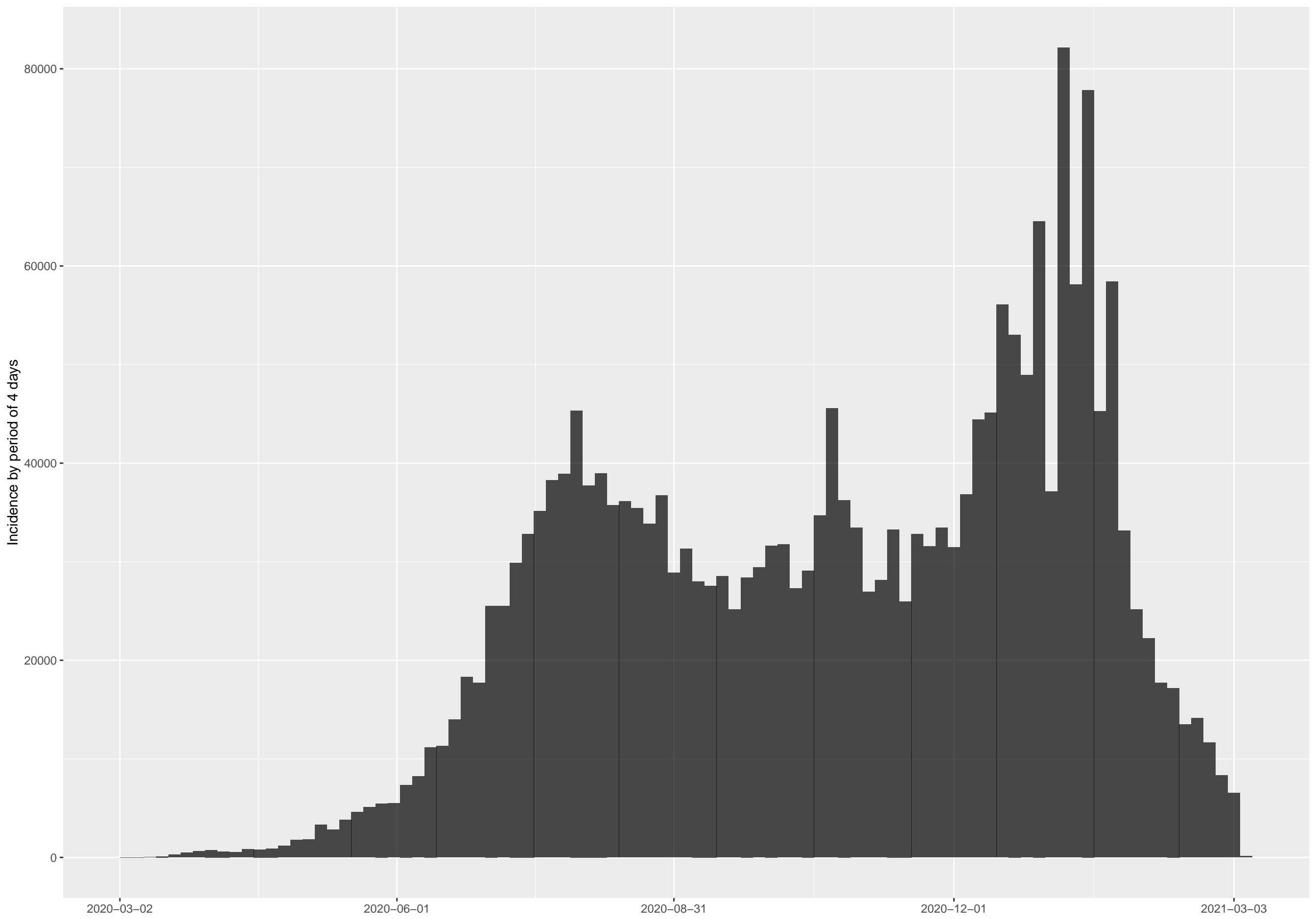

Supplement: Supplementary file 1 [file vaccines-09-00837-s001.zip › Supplementary_material/Supplementary Data S3/incidence_plot/WHO_model_incidence_ all .pdf]

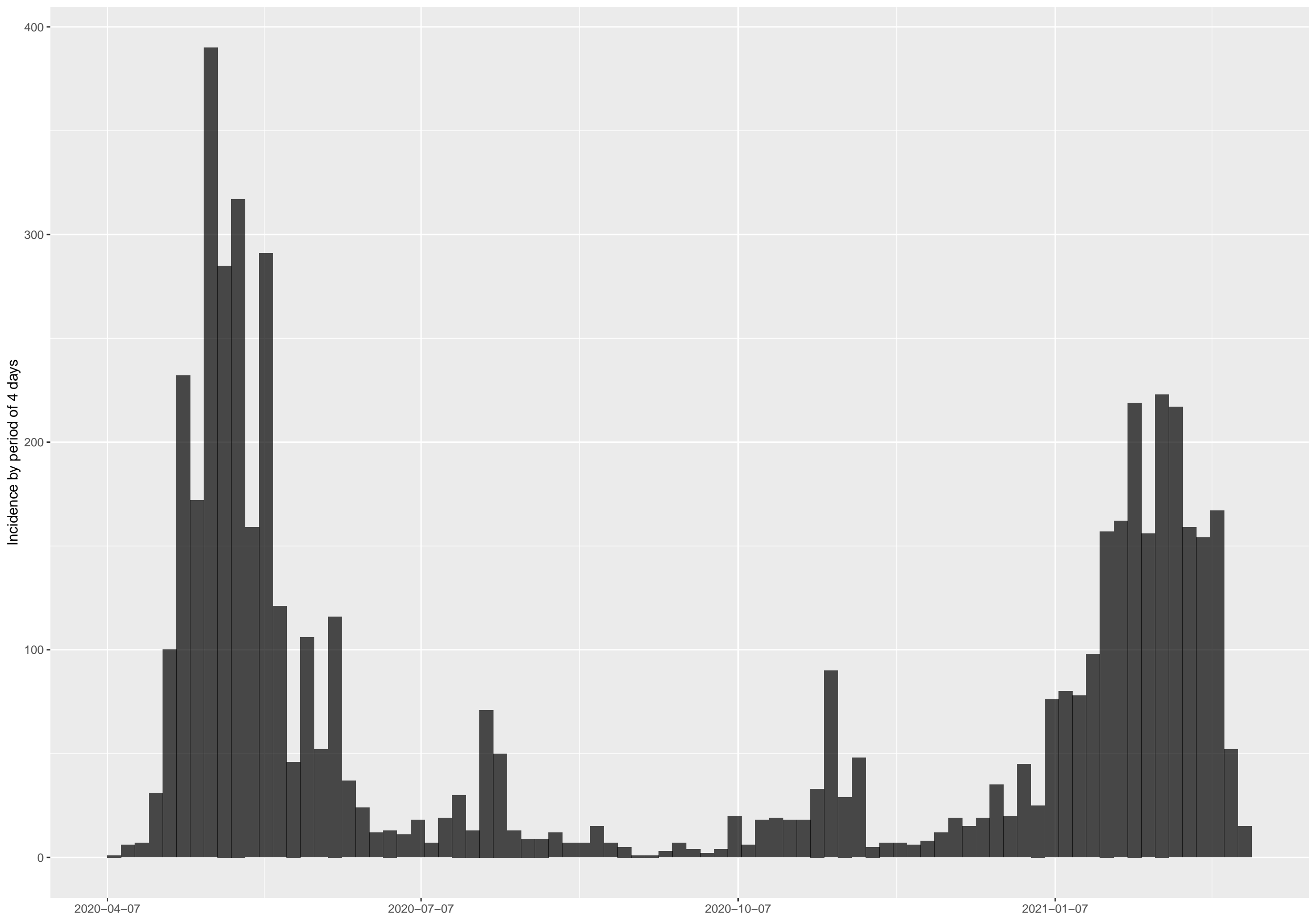

Supplement: Supplementary file 1 [file vaccines-09-00837-s001.zip › Supplementary_material/Supplementary Data S3/incidence_plot/WHO_model_incidence_ AM .pdf]

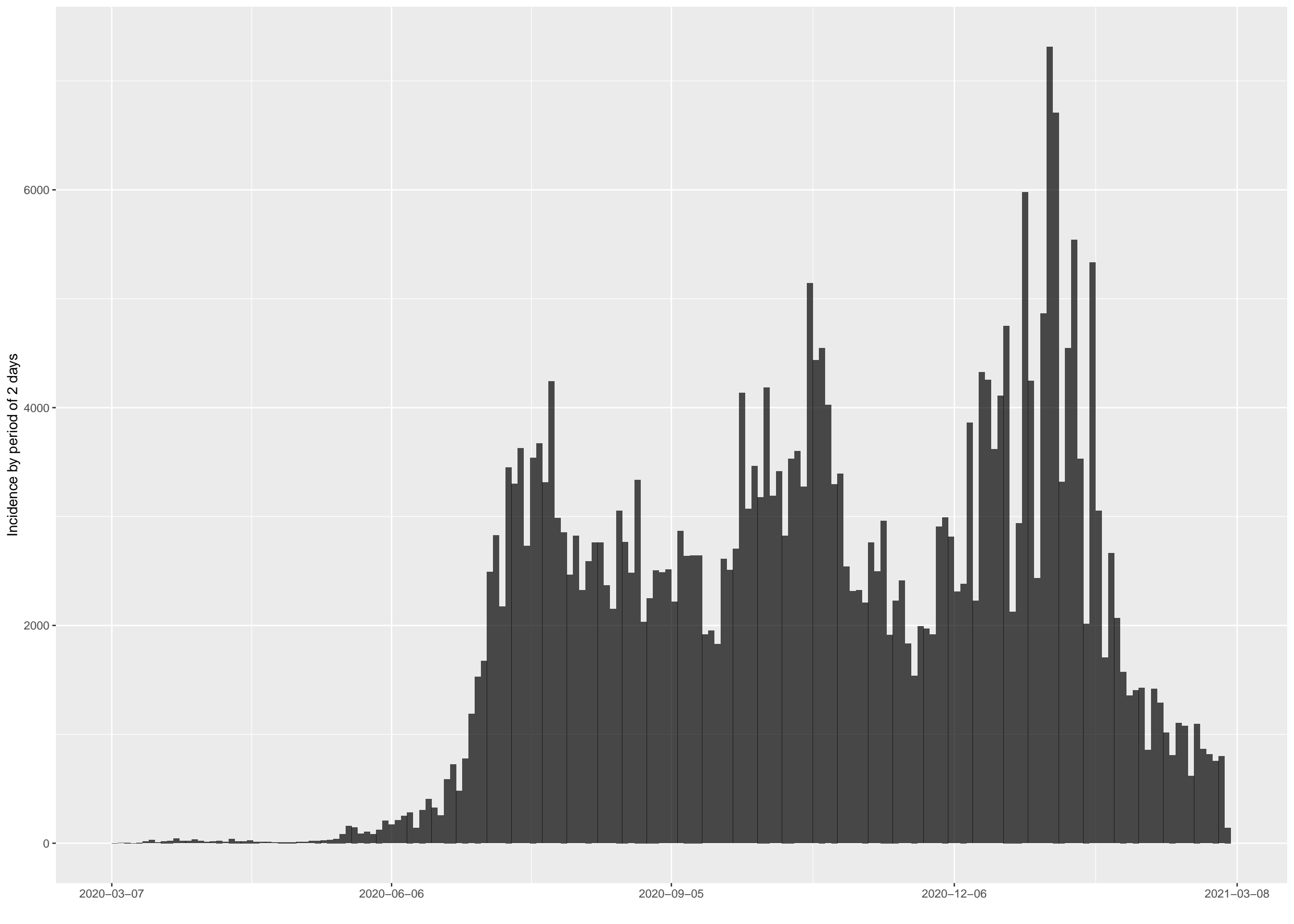

Supplement: Supplementary file 1 [file vaccines-09-00837-s001.zip › Supplementary_material/Supplementary Data S3/incidence_plot/WHO_model_incidence_ AN .pdf]

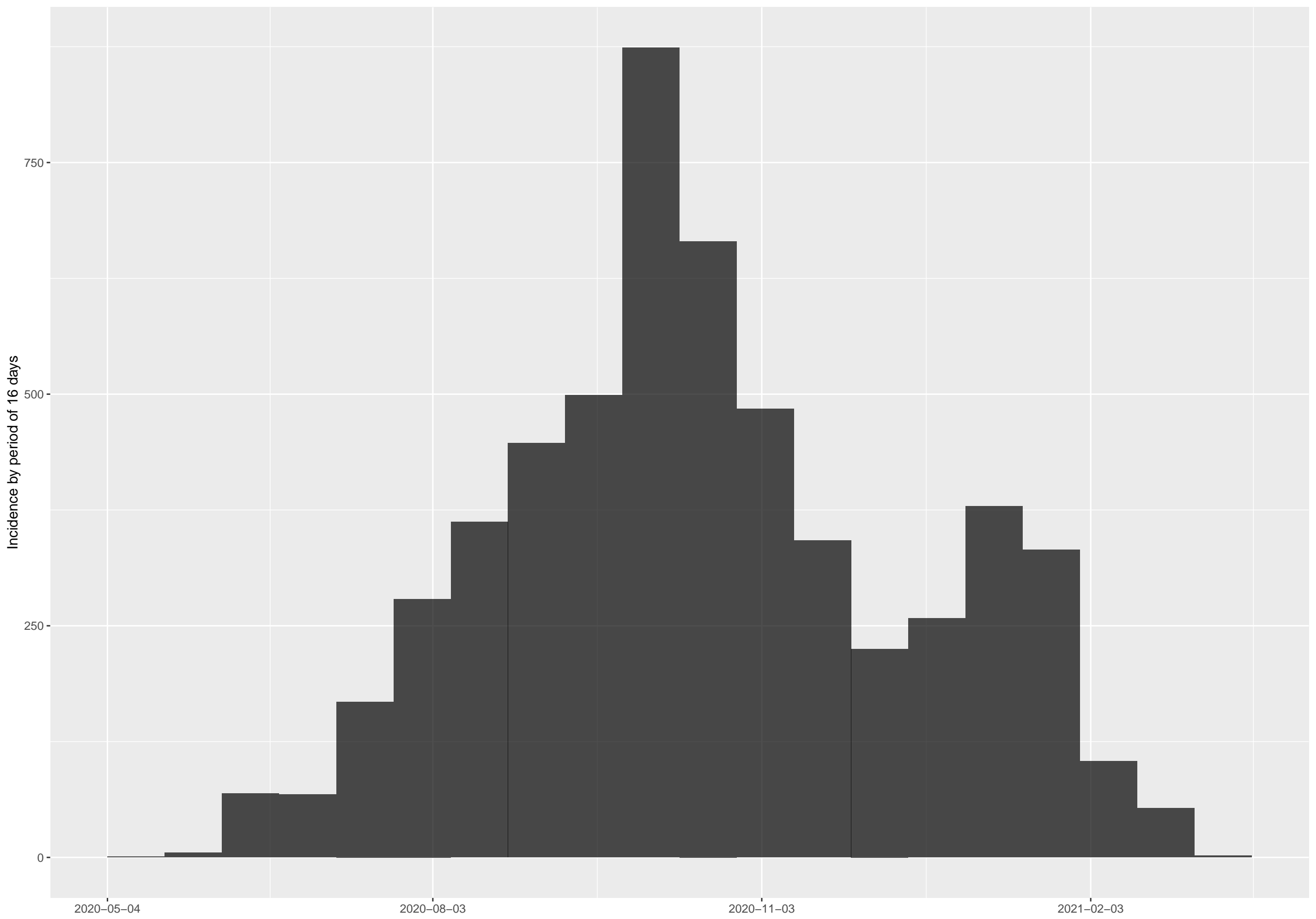

Supplement: Supplementary file 1 [file vaccines-09-00837-s001.zip › Supplementary_material/Supplementary Data S3/incidence_plot/WHO_model_incidence_ AR .pdf]

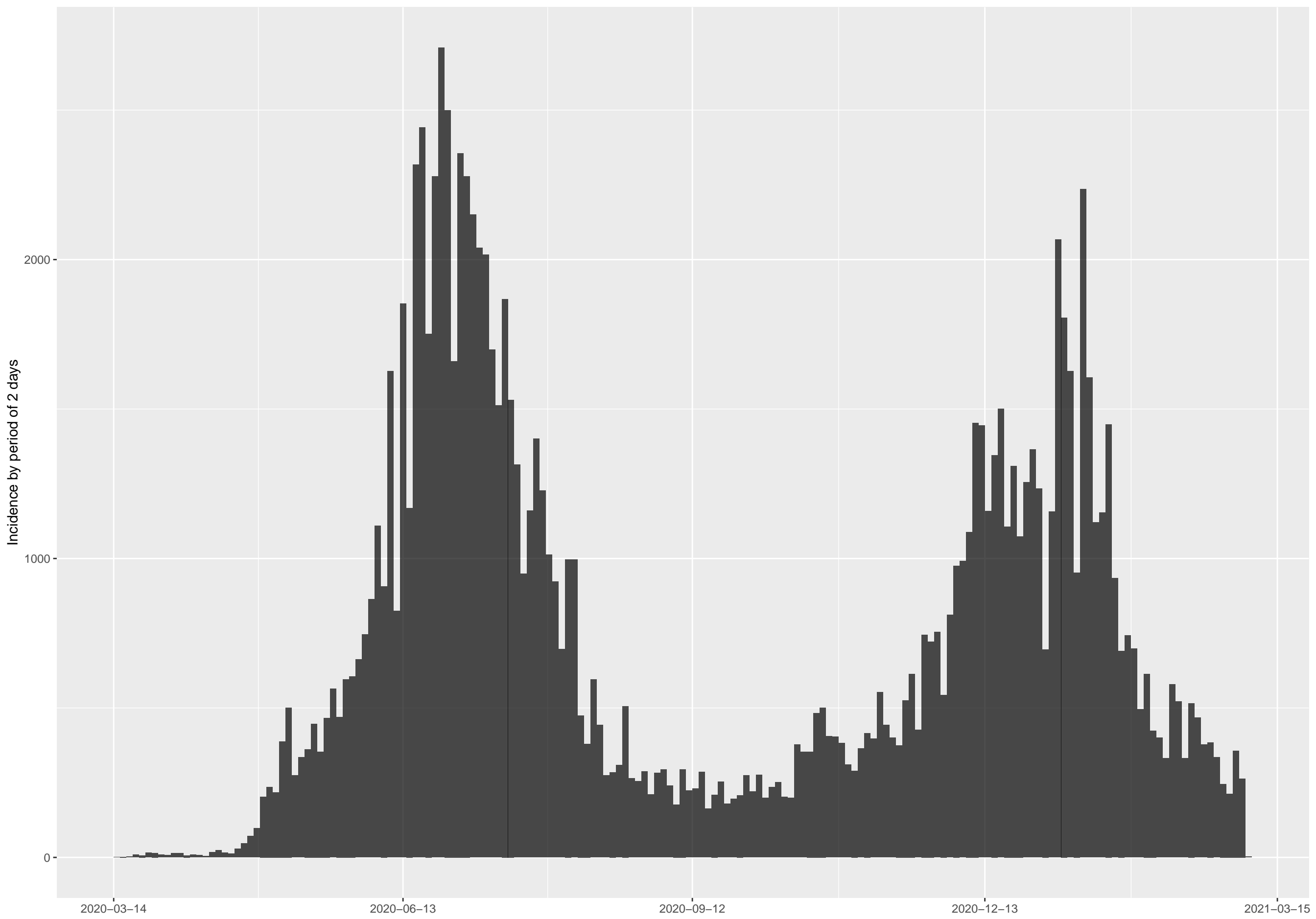

Supplement: Supplementary file 1 [file vaccines-09-00837-s001.zip › Supplementary_material/Supplementary Data S3/incidence_plot/WHO_model_incidence_ AT .pdf]

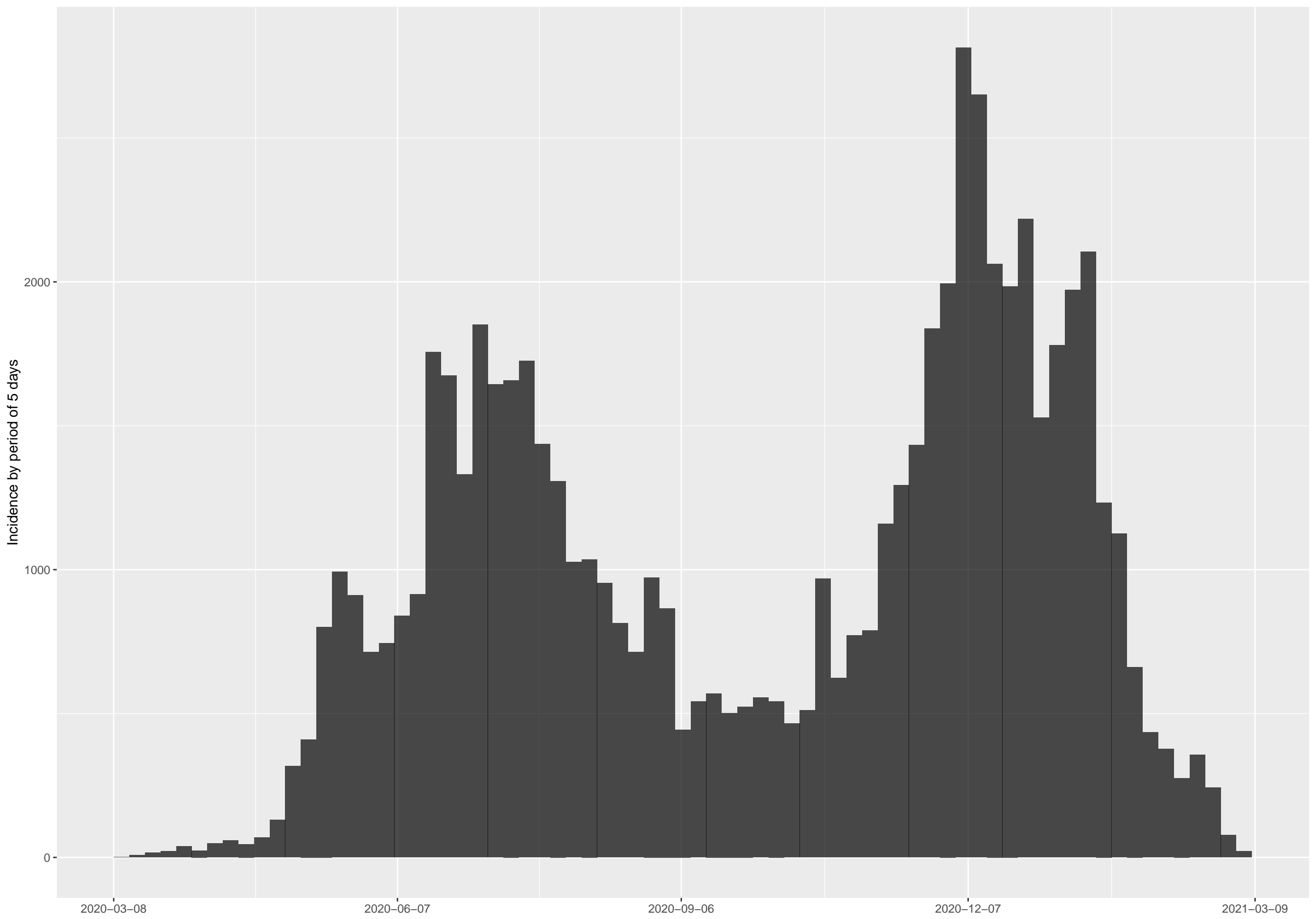

Supplement: Supplementary file 1 [file vaccines-09-00837-s001.zip › Supplementary_material/Supplementary Data S3/incidence_plot/WHO_model_incidence_ BL .pdf]

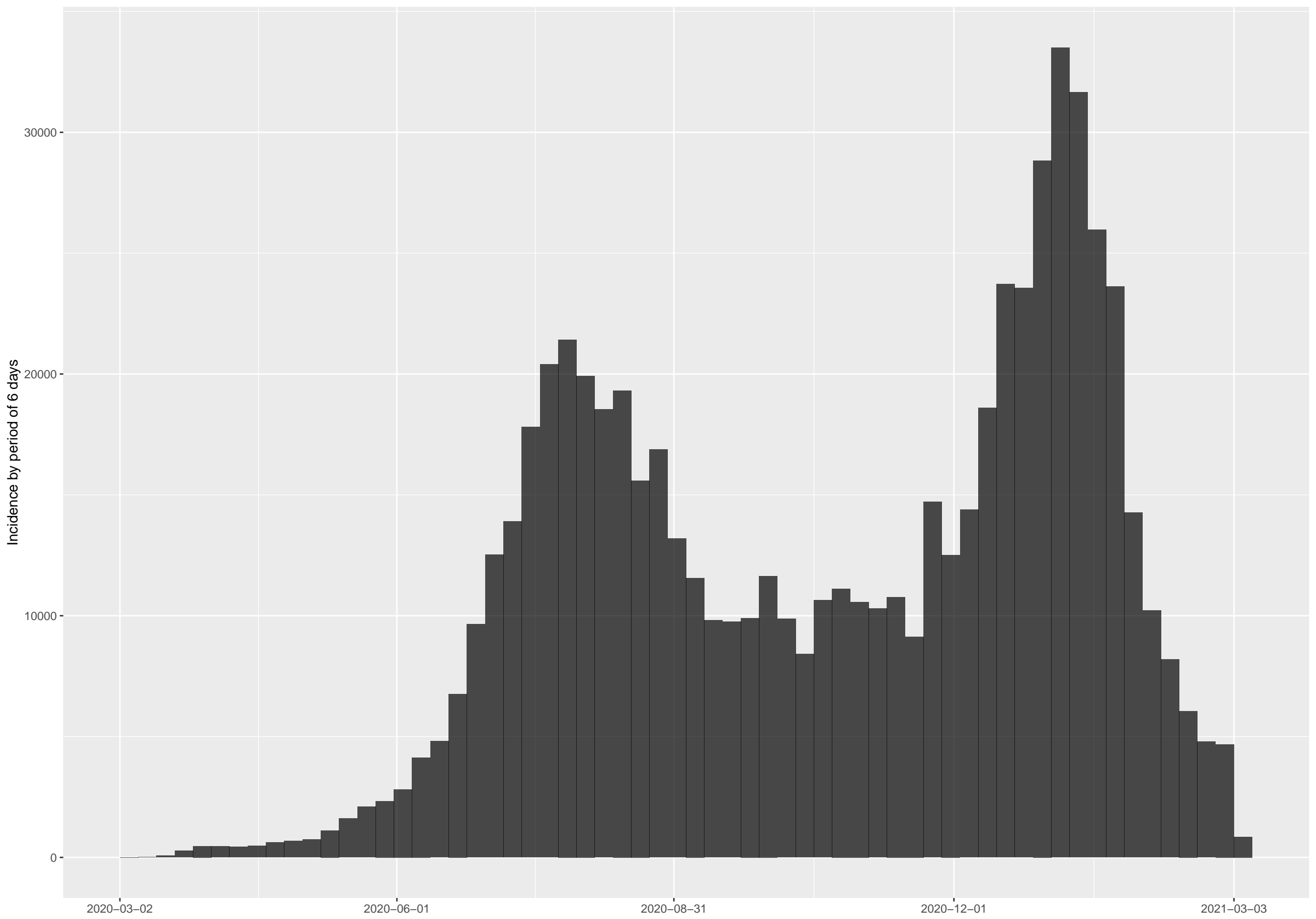

Supplement: Supplementary file 1 [file vaccines-09-00837-s001.zip › Supplementary_material/Supplementary Data S3/incidence_plot/WHO_model_incidence_ BO .pdf]

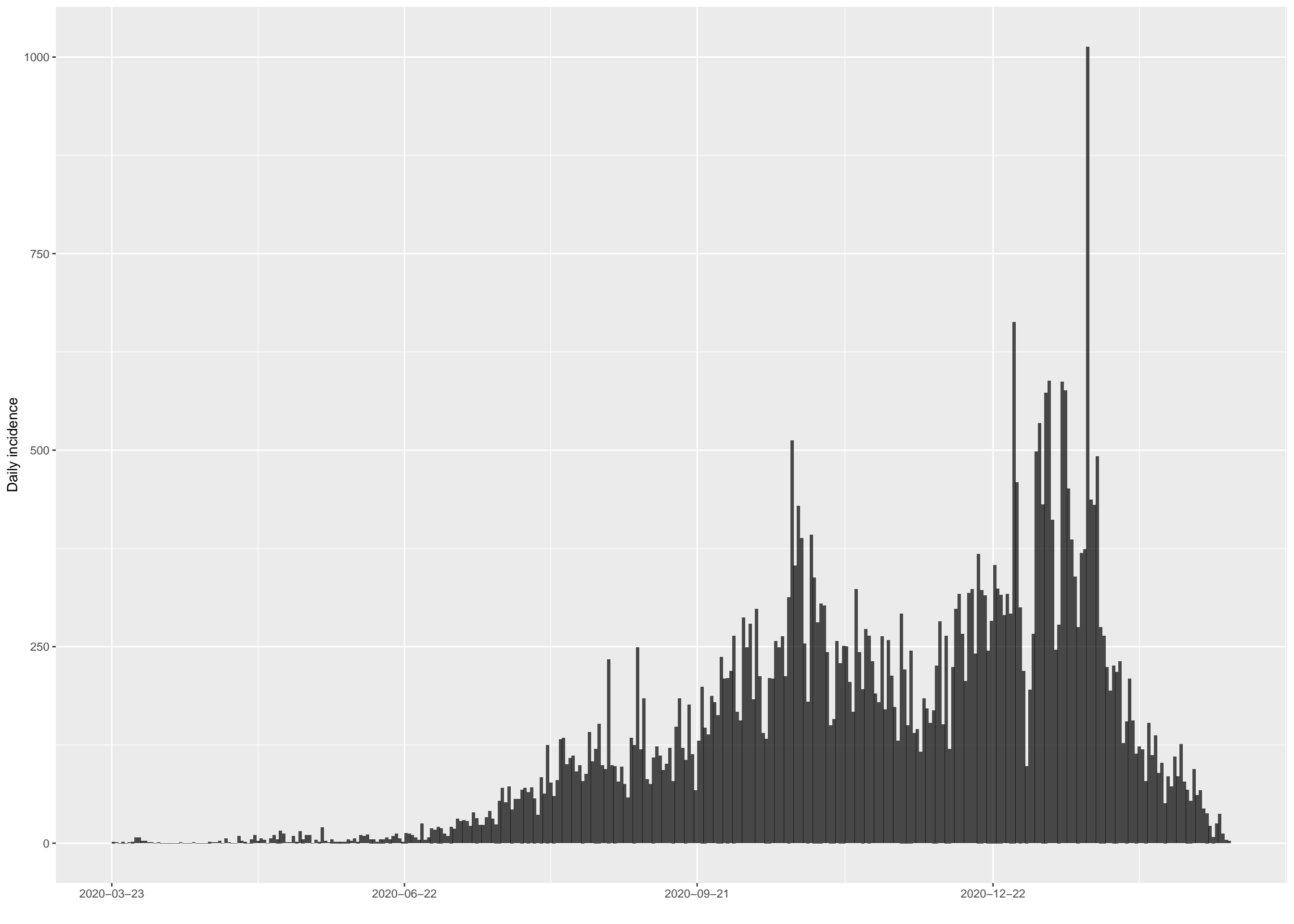

Supplement: Supplementary file 1 [file vaccines-09-00837-s001.zip › Supplementary_material/Supplementary Data S3/incidence_plot/WHO_model_incidence_ BY .pdf]

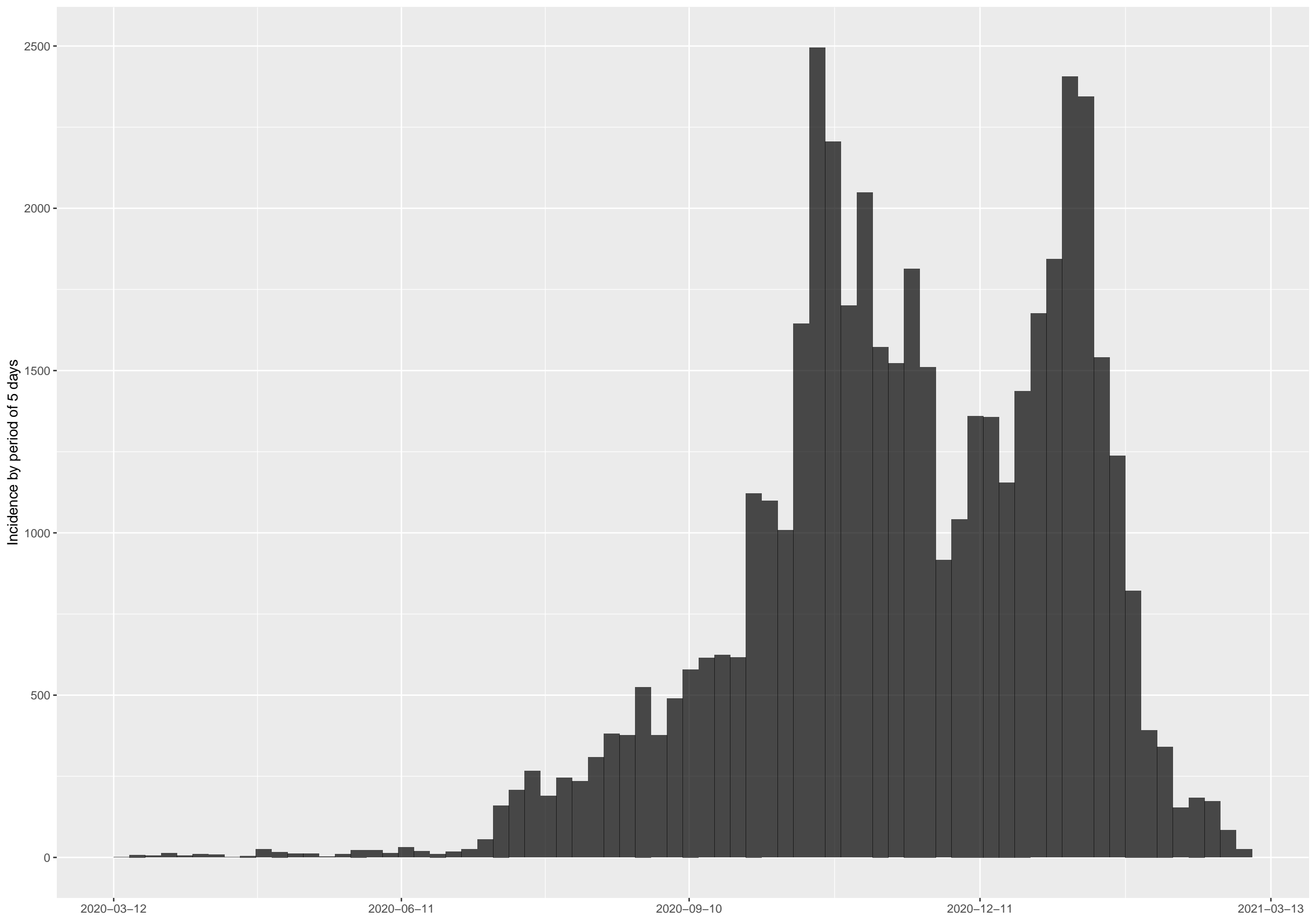

Supplement: Supplementary file 1 [file vaccines-09-00837-s001.zip › Supplementary_material/Supplementary Data S3/incidence_plot/WHO_model_incidence_ CA .pdf]

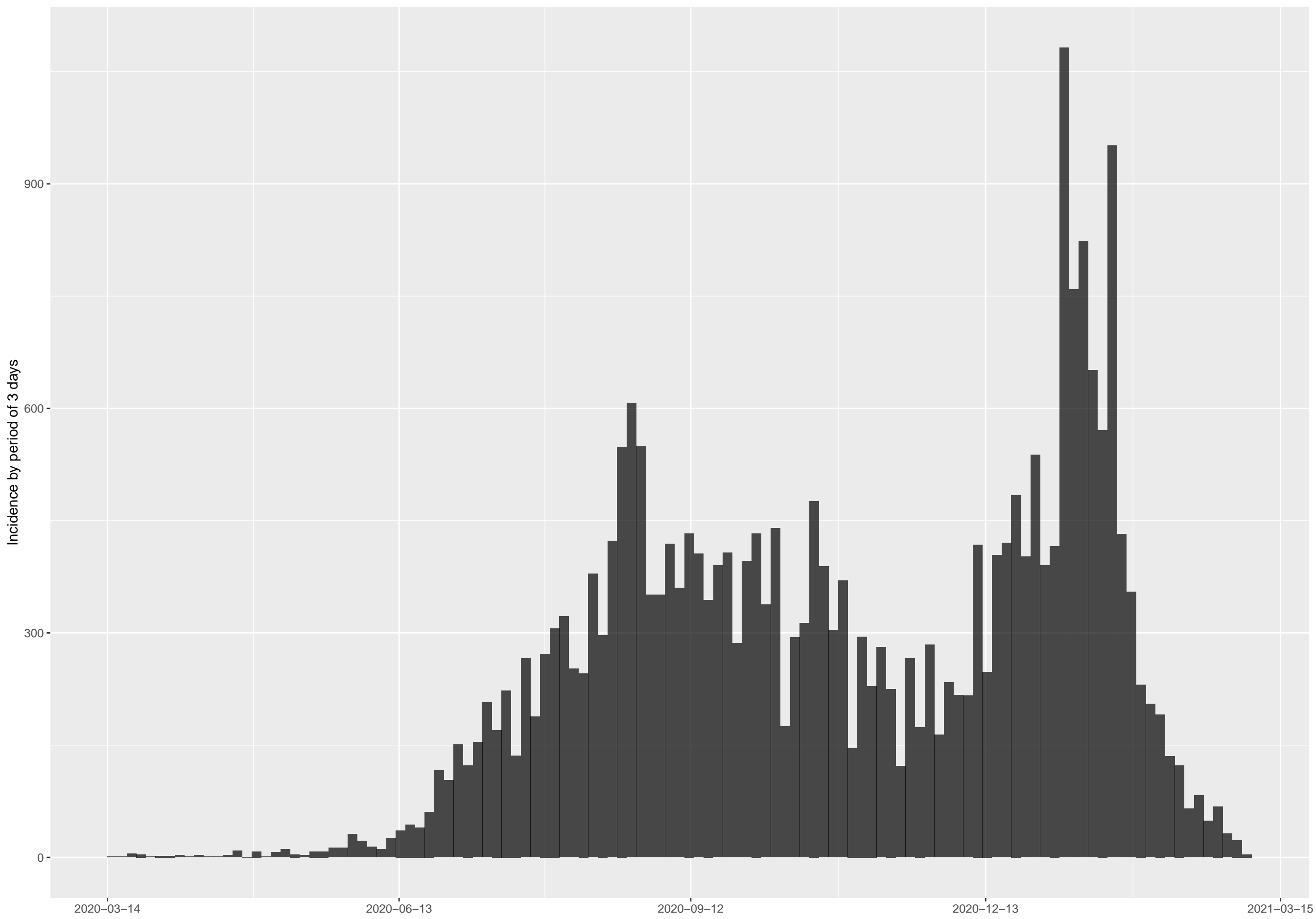

Supplement: Supplementary file 1 [file vaccines-09-00837-s001.zip › Supplementary_material/Supplementary Data S3/incidence_plot/WHO_model_incidence_ CC .pdf]

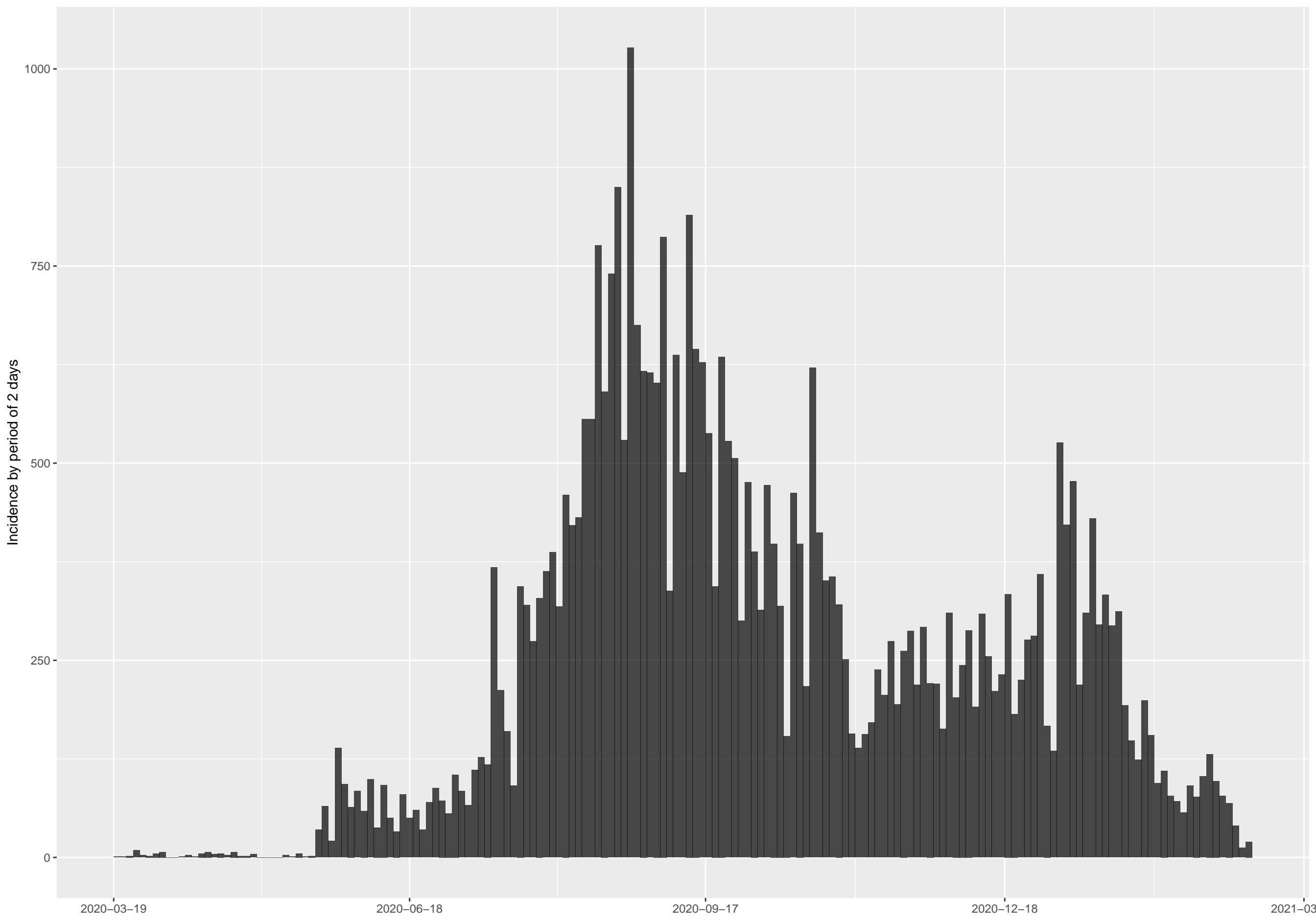

Supplement: Supplementary file 1 [file vaccines-09-00837-s001.zip › Supplementary_material/Supplementary Data S3/incidence_plot/WHO_model_incidence_ CE .pdf]

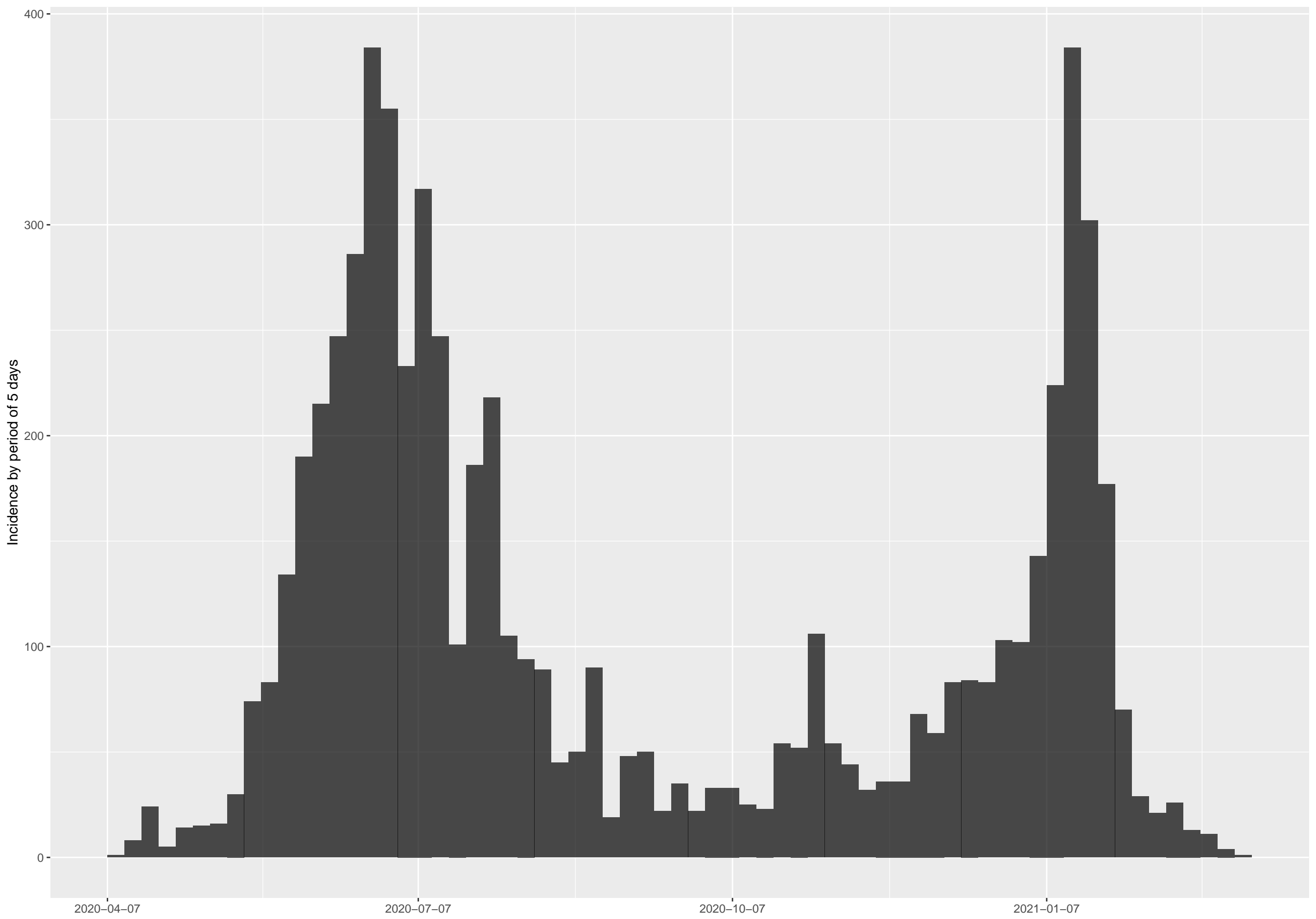

Supplement: Supplementary file 1 [file vaccines-09-00837-s001.zip › Supplementary_material/Supplementary Data S3/incidence_plot/WHO_model_incidence_ CH .pdf]

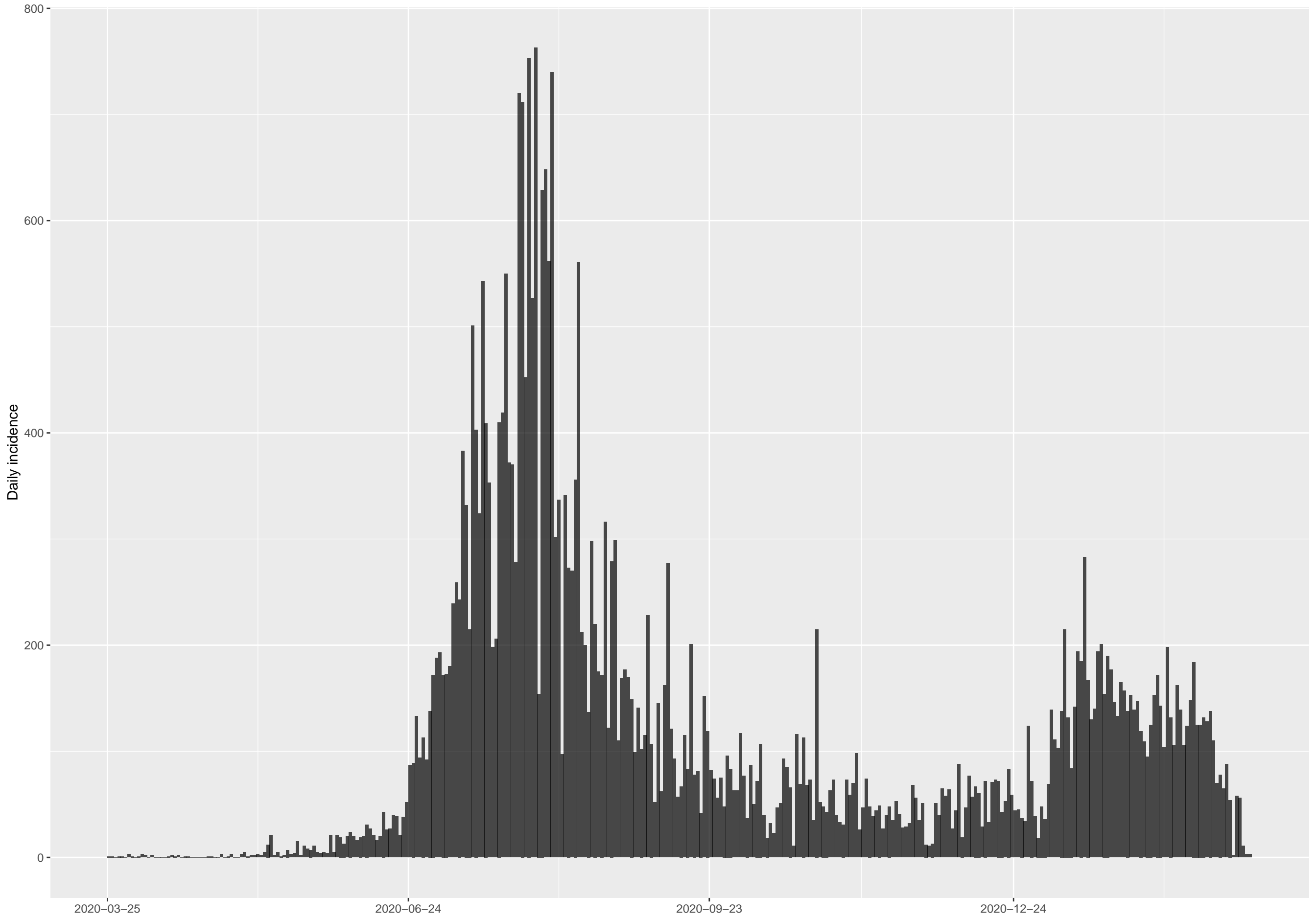

Supplement: Supplementary file 1 [file vaccines-09-00837-s001.zip › Supplementary_material/Supplementary Data S3/incidence_plot/WHO_model_incidence_ CO .pdf]

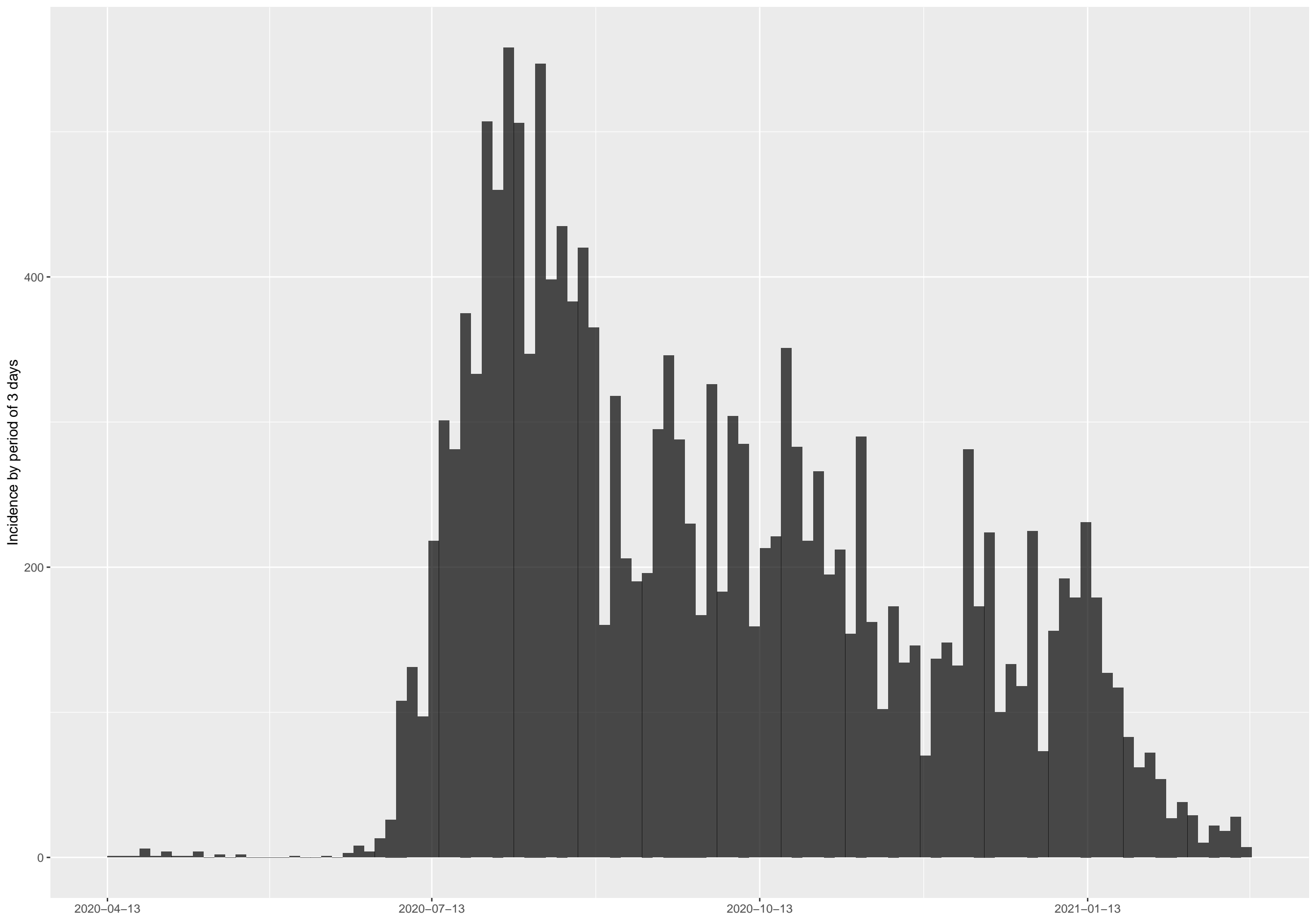

Supplement: Supplementary file 1 [file vaccines-09-00837-s001.zip › Supplementary_material/Supplementary Data S3/incidence_plot/WHO_model_incidence_ CQ .pdf]

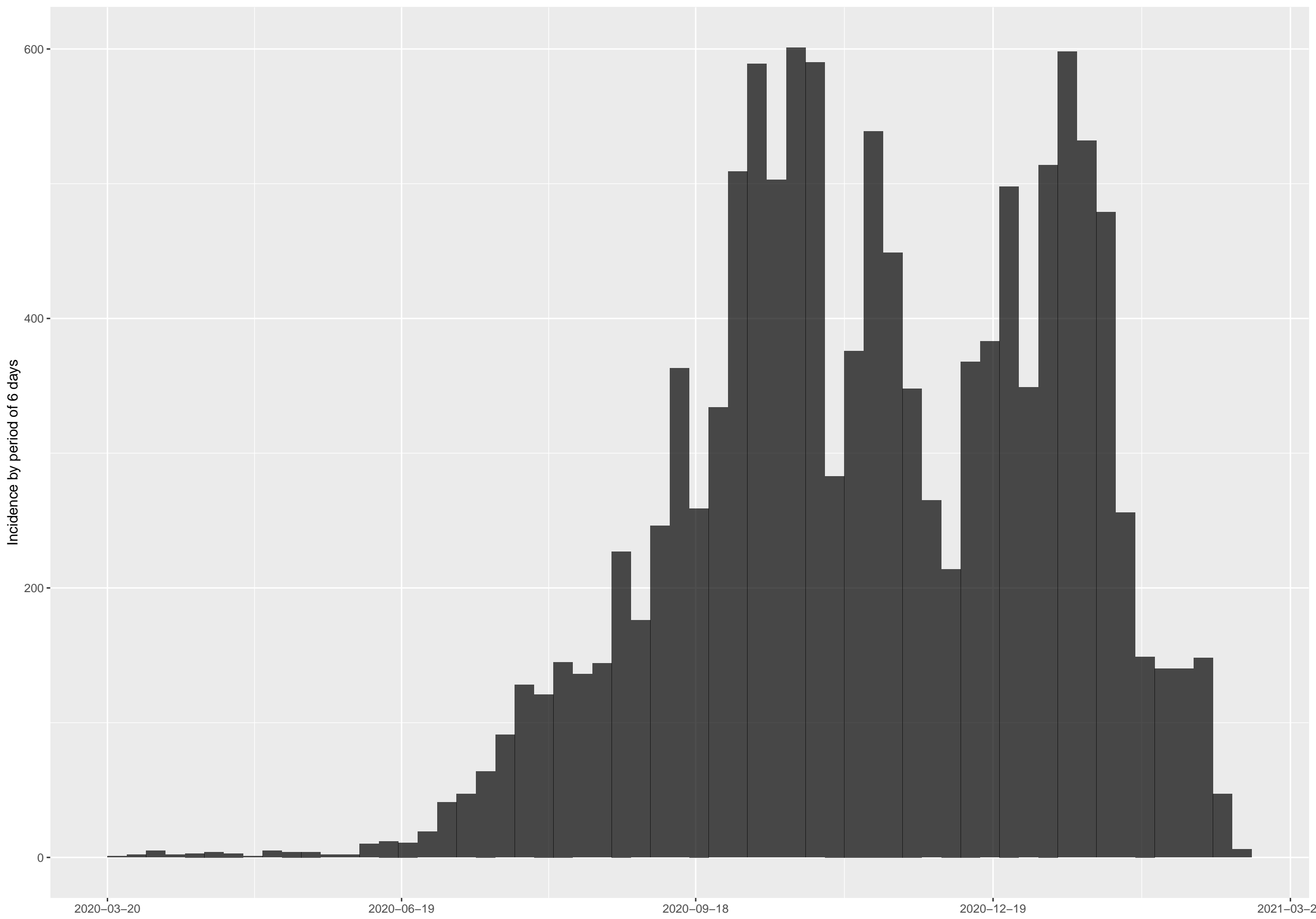

Supplement: Supplementary file 1 [file vaccines-09-00837-s001.zip › Supplementary_material/Supplementary Data S3/incidence_plot/WHO_model_incidence_ CS .pdf]

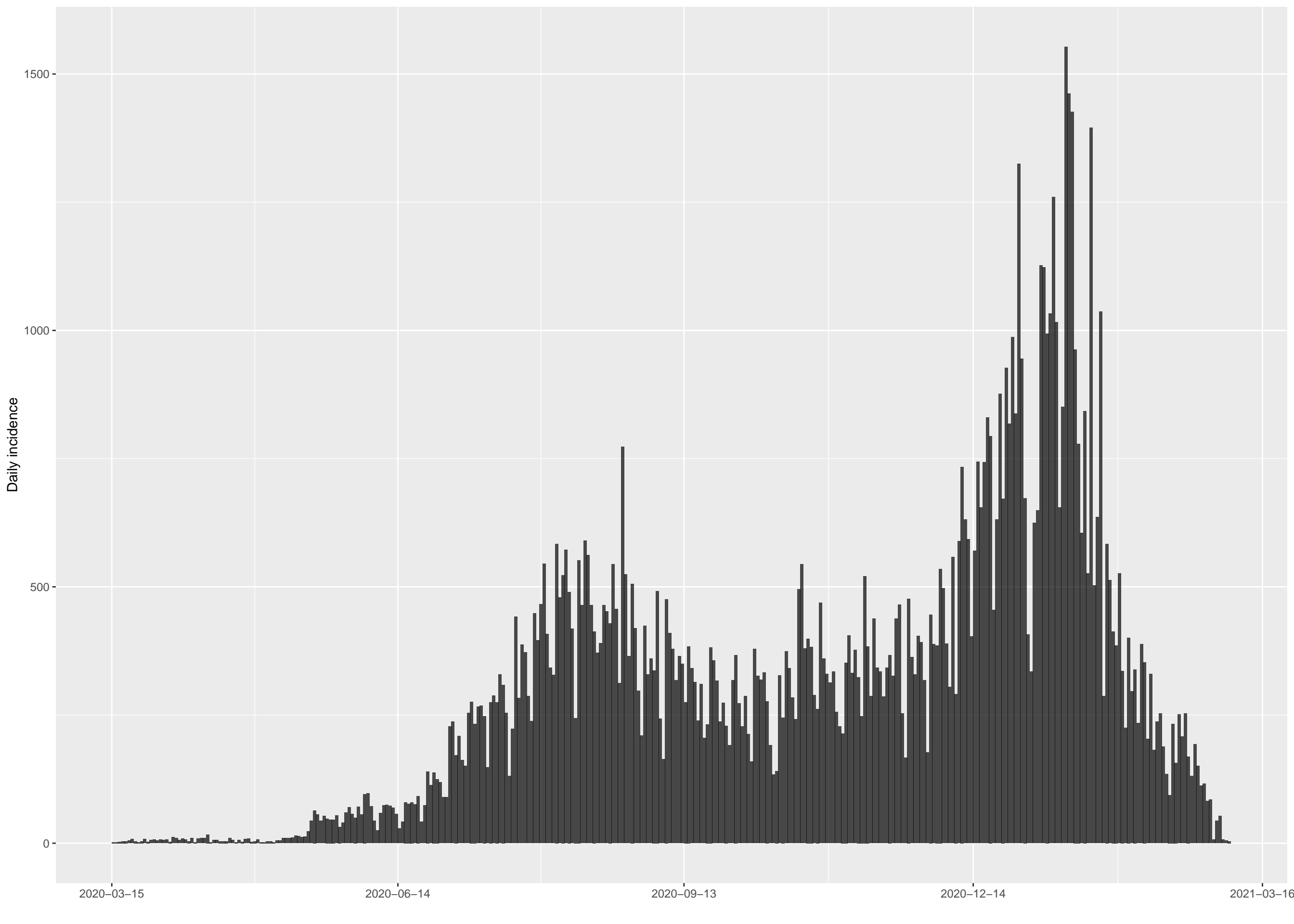

Supplement: Supplementary file 1 [file vaccines-09-00837-s001.zip › Supplementary_material/Supplementary Data S3/incidence_plot/WHO_model_incidence_ CU .pdf]

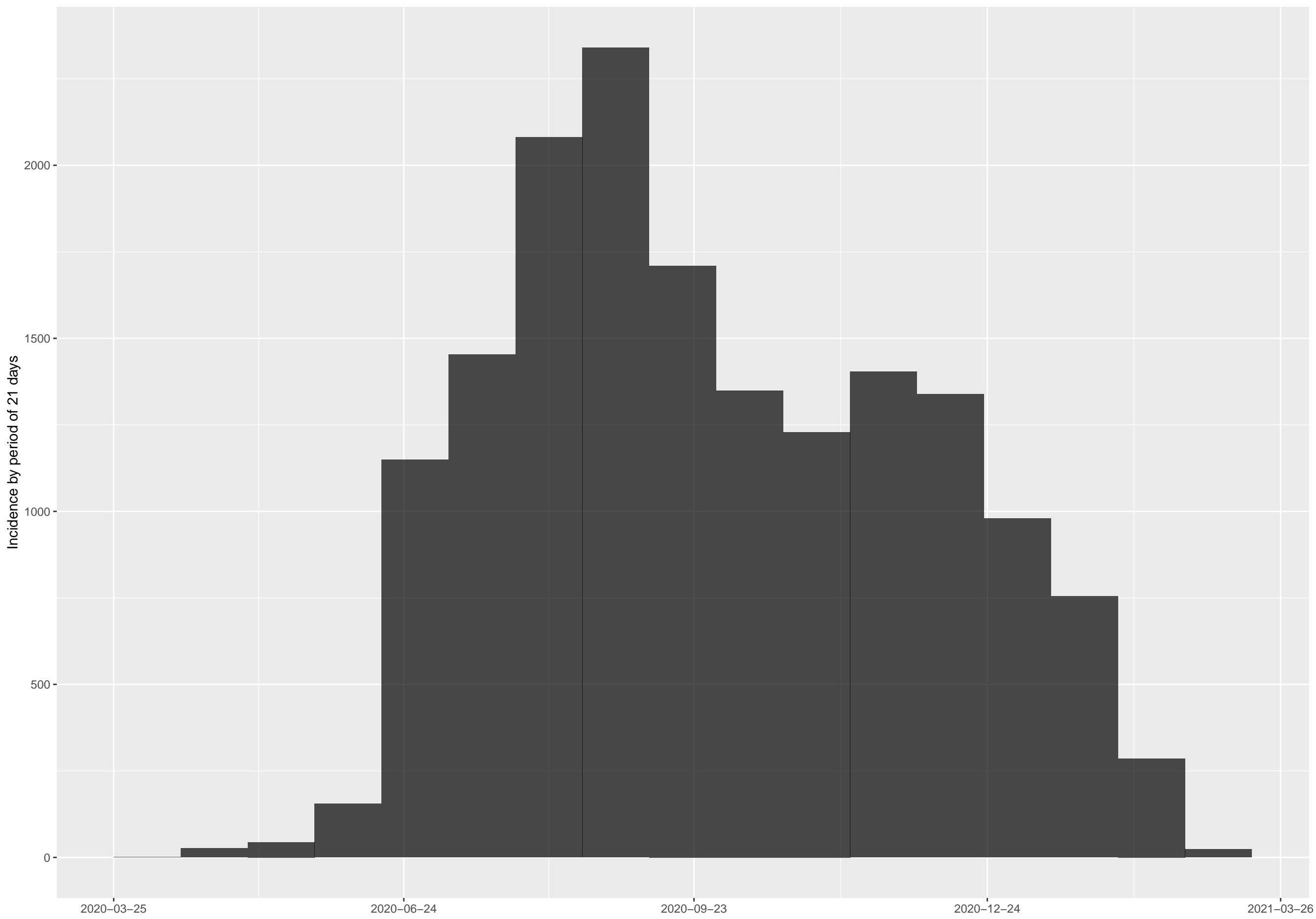

Supplement: Supplementary file 1 [file vaccines-09-00837-s001.zip › Supplementary_material/Supplementary Data S3/incidence_plot/WHO_model_incidence_ GJ .pdf]

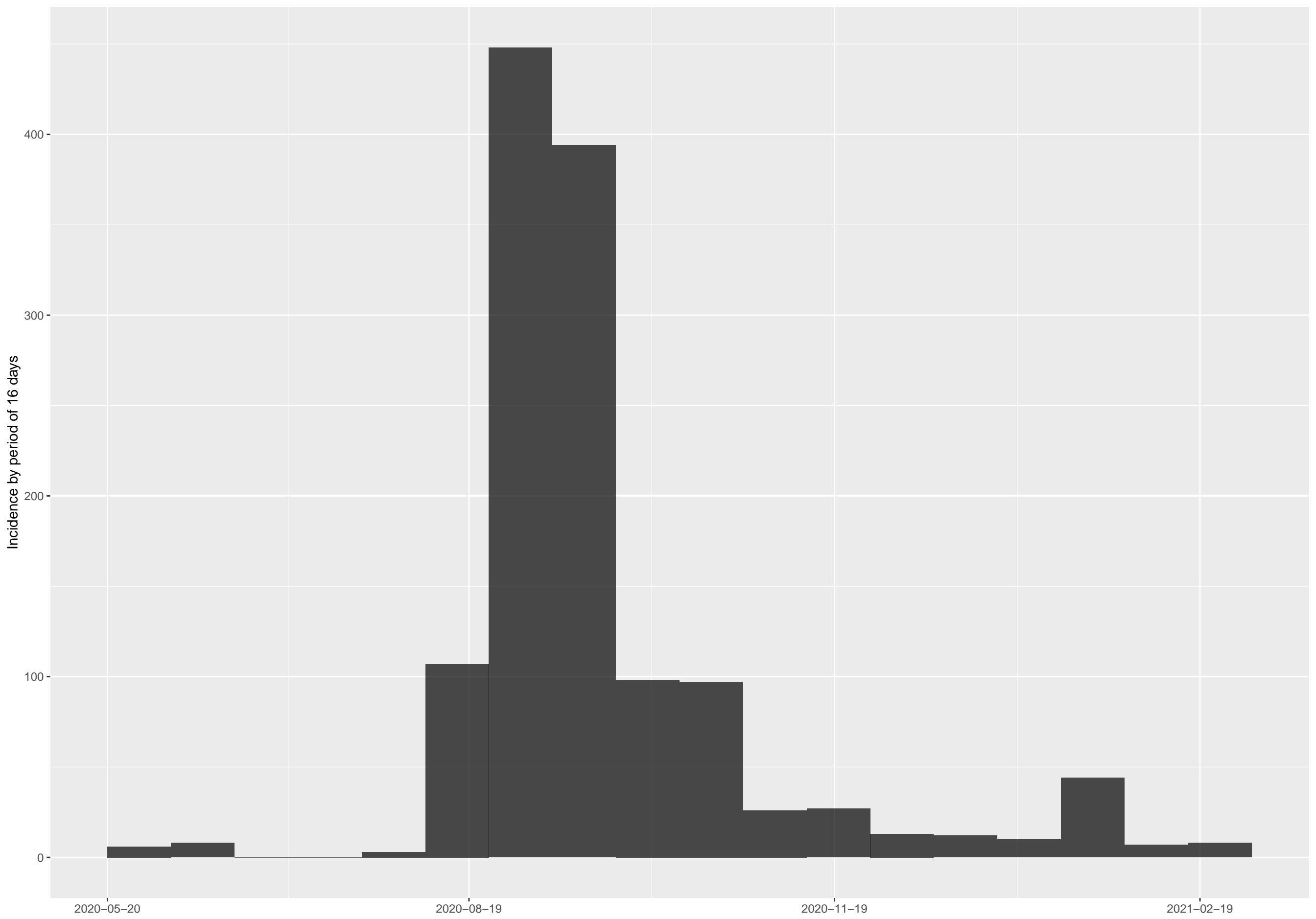

Supplement: Supplementary file 1 [file vaccines-09-00837-s001.zip › Supplementary_material/Supplementary Data S3/incidence_plot/WHO_model_incidence_ GN .pdf]

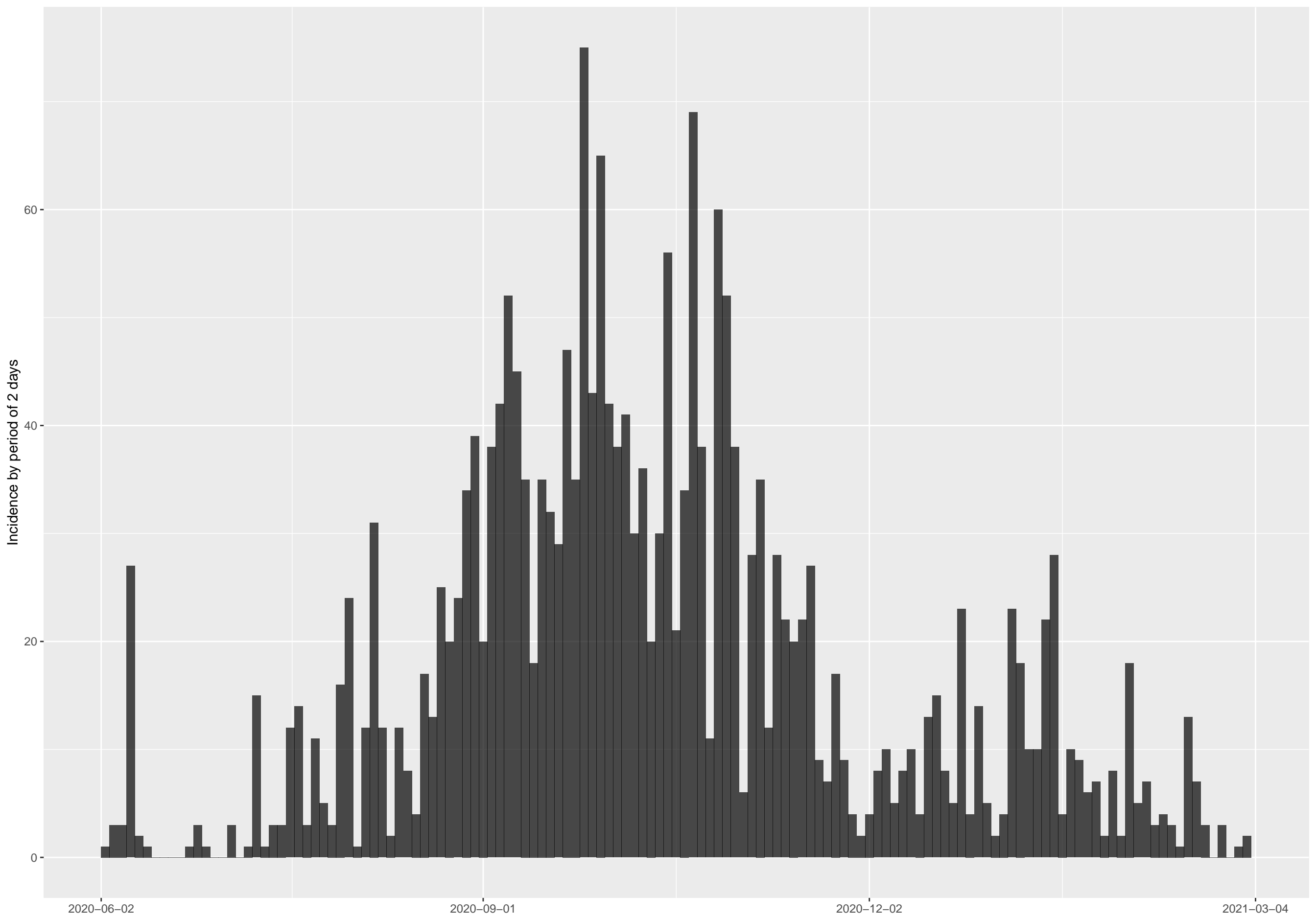

Supplement: Supplementary file 1 [file vaccines-09-00837-s001.zip › Supplementary_material/Supplementary Data S3/incidence_plot/WHO_model_incidence_ GV .pdf]

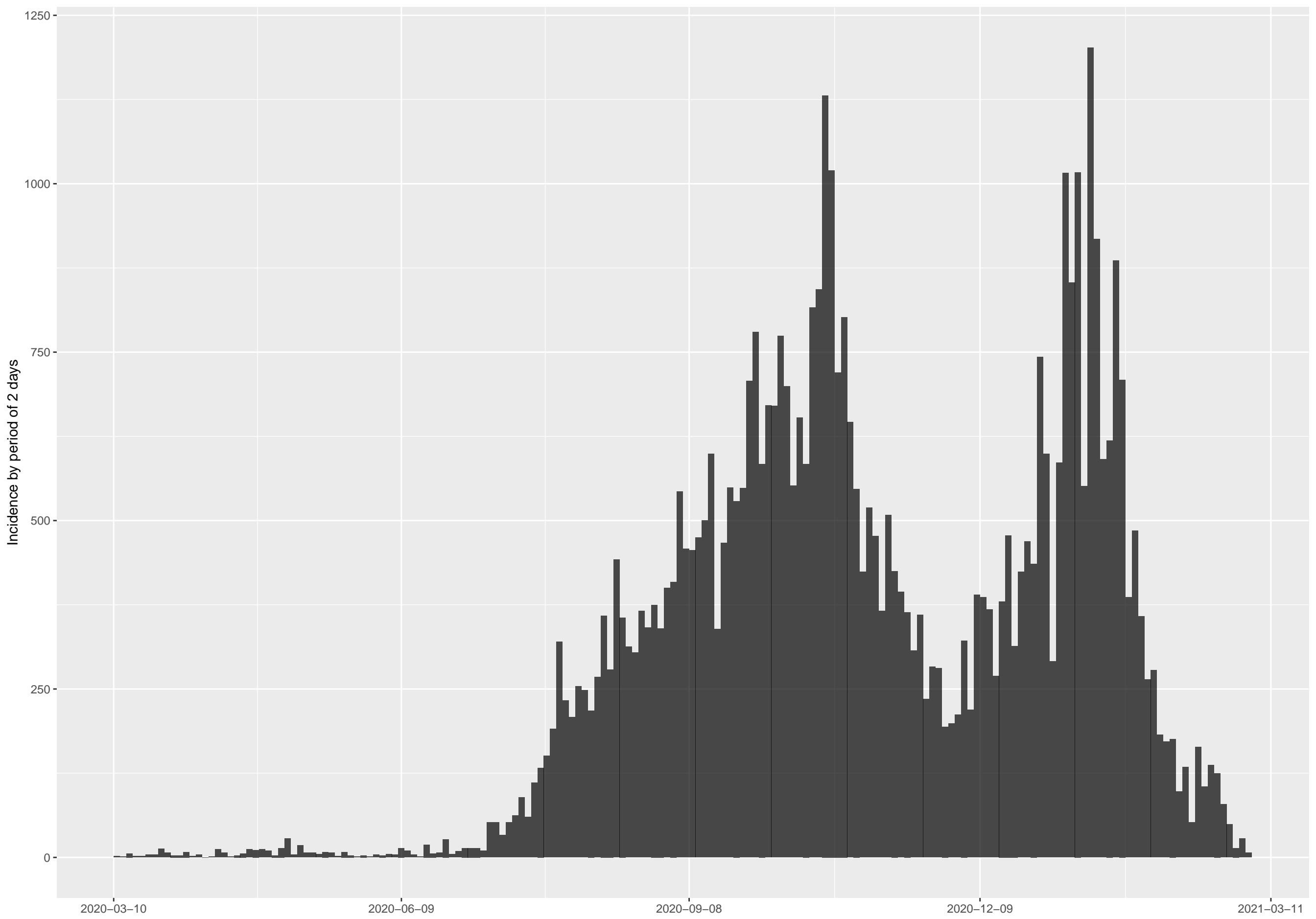

Supplement: Supplementary file 1 [file vaccines-09-00837-s001.zip › Supplementary_material/Supplementary Data S3/incidence_plot/WHO_model_incidence_ HU .pdf]

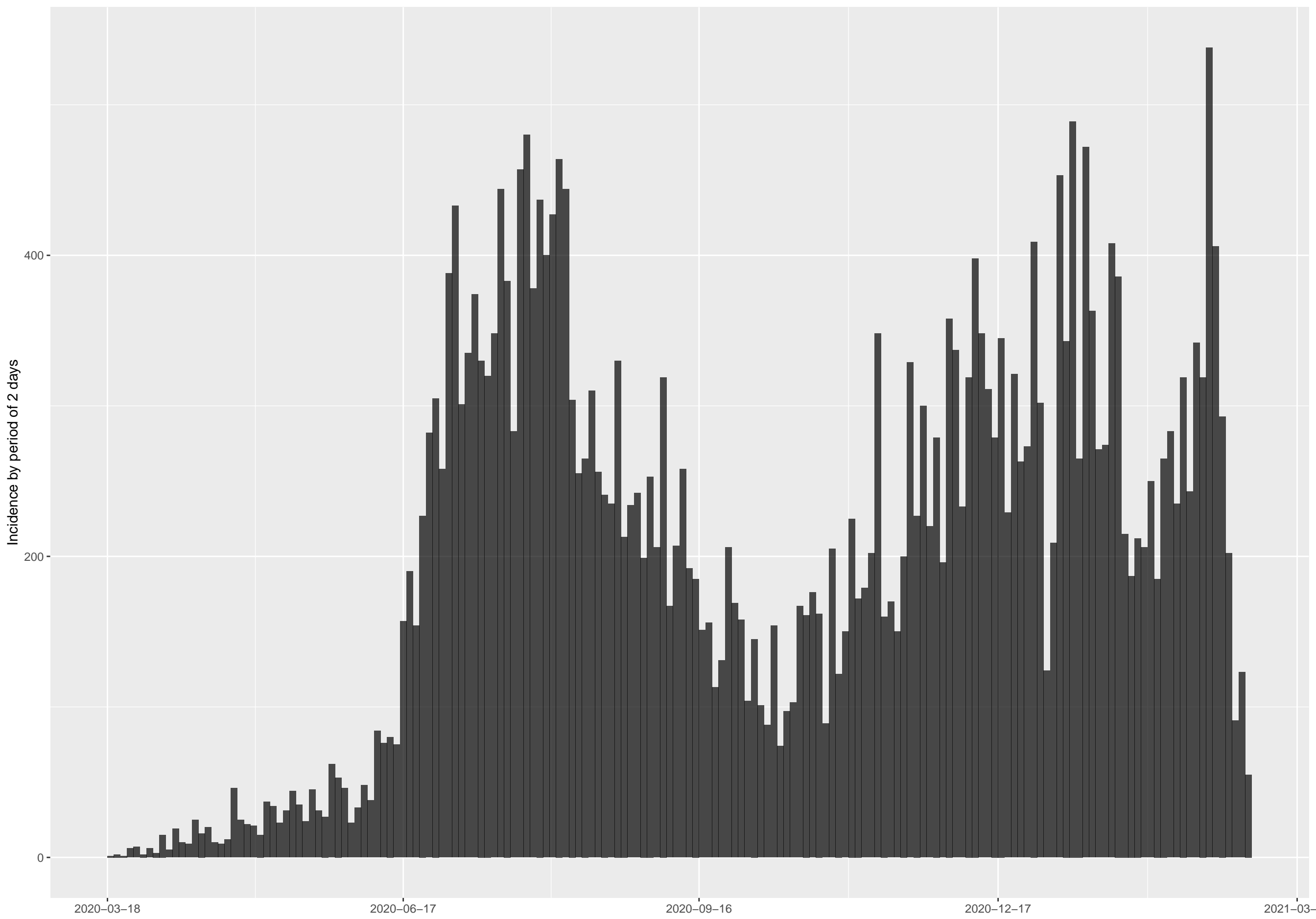

Supplement: Supplementary file 1 [file vaccines-09-00837-s001.zip › Supplementary_material/Supplementary Data S3/incidence_plot/WHO_model_incidence_ MA .pdf]

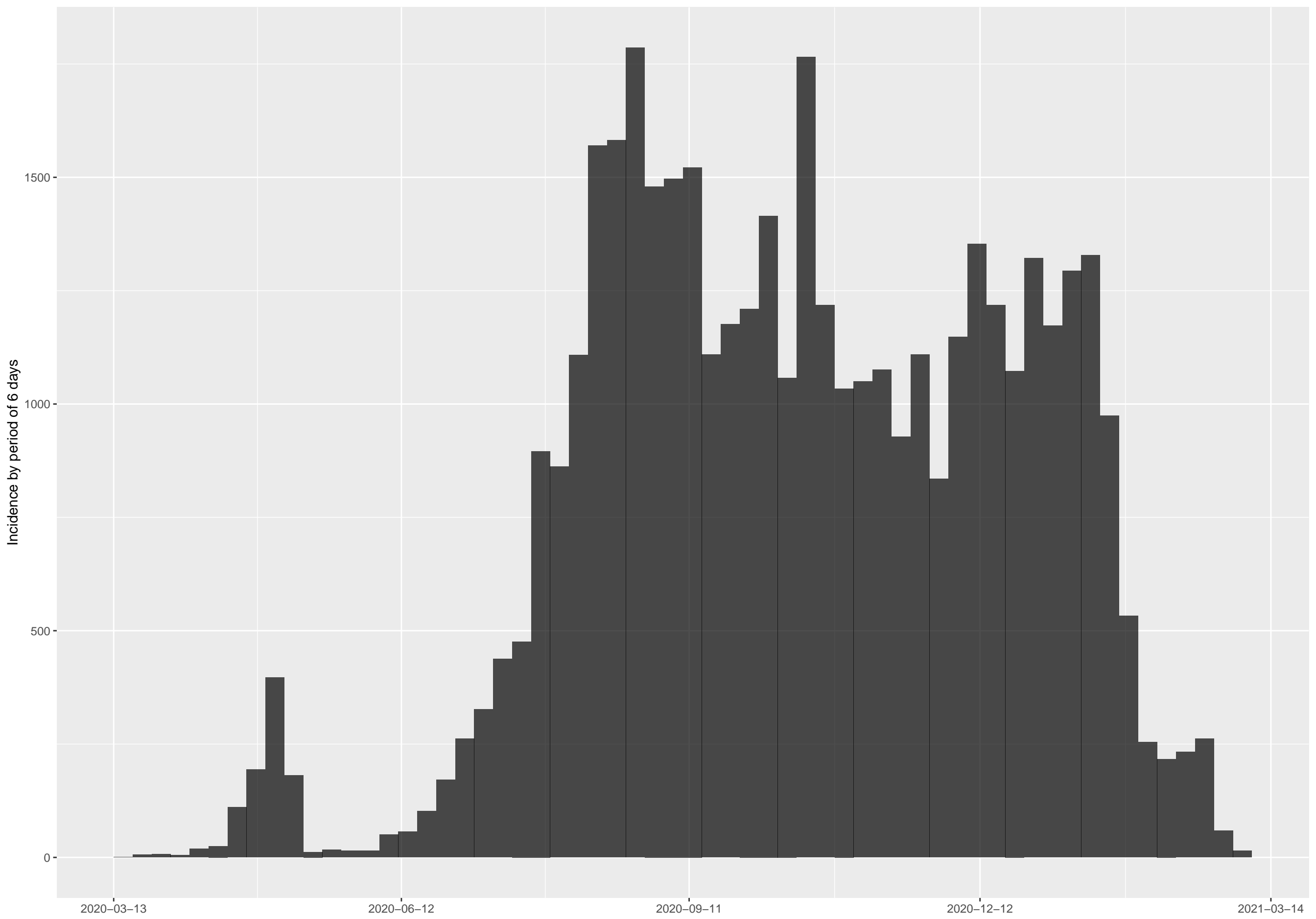

Supplement: Supplementary file 1 [file vaccines-09-00837-s001.zip › Supplementary_material/Supplementary Data S3/incidence_plot/WHO_model_incidence_ ME .pdf]

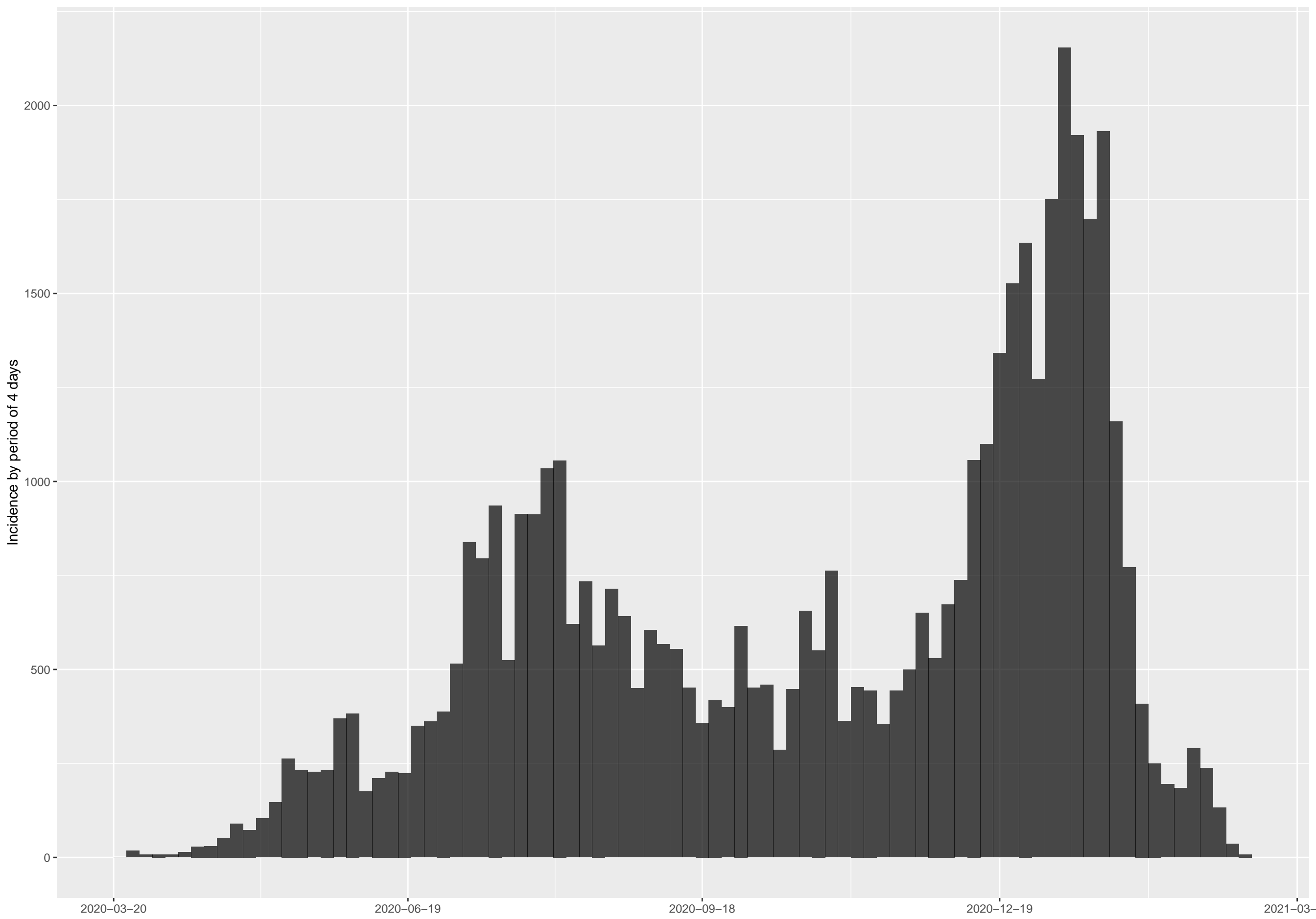

Supplement: Supplementary file 1 [file vaccines-09-00837-s001.zip › Supplementary_material/Supplementary Data S3/incidence_plot/WHO_model_incidence_ NA .pdf]

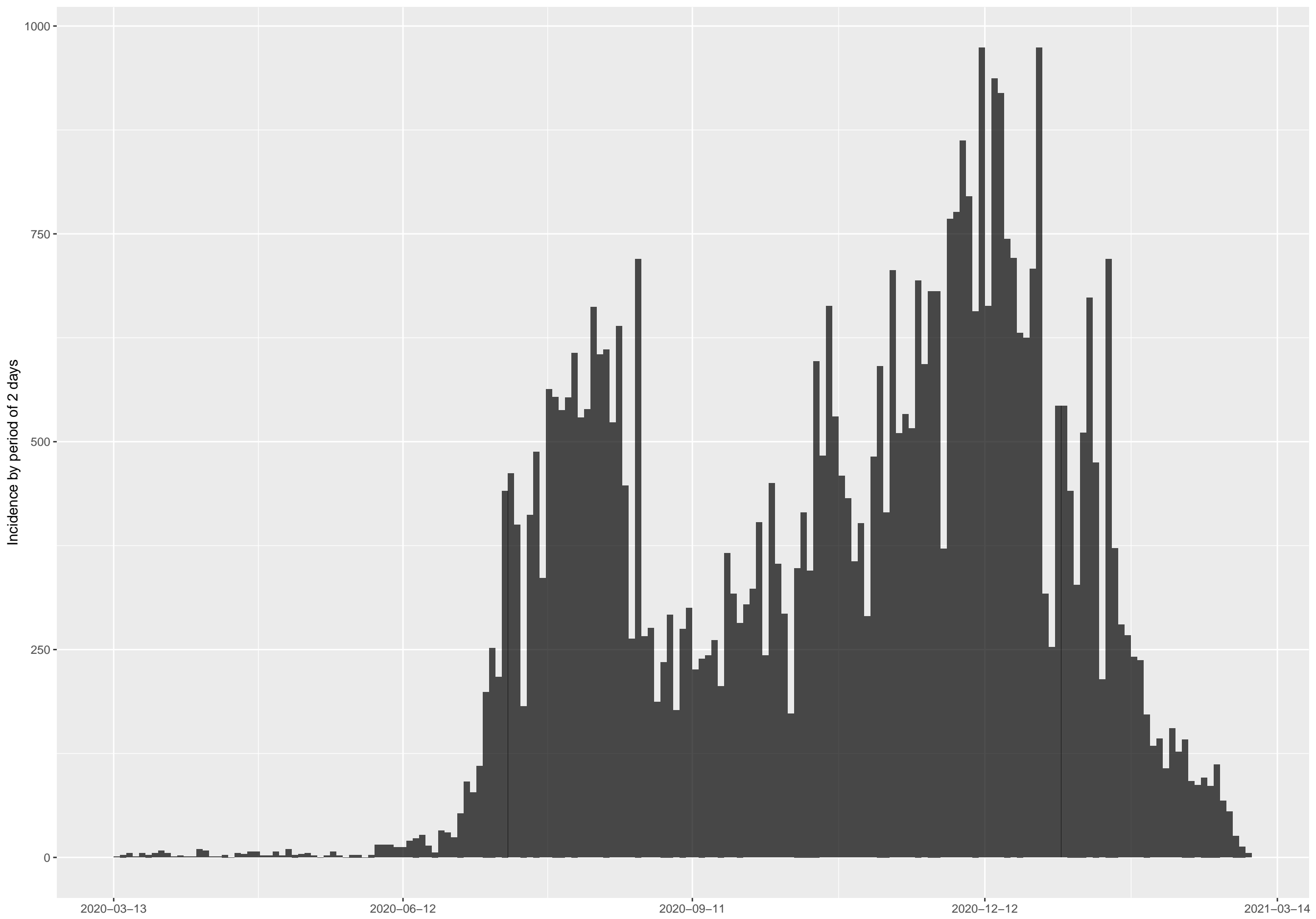

Supplement: Supplementary file 1 [file vaccines-09-00837-s001.zip › Supplementary_material/Supplementary Data S3/incidence_plot/WHO_model_incidence_ NS .pdf]

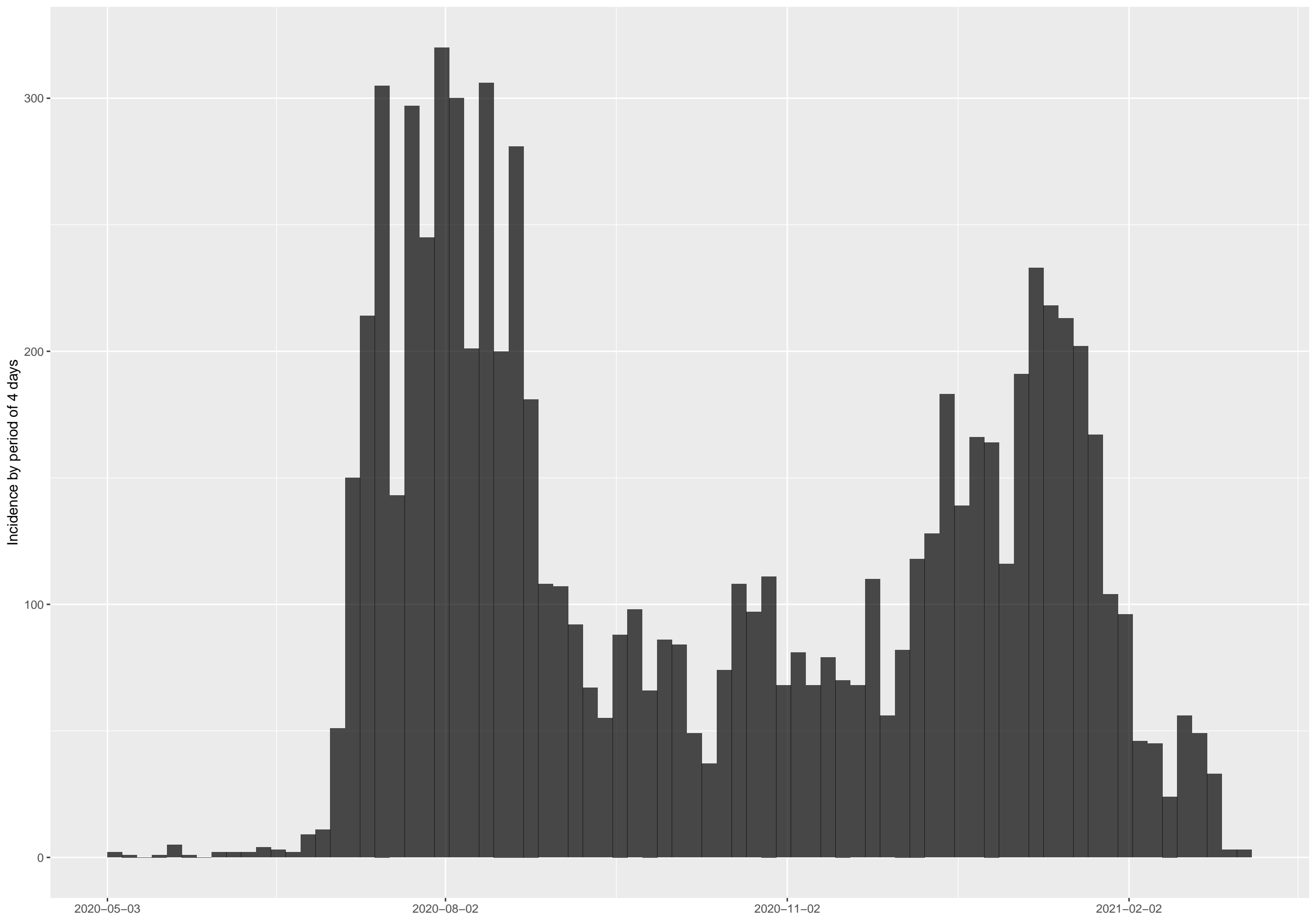

Supplement: Supplementary file 1 [file vaccines-09-00837-s001.zip › Supplementary_material/Supplementary Data S3/incidence_plot/WHO_model_incidence_ PU .pdf]

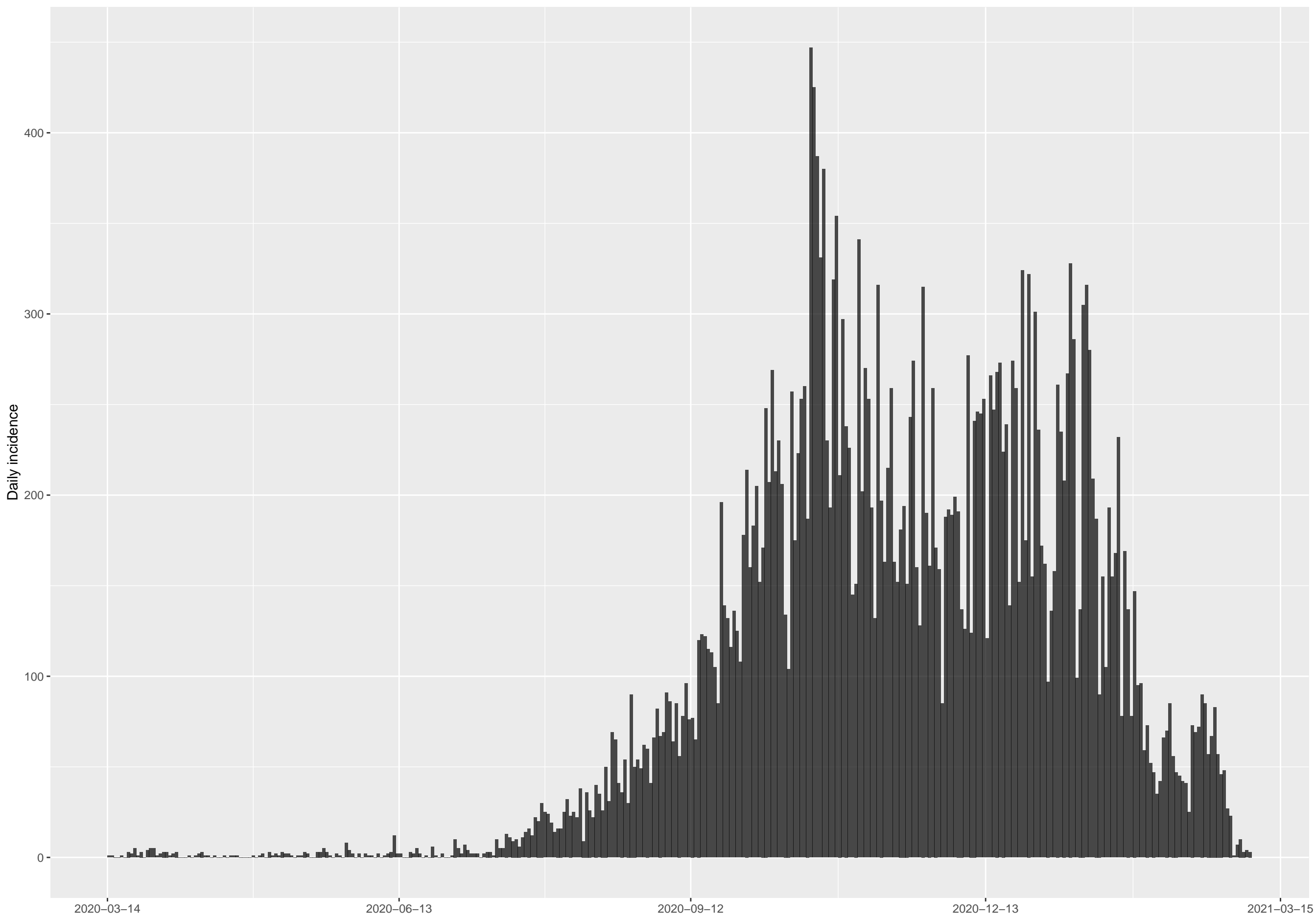

Supplement: Supplementary file 1 [file vaccines-09-00837-s001.zip › Supplementary_material/Supplementary Data S3/incidence_plot/WHO_model_incidence_ QU .pdf]

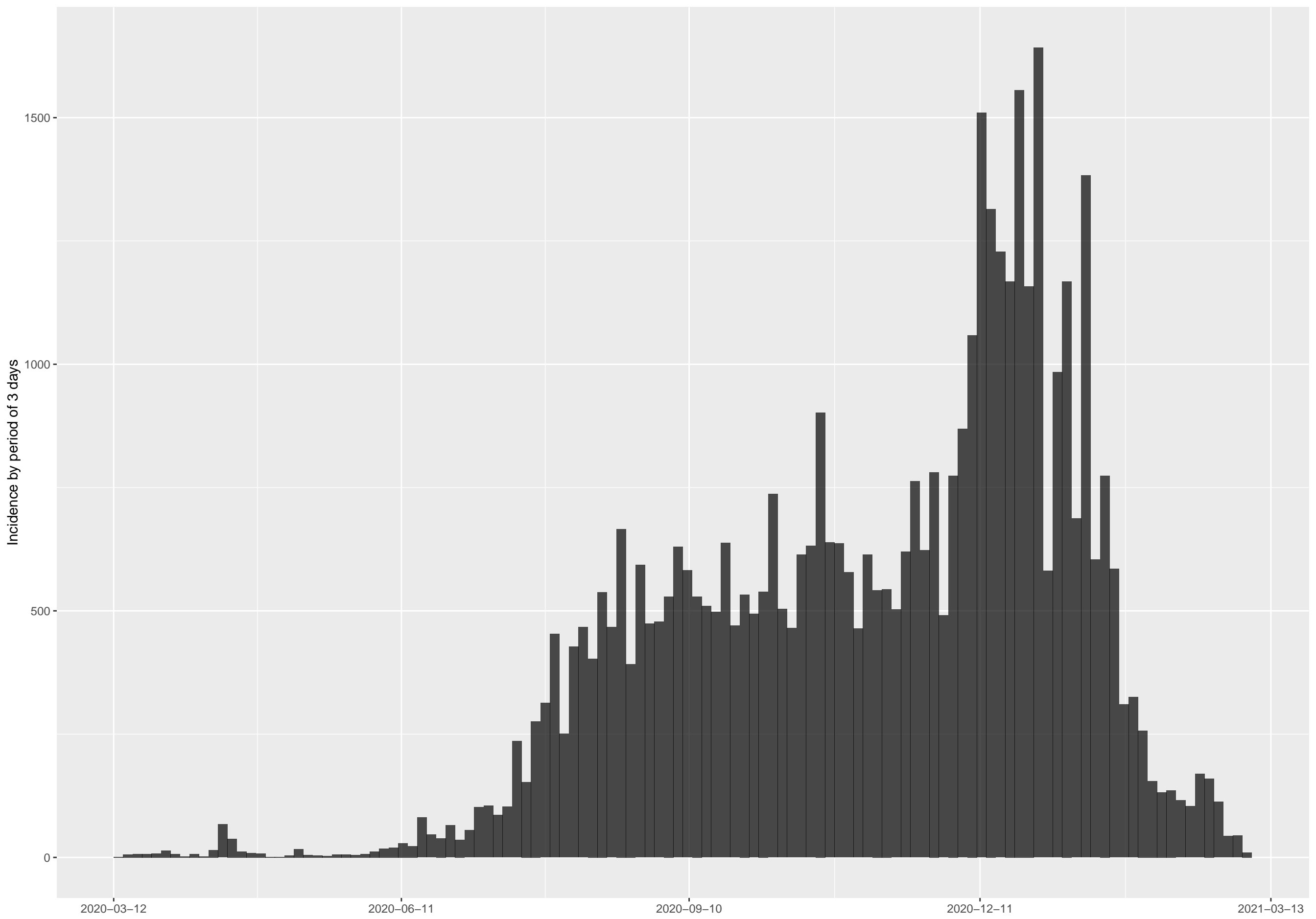

Supplement: Supplementary file 1 [file vaccines-09-00837-s001.zip › Supplementary_material/Supplementary Data S3/incidence_plot/WHO_model_incidence_ RI .pdf]

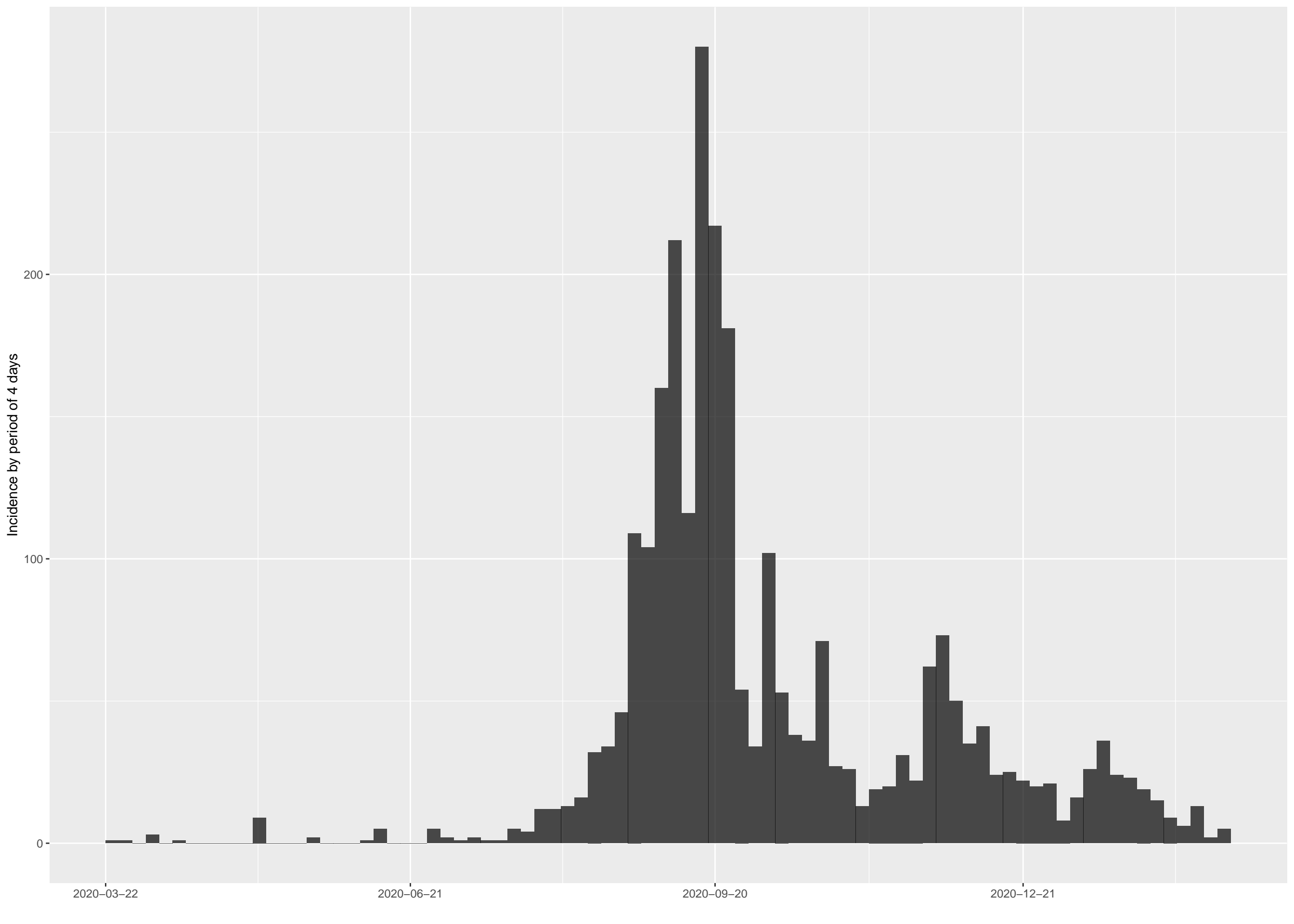

Supplement: Supplementary file 1 [file vaccines-09-00837-s001.zip › Supplementary_material/Supplementary Data S3/incidence_plot/WHO_model_incidence_ SA .pdf]

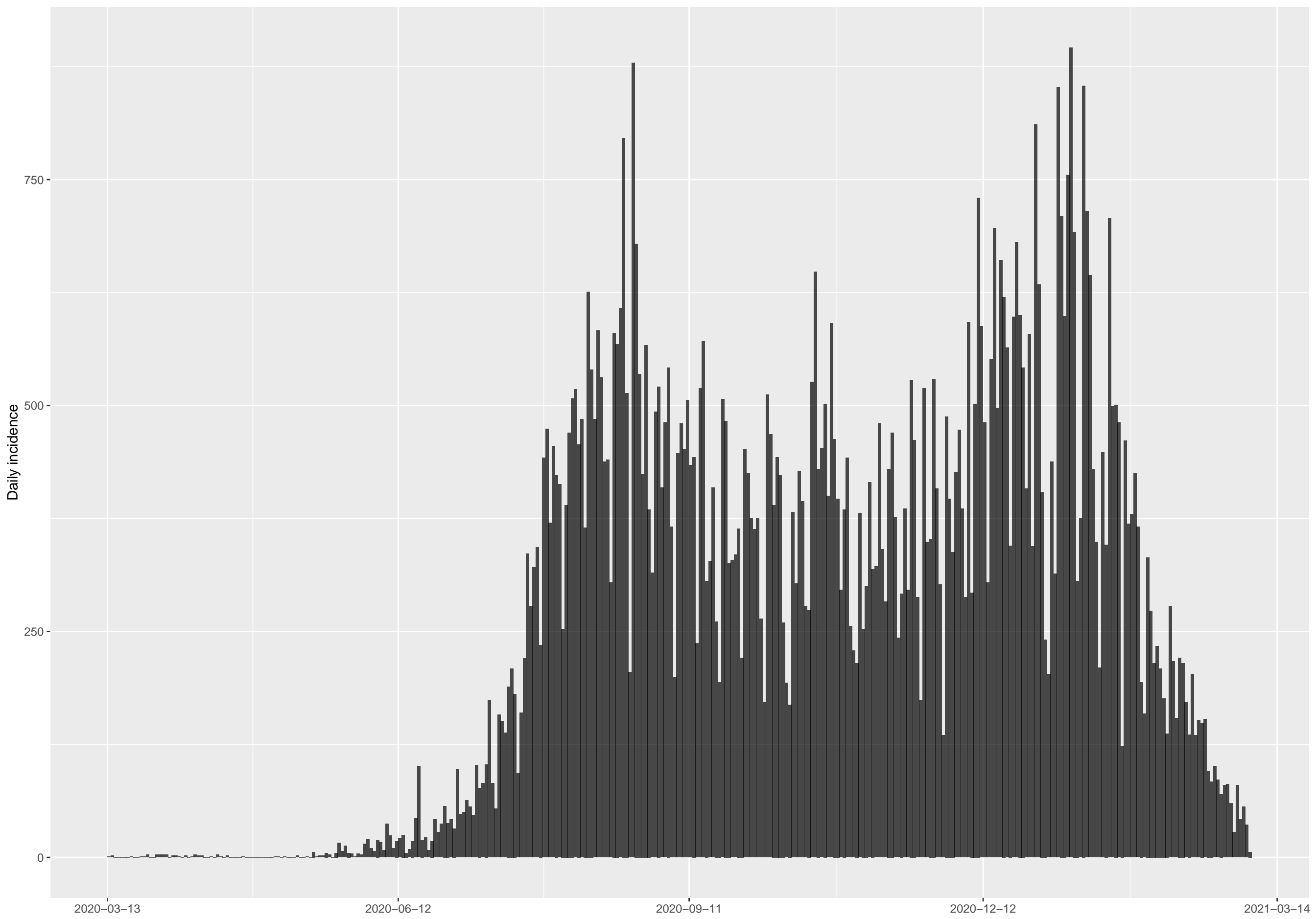

Supplement: Supplementary file 1 [file vaccines-09-00837-s001.zip › Supplementary_material/Supplementary Data S3/incidence_plot/WHO_model_incidence_ SN .pdf]

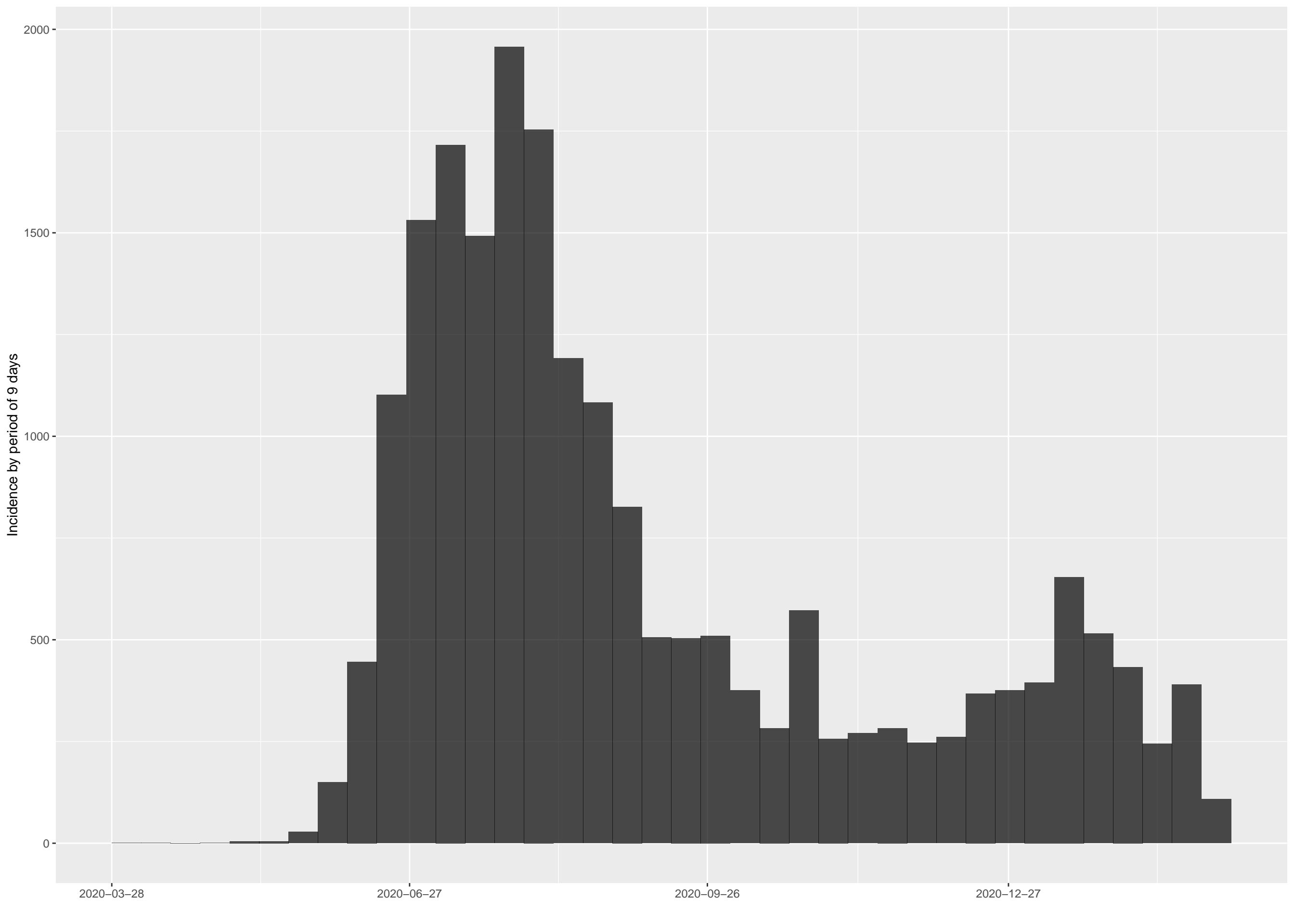

Supplement: Supplementary file 1 [file vaccines-09-00837-s001.zip › Supplementary_material/Supplementary Data S3/incidence_plot/WHO_model_incidence_ SU .pdf]

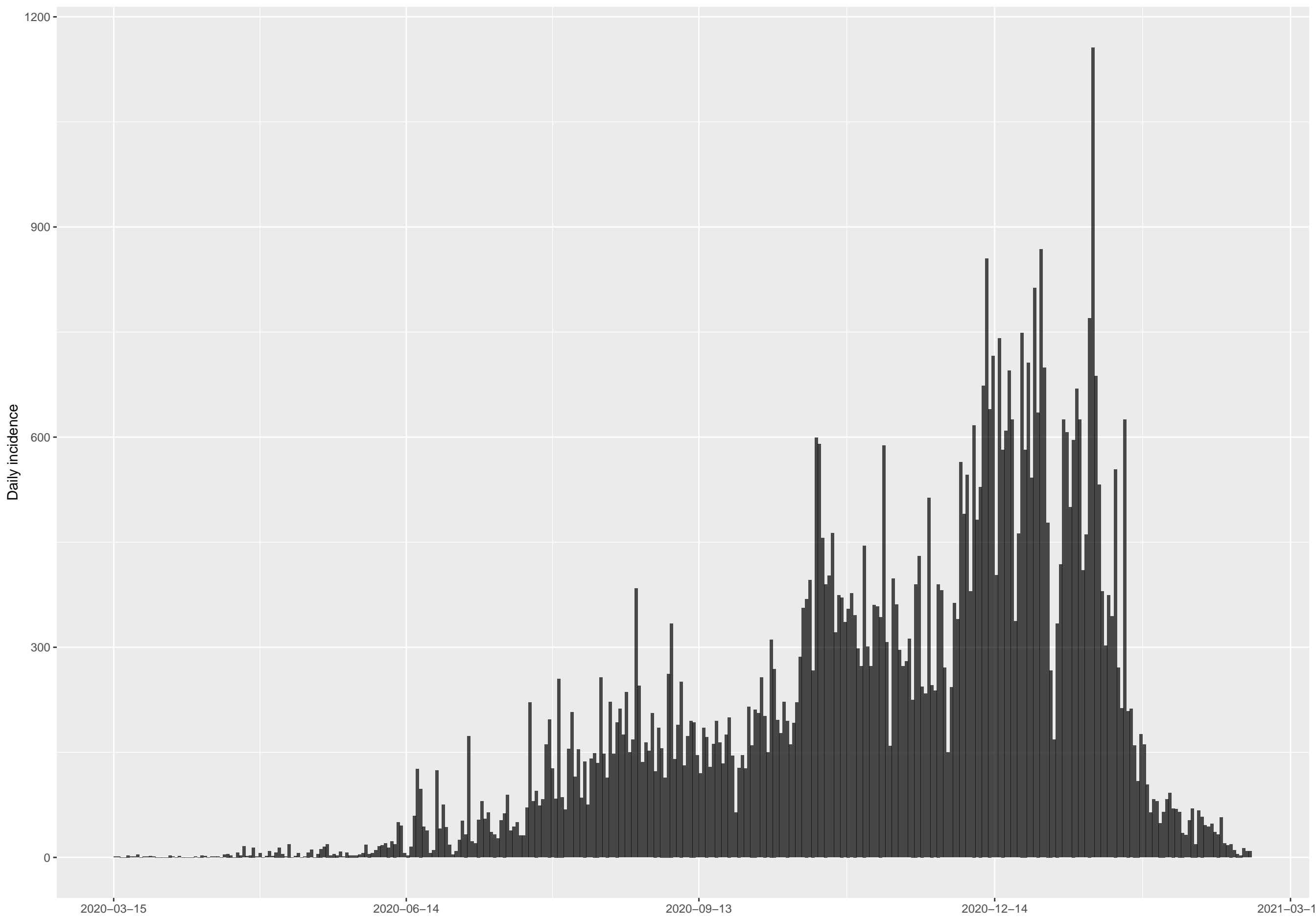

Supplement: Supplementary file 1 [file vaccines-09-00837-s001.zip › Supplementary_material/Supplementary Data S3/incidence_plot/WHO_model_incidence_ TO .pdf]

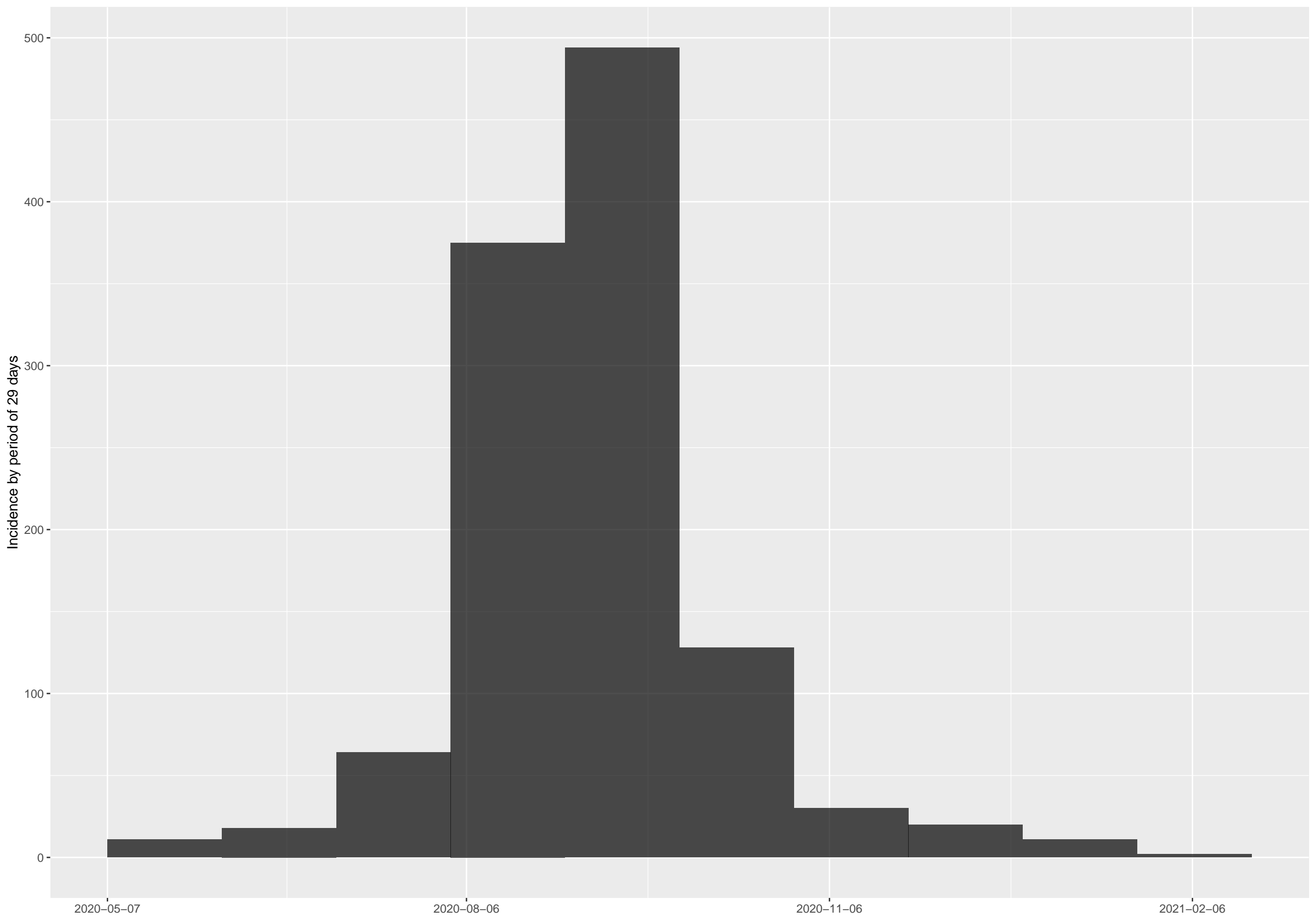

Supplement: Supplementary file 1 [file vaccines-09-00837-s001.zip › Supplementary_material/Supplementary Data S3/incidence_plot/WHO_model_incidence_ VA .pdf]

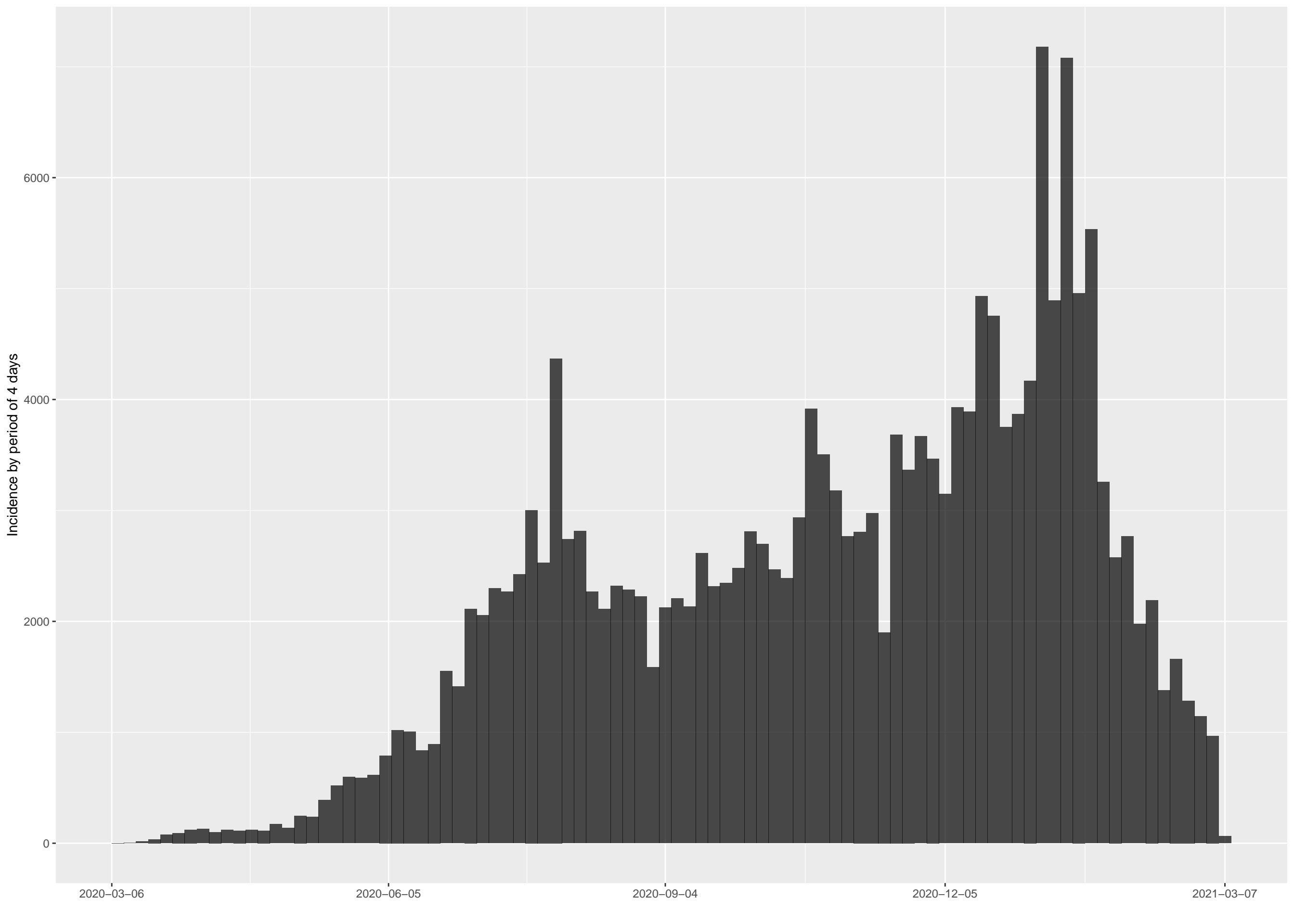

Supplement: Supplementary file 1 [file vaccines-09-00837-s001.zip › Supplementary_material/Supplementary Data S3/incidence_plot/WHO_model_incidence_ VC .pdf]

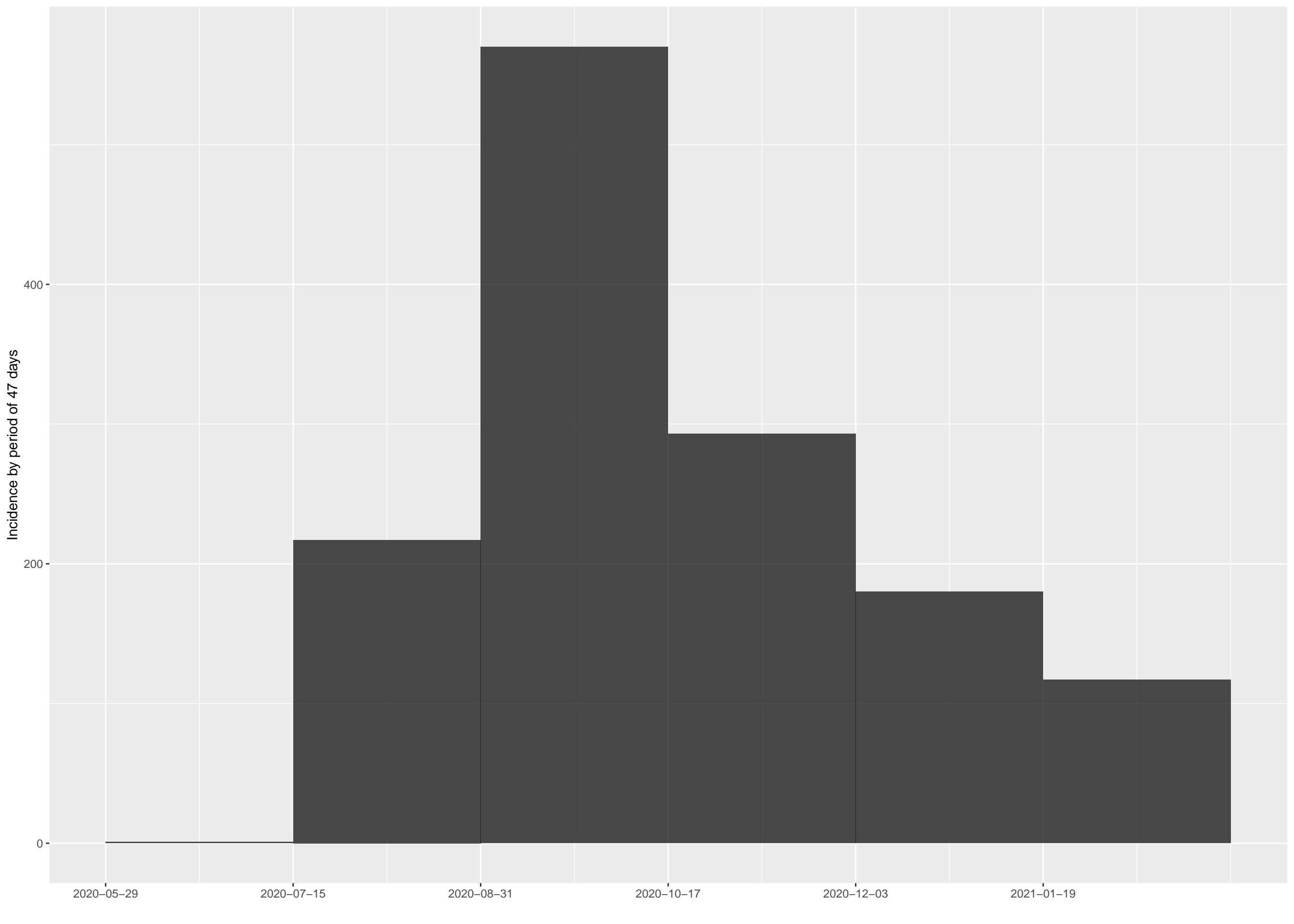

Supplement: Supplementary file 1 [file vaccines-09-00837-s001.zip › Supplementary_material/Supplementary Data S3/incidence_plot/WHO_model_incidence_ VI .pdf]

Estimated R

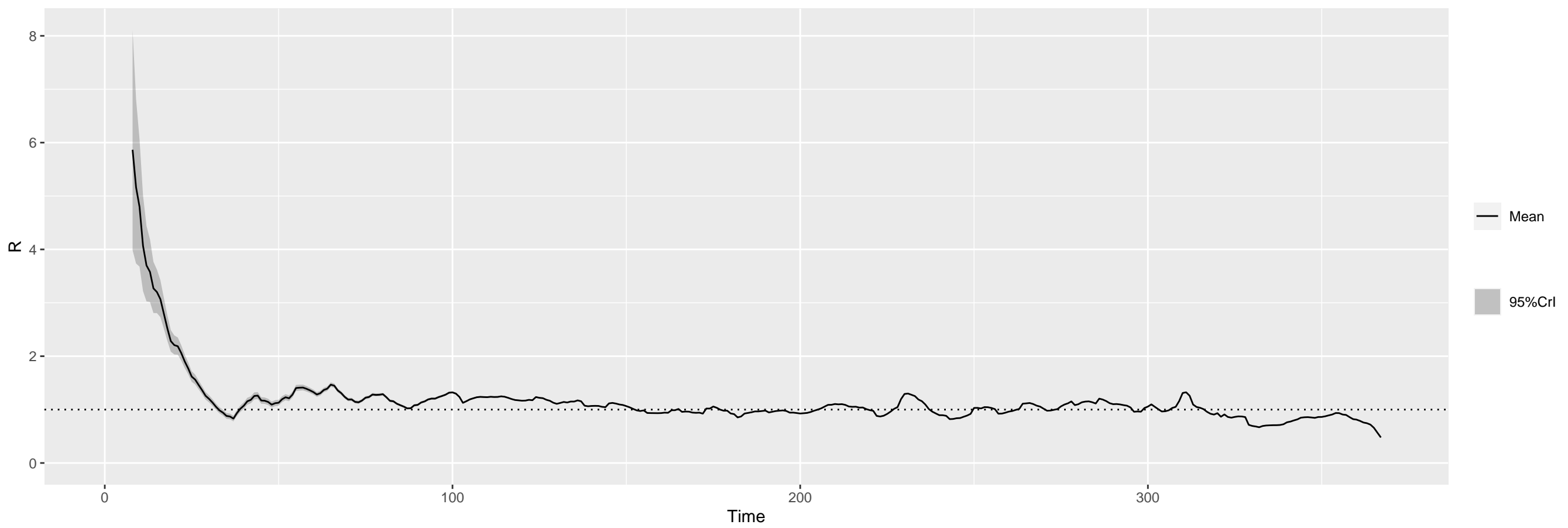

Epidemic curve

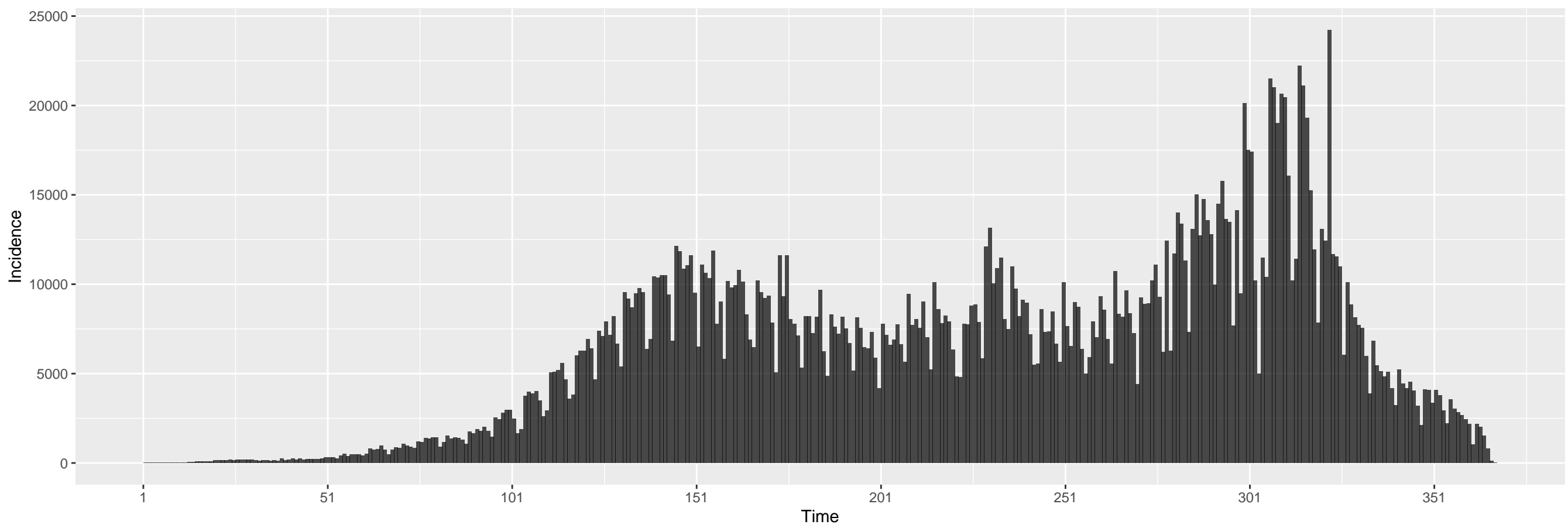

Supplement: Supplementary file 1 [file vaccines-09-00837-s001.zip › Supplementary_material/Supplementary Data S3/summary_plot/who-plot- all .pdf]

Estimated R

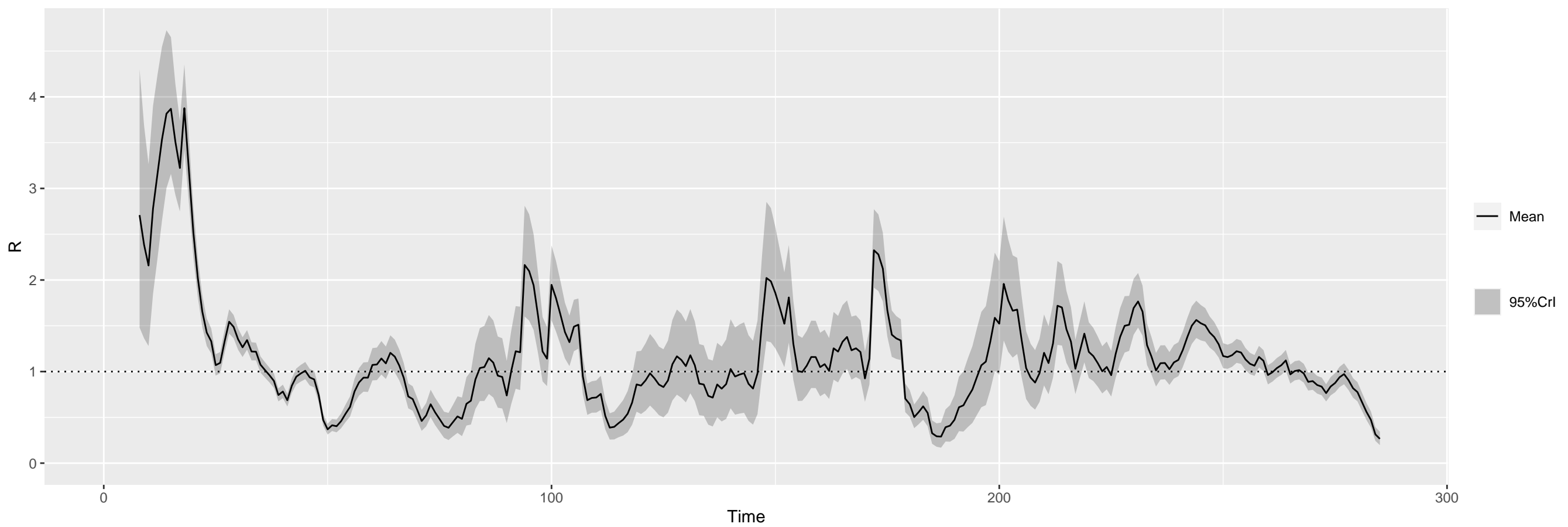

Epidemic curve

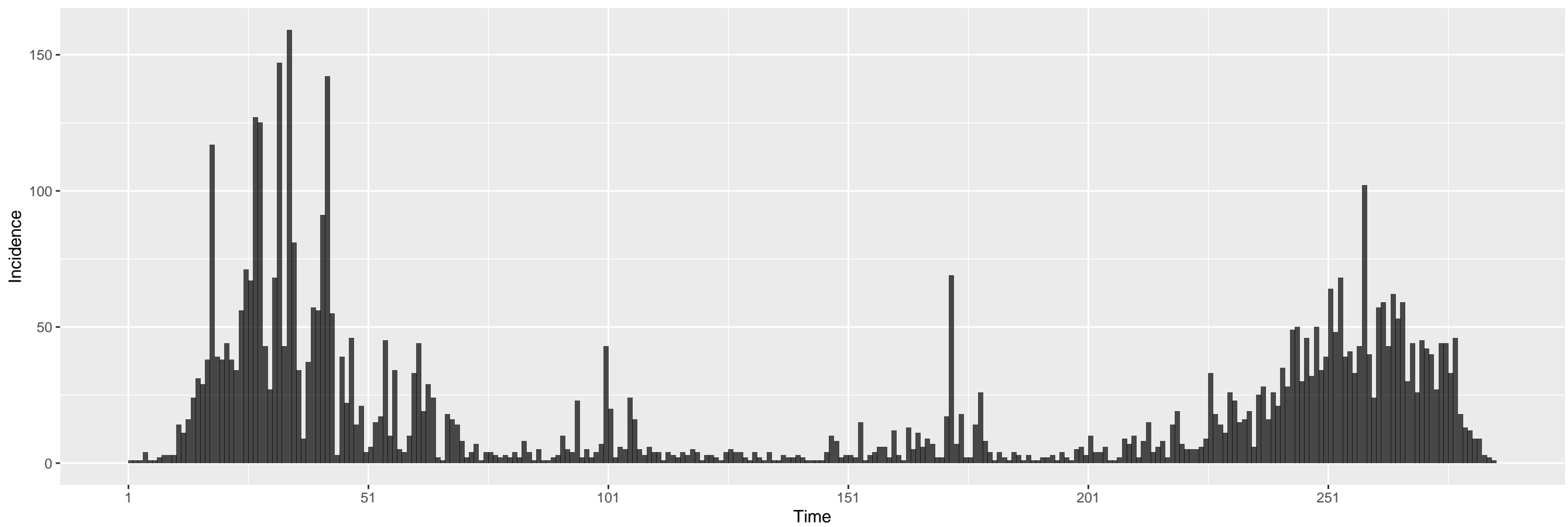

Supplement: Supplementary file 1 [file vaccines-09-00837-s001.zip › Supplementary_material/Supplementary Data S3/summary_plot/who-plot- AM .pdf]

Estimated R

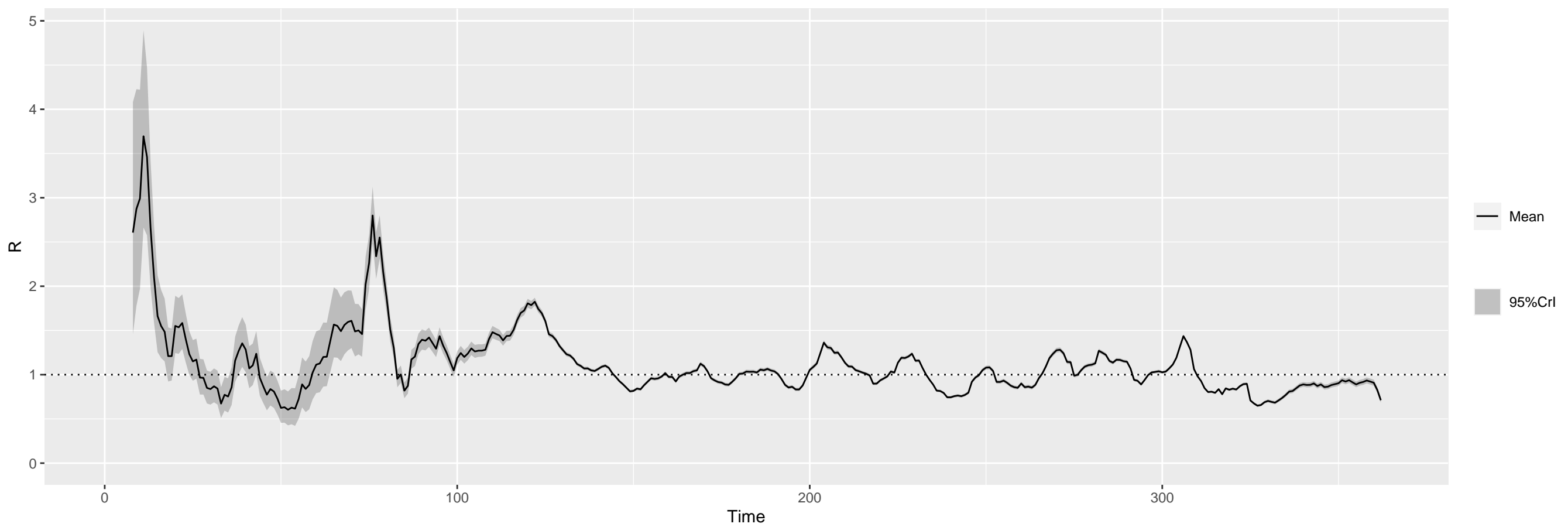

Epidemic curve

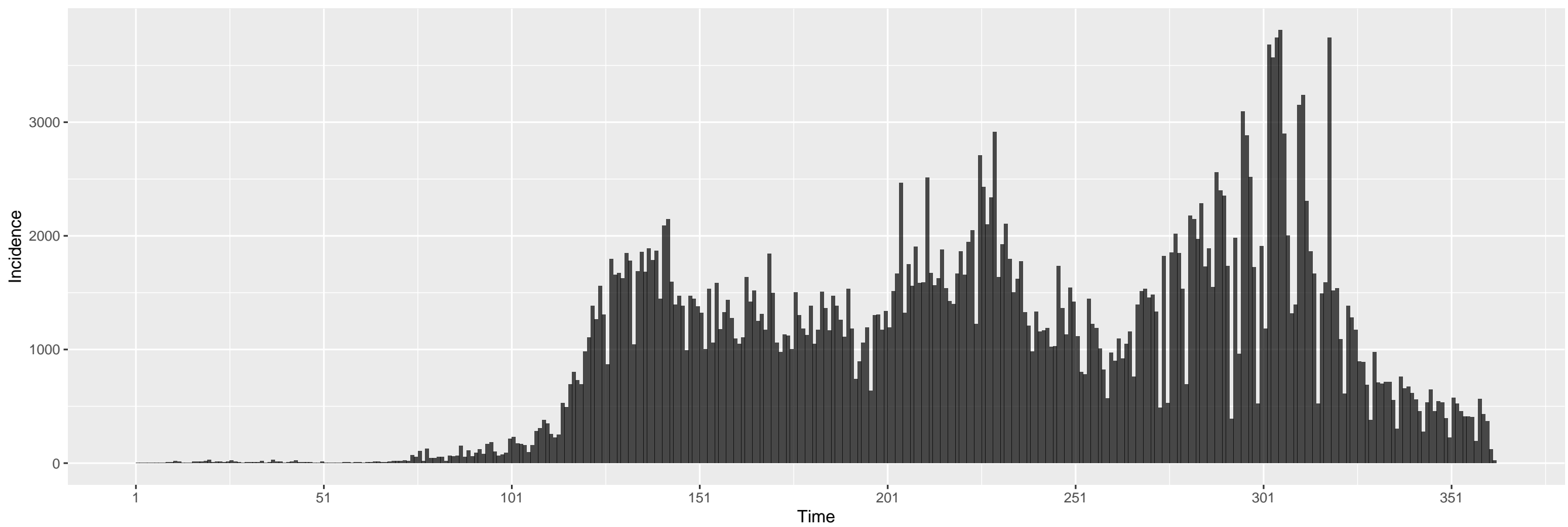

Supplement: Supplementary file 1 [file vaccines-09-00837-s001.zip › Supplementary_material/Supplementary Data S3/summary_plot/who-plot- AN .pdf]

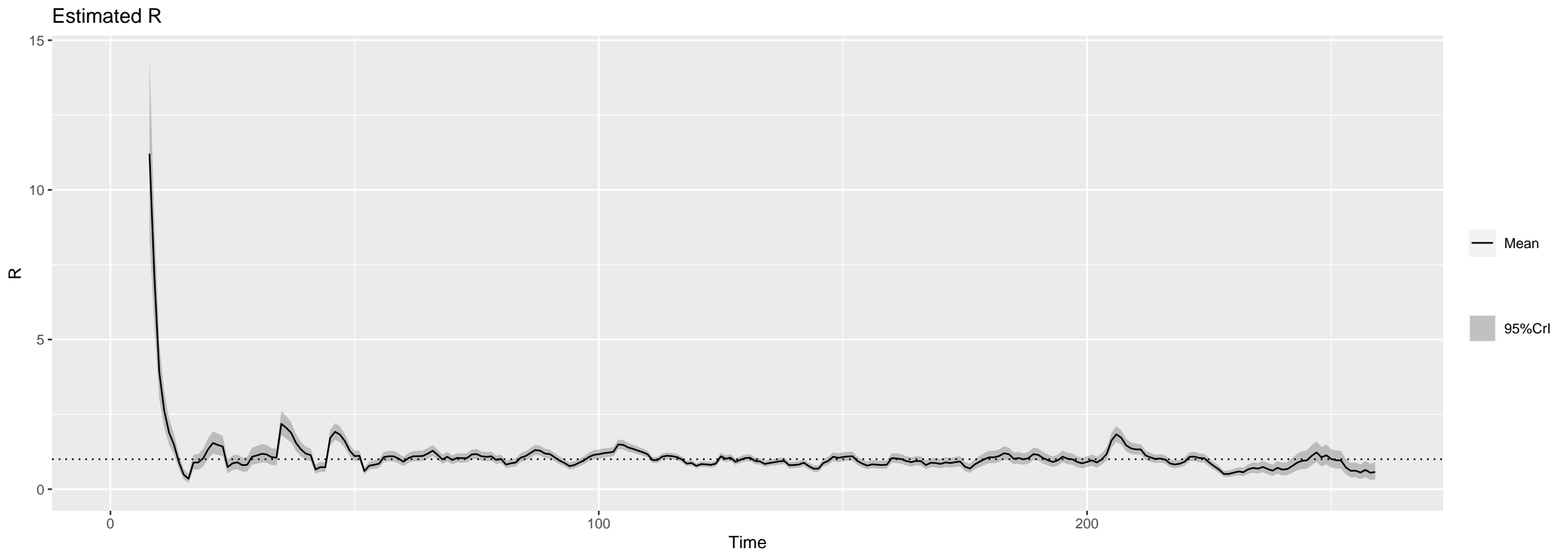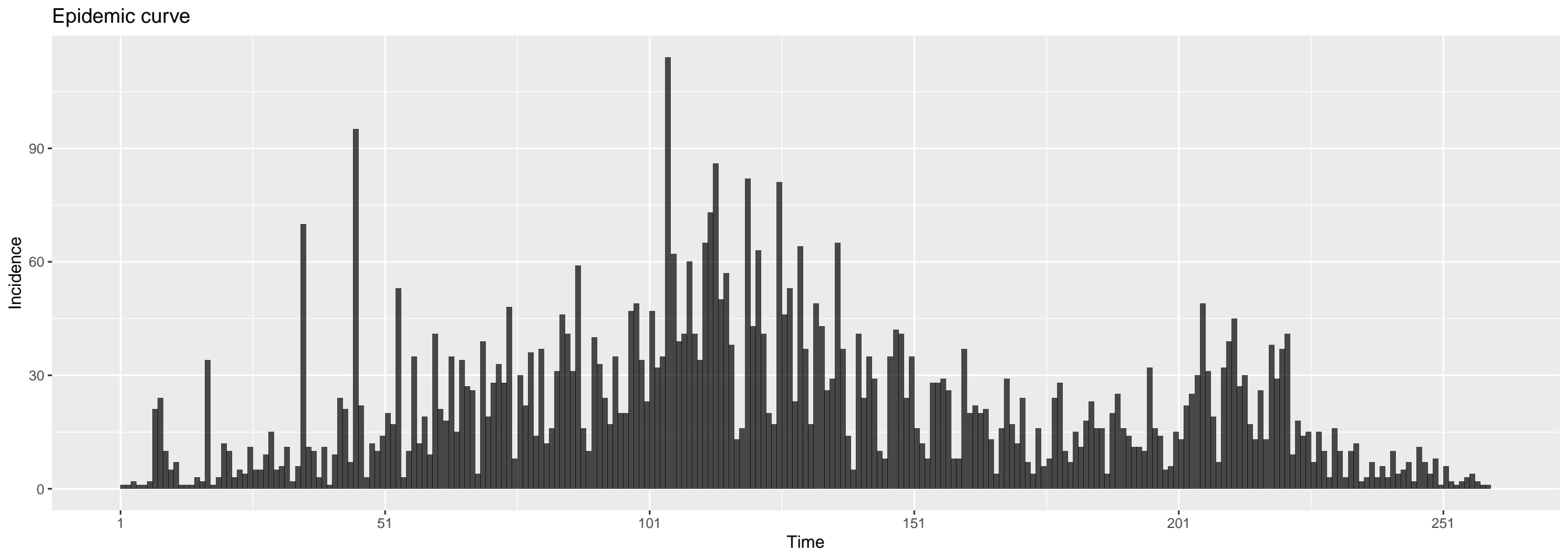

Supplement: Supplementary file 1 [file vaccines-09-00837-s001.zip › Supplementary_material/Supplementary Data S3/summary_plot/who-plot- AR .pdf]

Estimated R

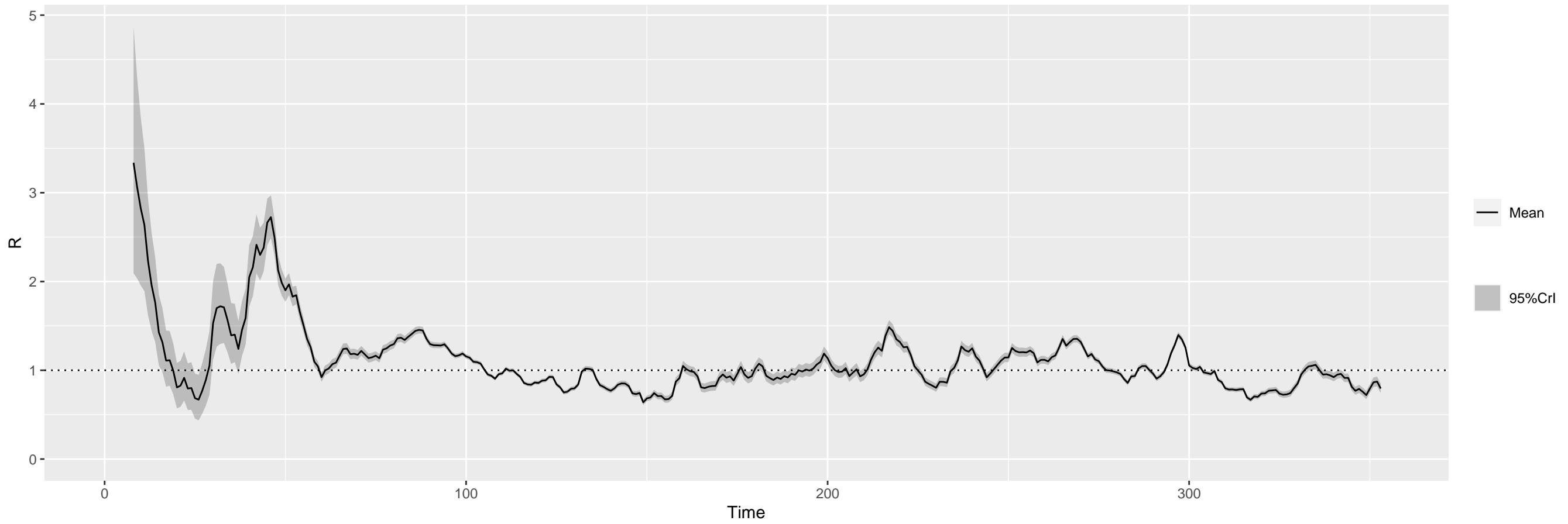

Epidemic curve

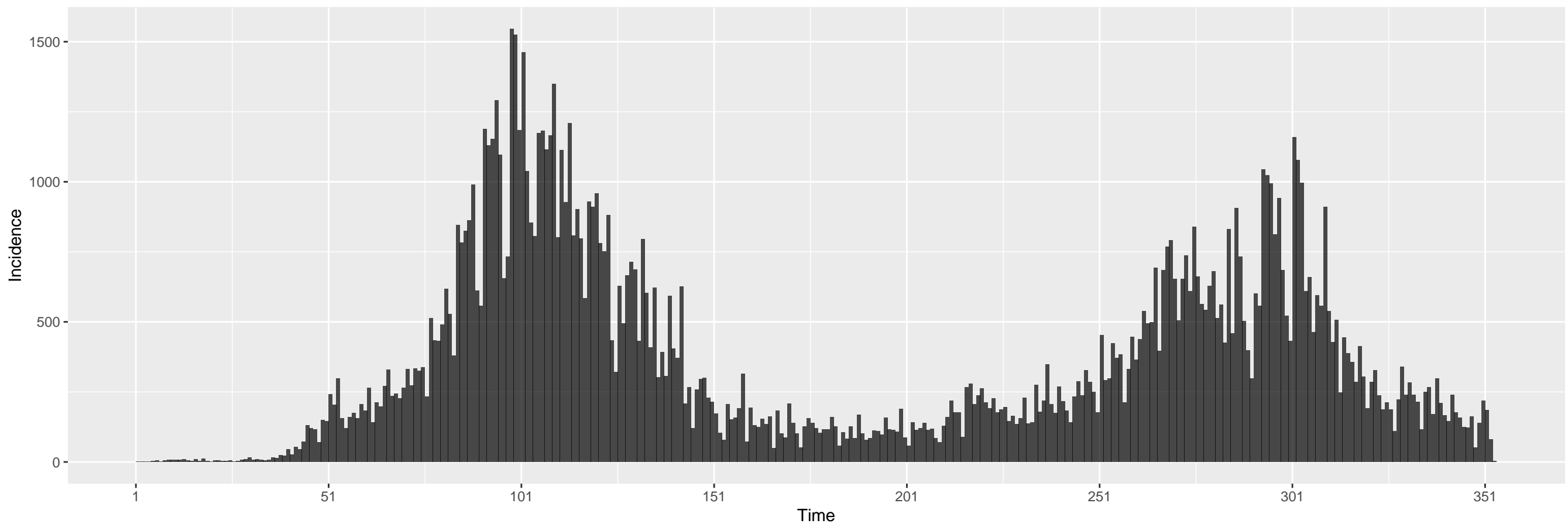

Supplement: Supplementary file 1 [file vaccines-09-00837-s001.zip › Supplementary_material/Supplementary Data S3/summary_plot/who-plot- AT .pdf]

Estimated R

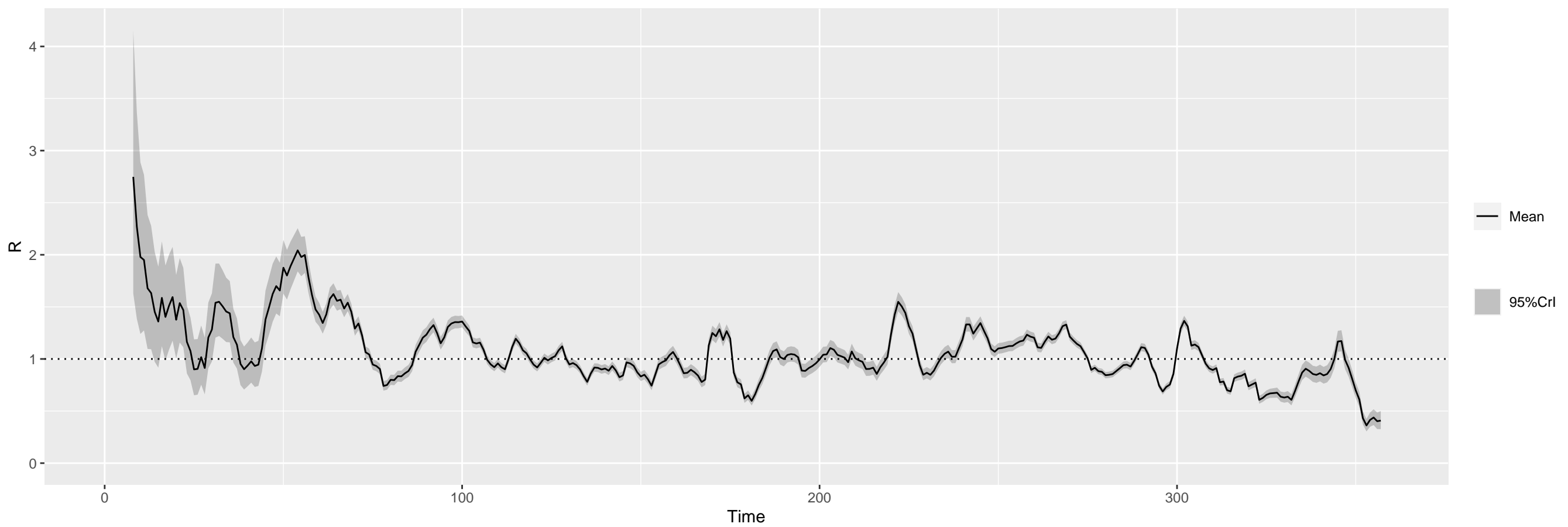

Epidemic curve

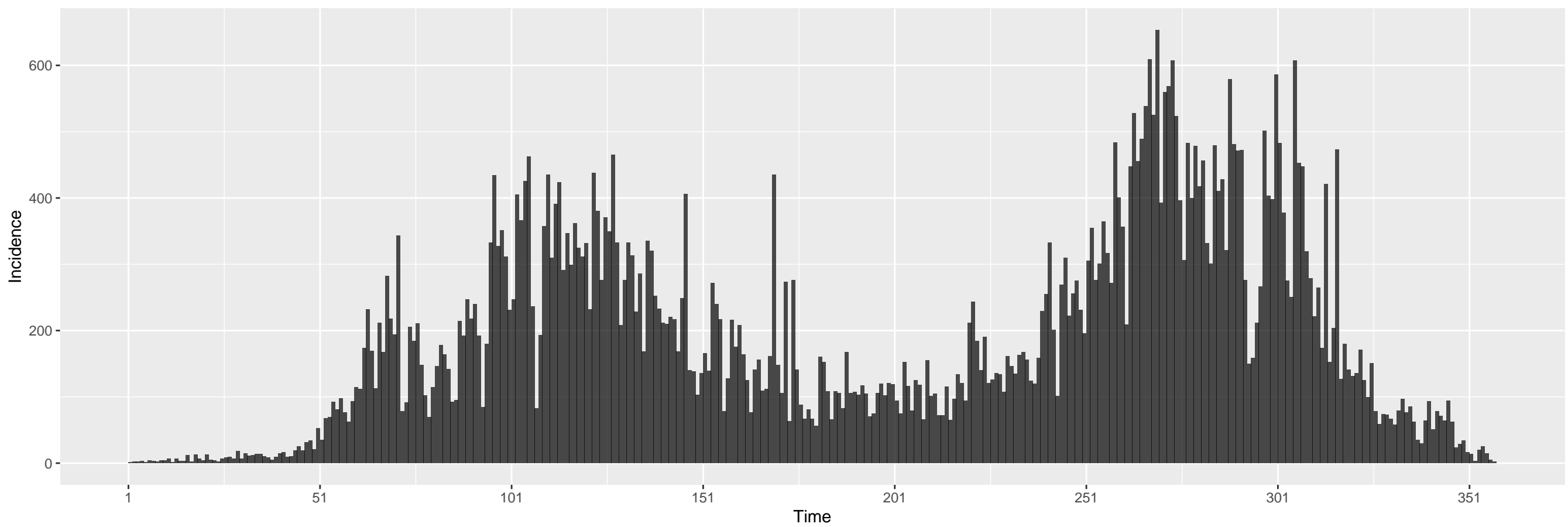

Supplement: Supplementary file 1 [file vaccines-09-00837-s001.zip › Supplementary_material/Supplementary Data S3/summary_plot/who-plot- BL .pdf]

Estimated R

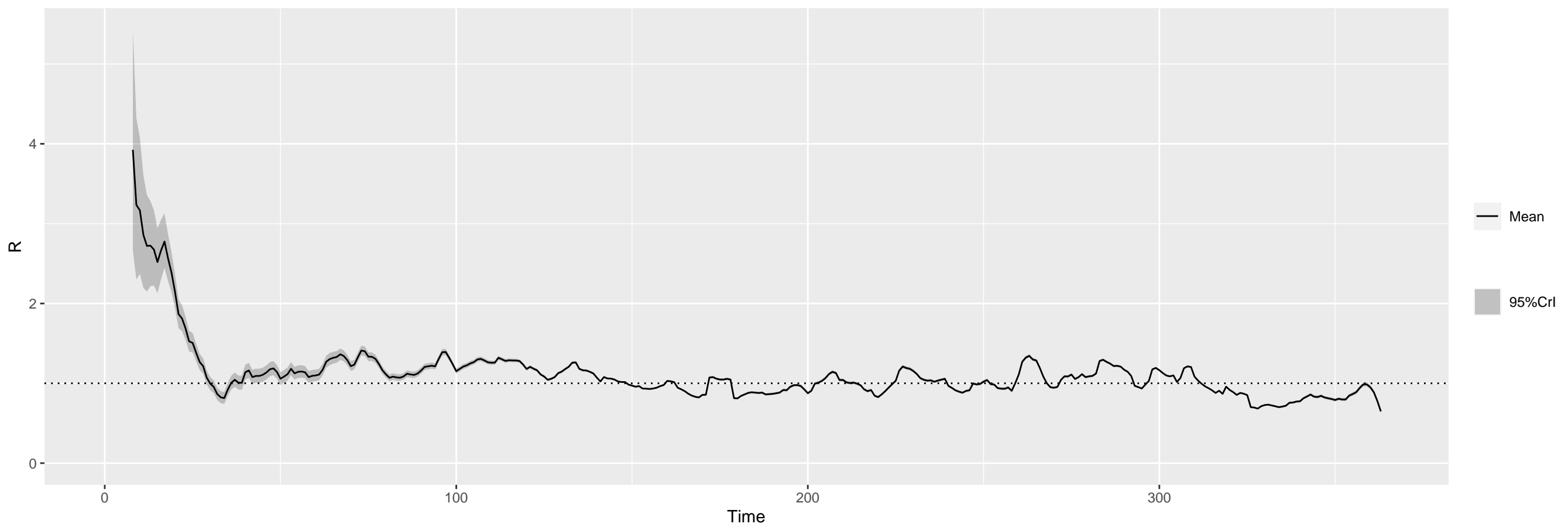

Epidemic curve

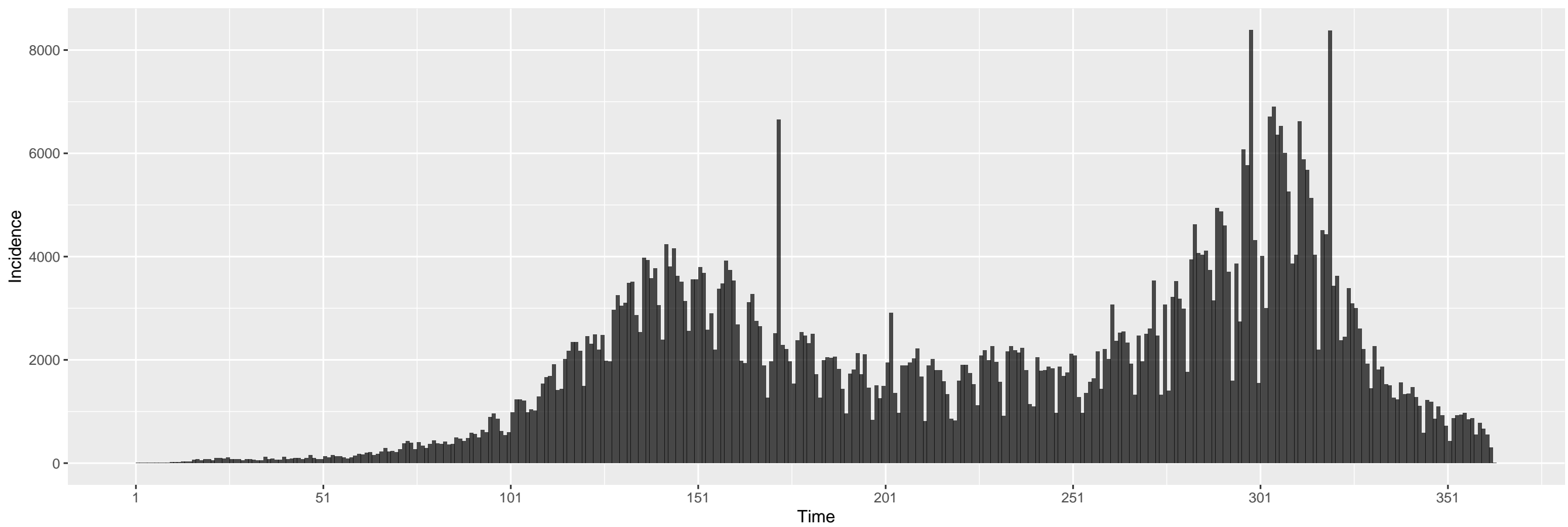

Supplement: Supplementary file 1 [file vaccines-09-00837-s001.zip › Supplementary_material/Supplementary Data S3/summary_plot/who-plot- BO .pdf]

Estimated R

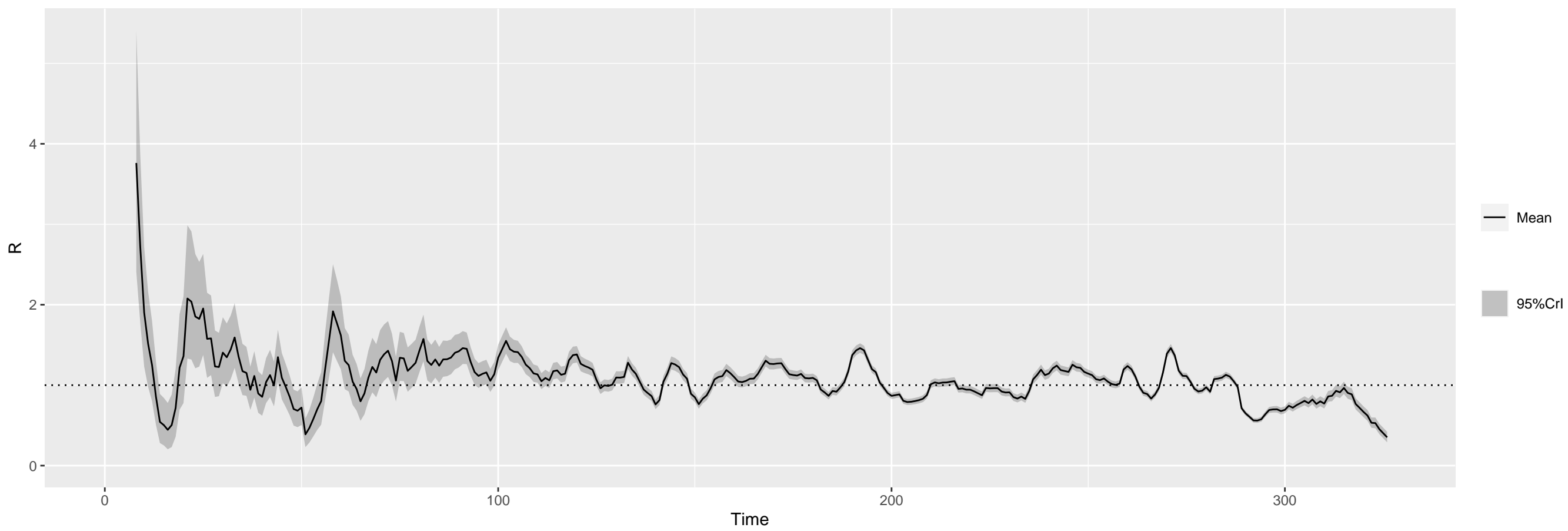

Epidemic curve

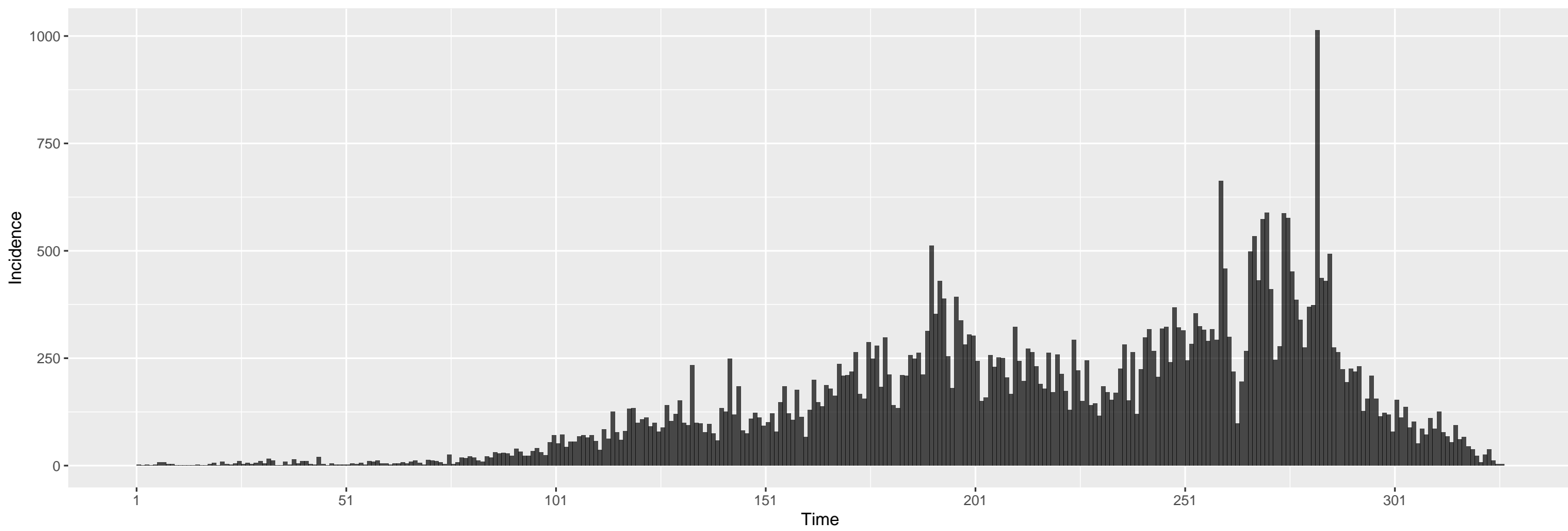

Supplement: Supplementary file 1 [file vaccines-09-00837-s001.zip › Supplementary_material/Supplementary Data S3/summary_plot/who-plot- BY .pdf]

Estimated R

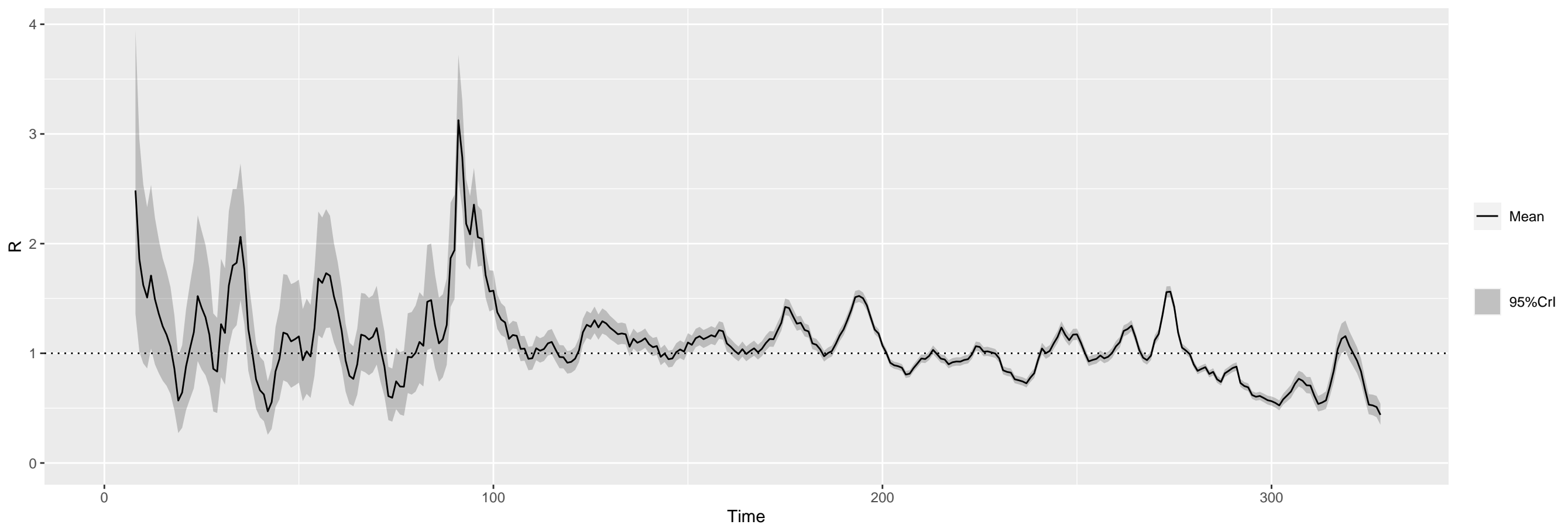

Epidemic curve

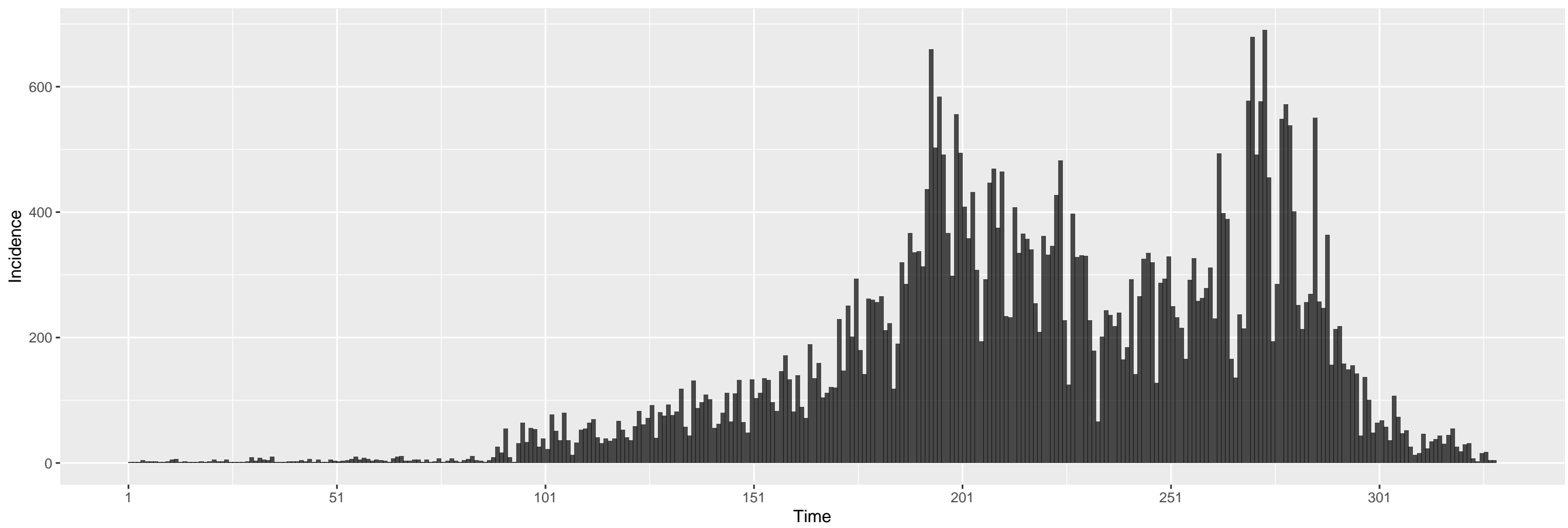

Supplement: Supplementary file 1 [file vaccines-09-00837-s001.zip › Supplementary_material/Supplementary Data S3/summary_plot/who-plot- CA .pdf]

Estimated R

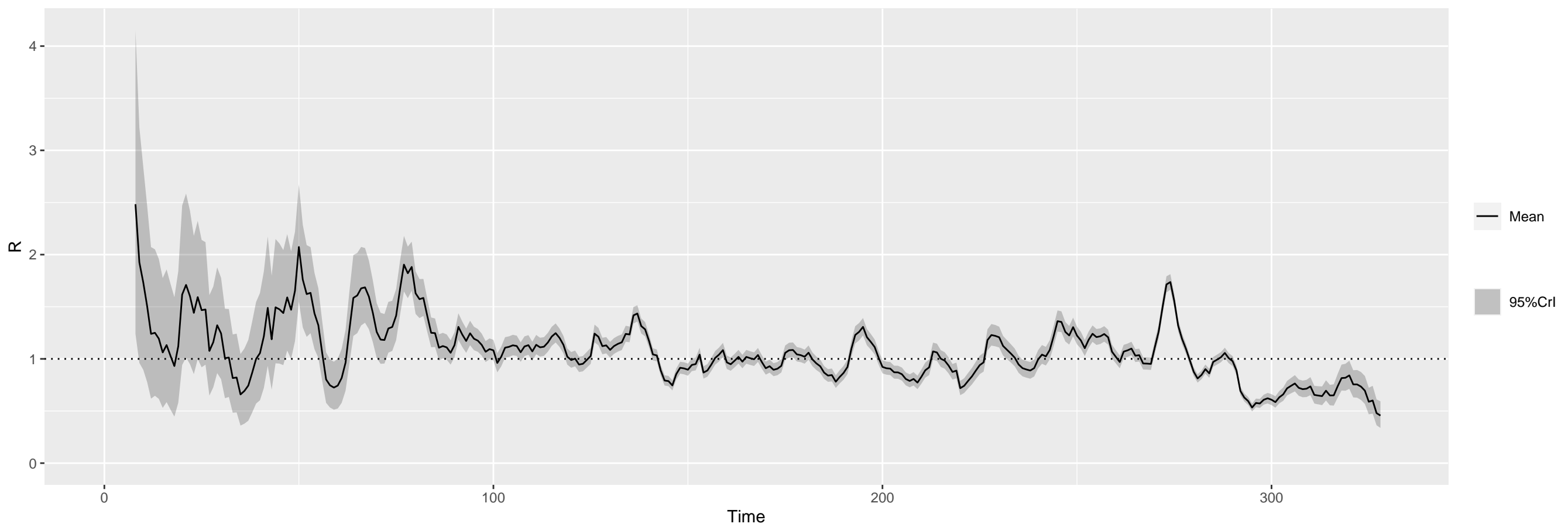

Epidemic curve

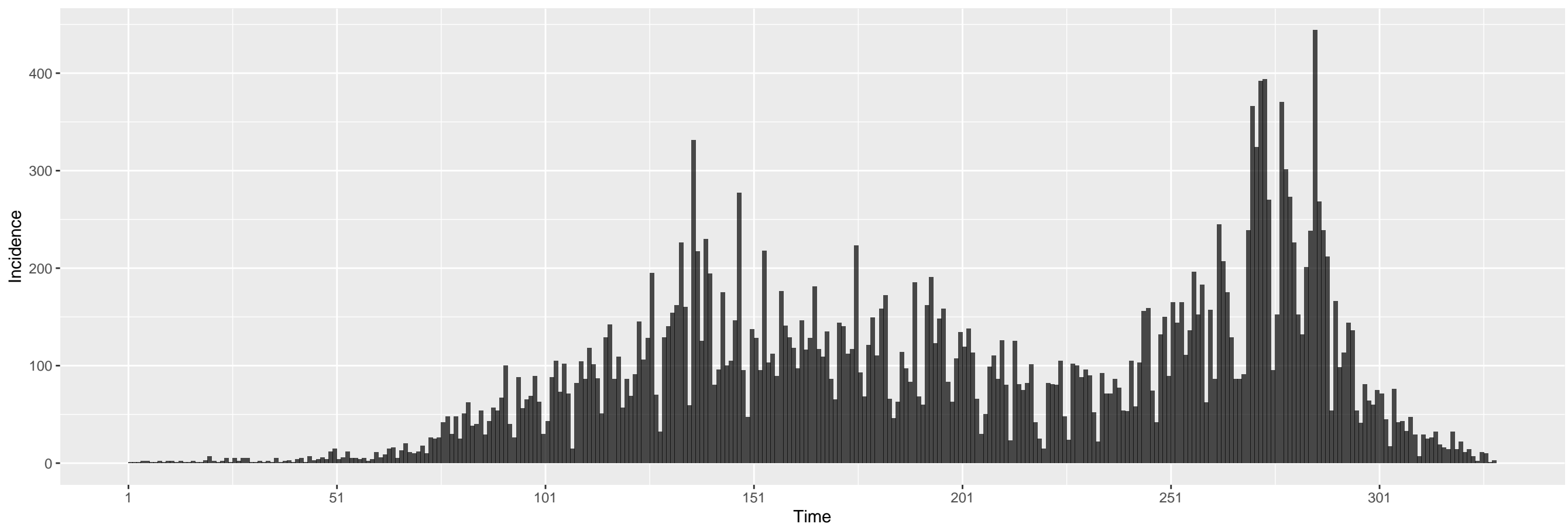

Supplement: Supplementary file 1 [file vaccines-09-00837-s001.zip › Supplementary_material/Supplementary Data S3/summary_plot/who-plot- CC .pdf]

Estimated R

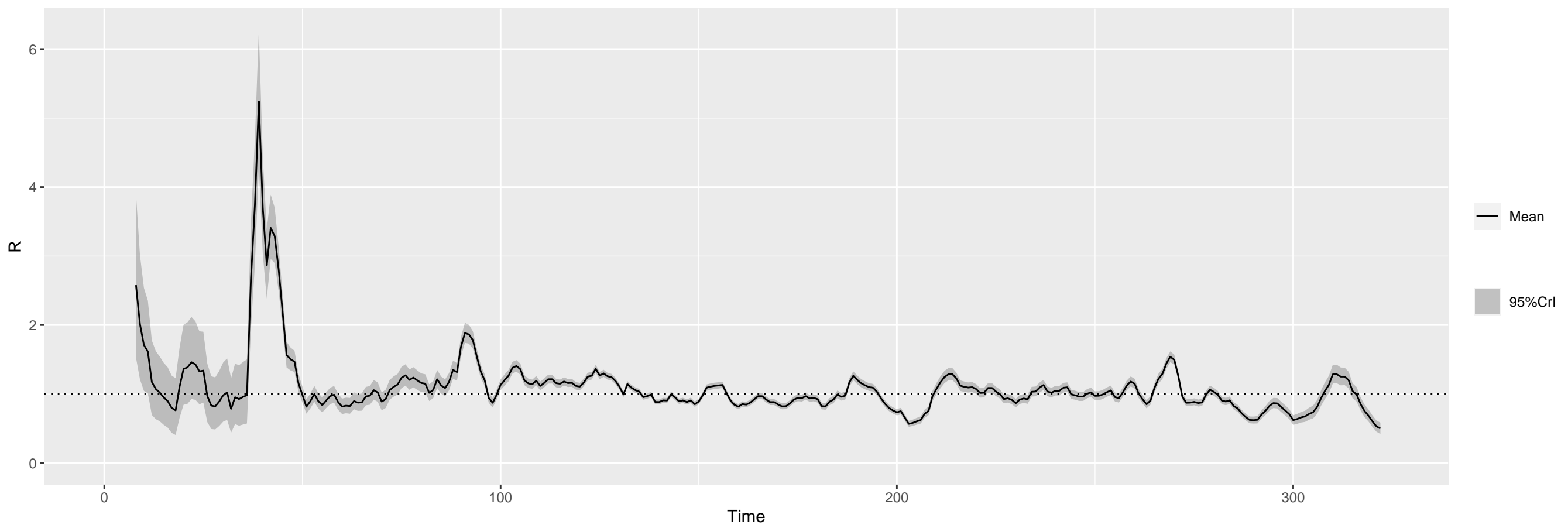

Epidemic curve

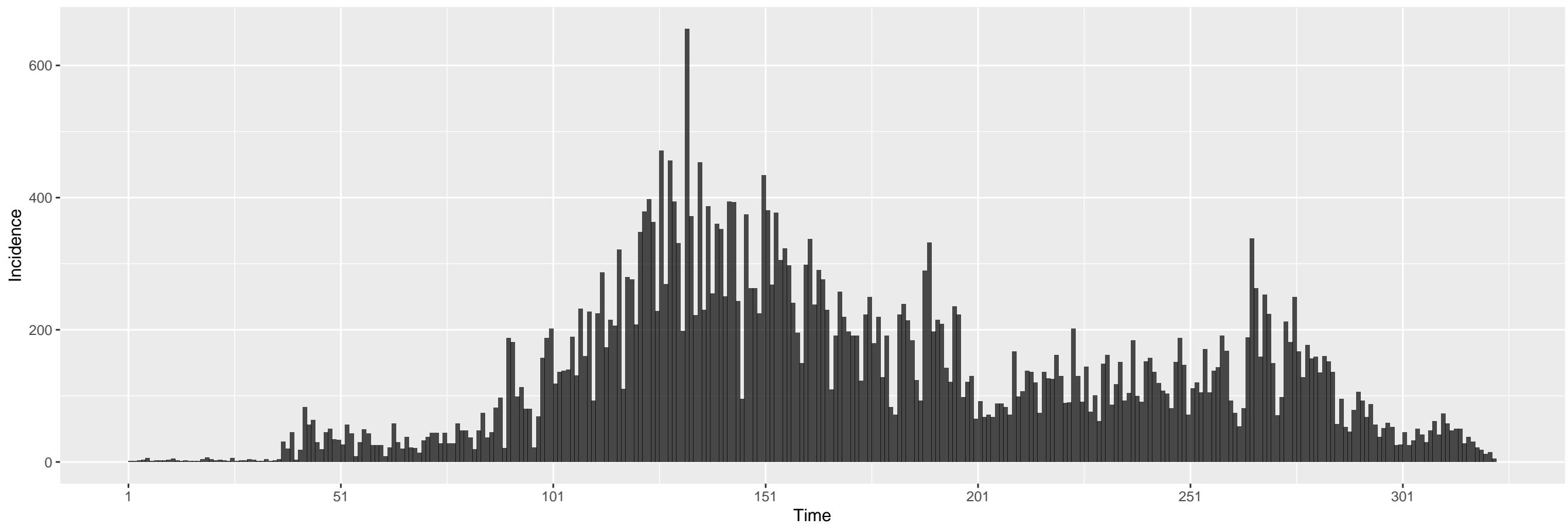

Supplement: Supplementary file 1 [file vaccines-09-00837-s001.zip › Supplementary_material/Supplementary Data S3/summary_plot/who-plot- CE .pdf]

Estimated R

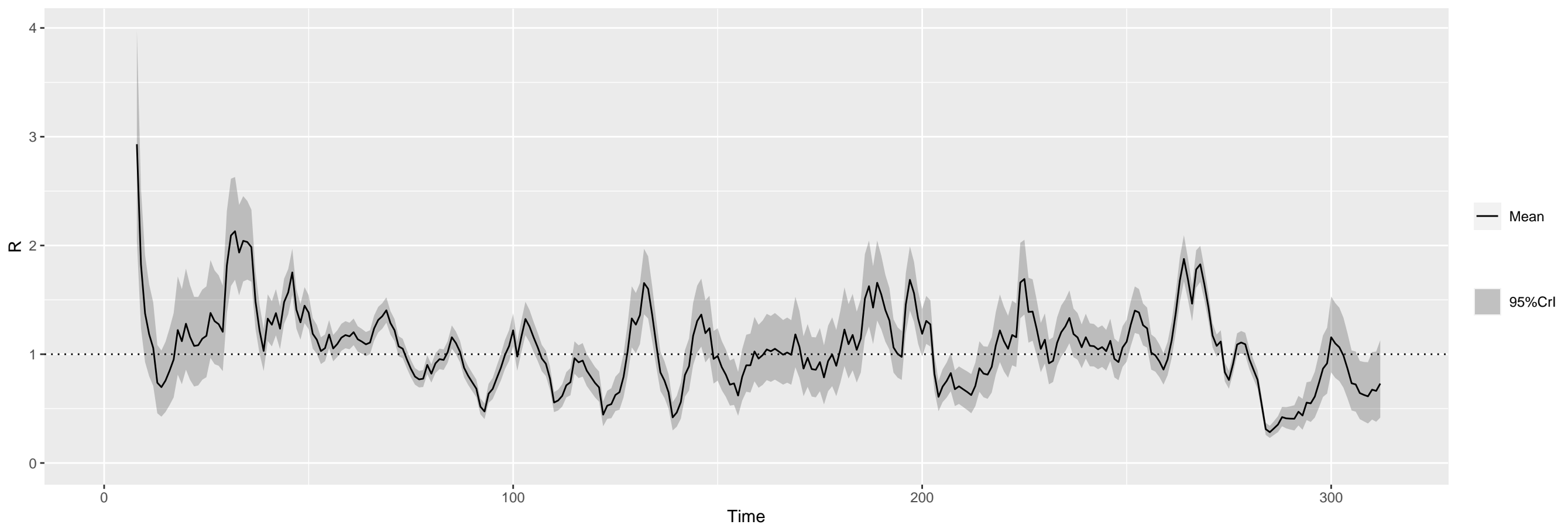

Epidemic curve

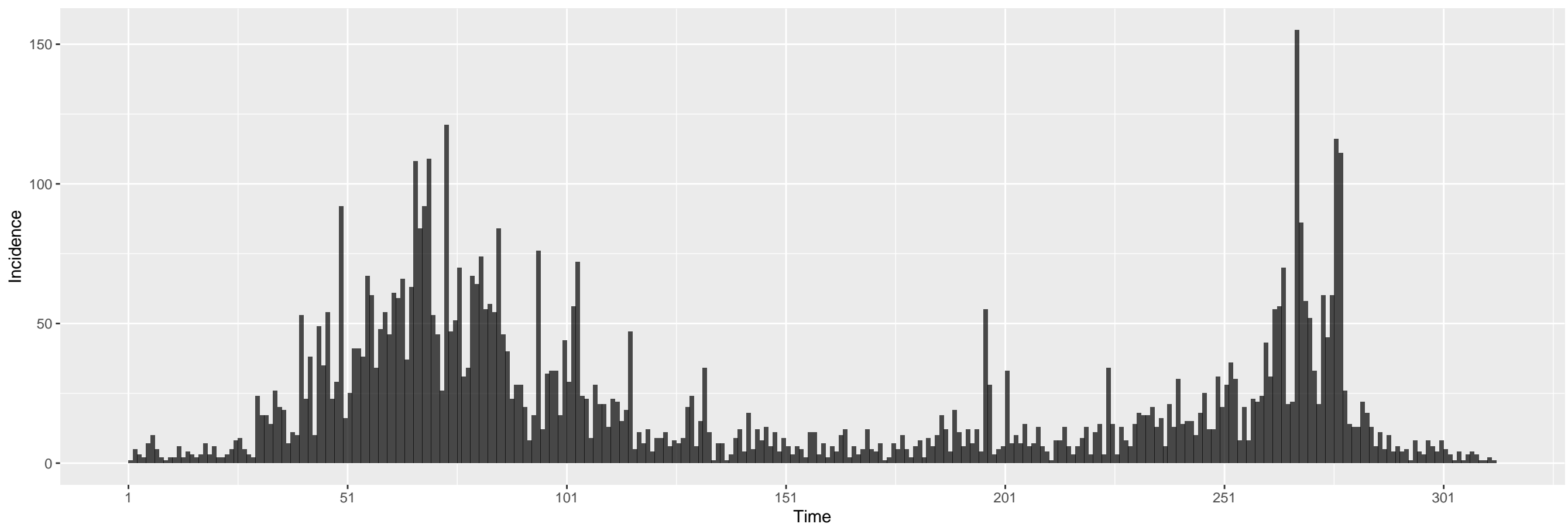

Supplement: Supplementary file 1 [file vaccines-09-00837-s001.zip › Supplementary_material/Supplementary Data S3/summary_plot/who-plot- CH .pdf]

Estimated R

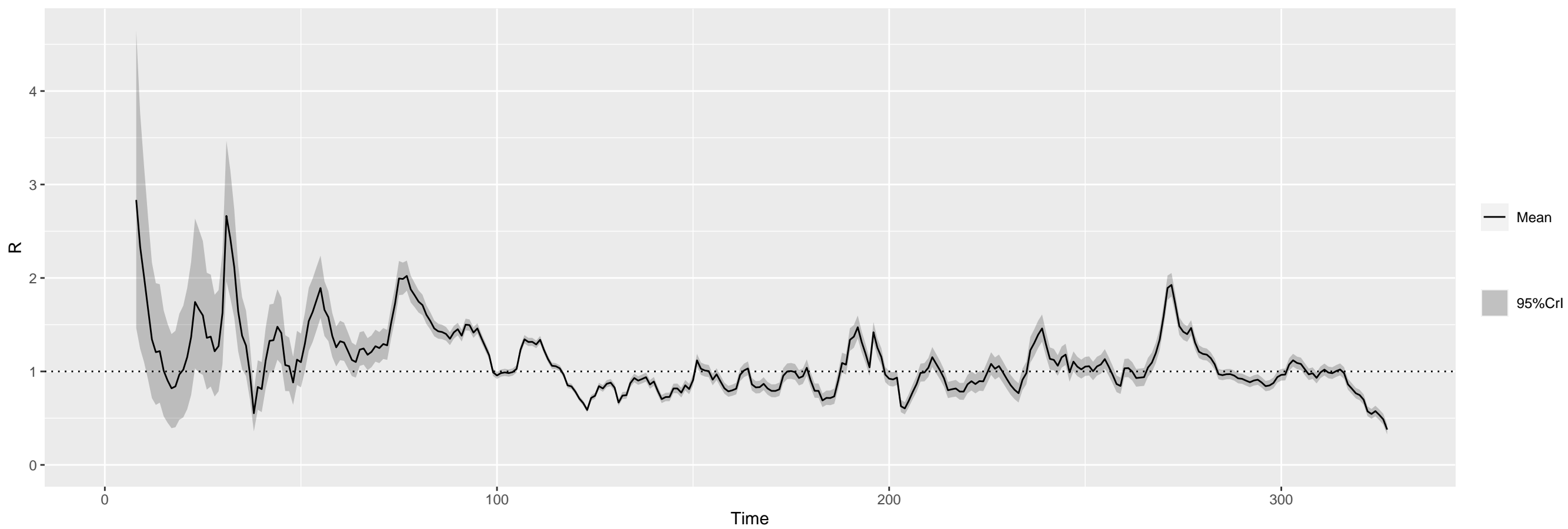

Epidemic curve

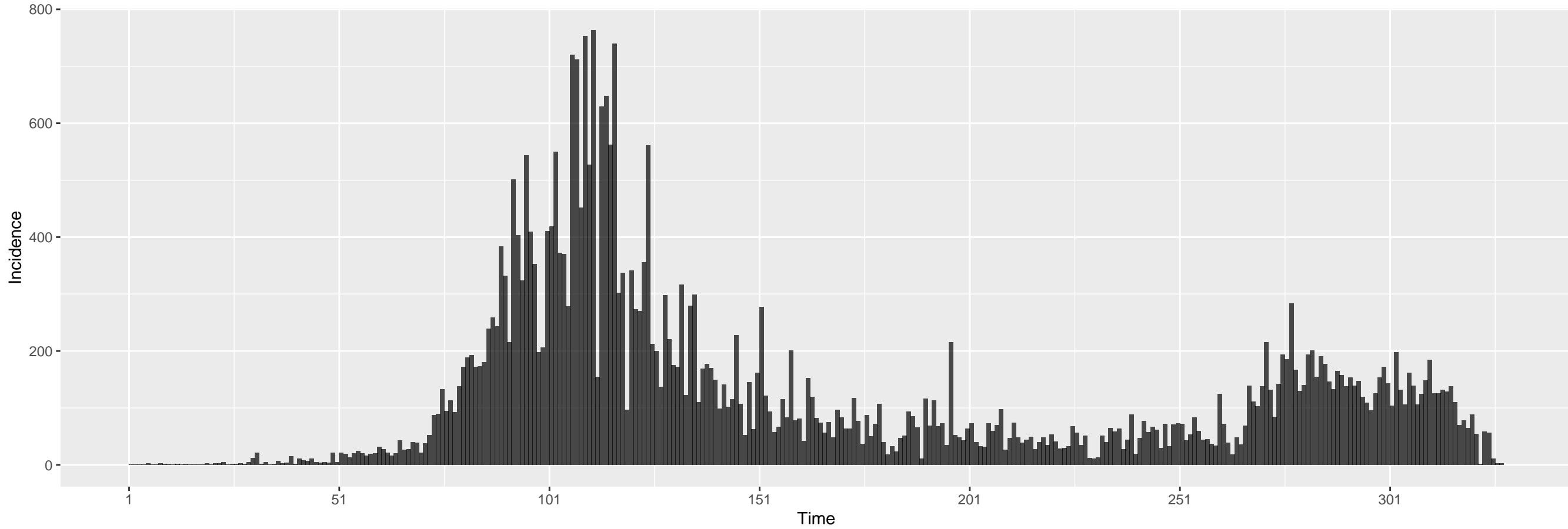

Supplement: Supplementary file 1 [file vaccines-09-00837-s001.zip › Supplementary_material/Supplementary Data S3/summary_plot/who-plot- CO .pdf]

Estimated R

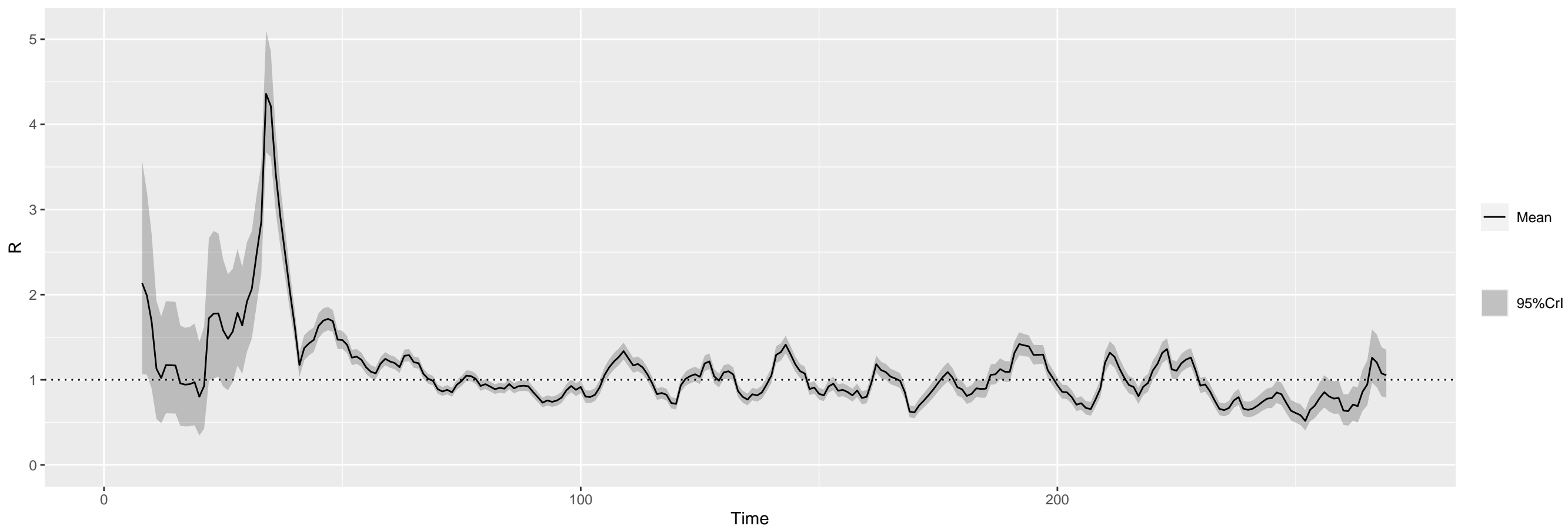

Epidemic curve

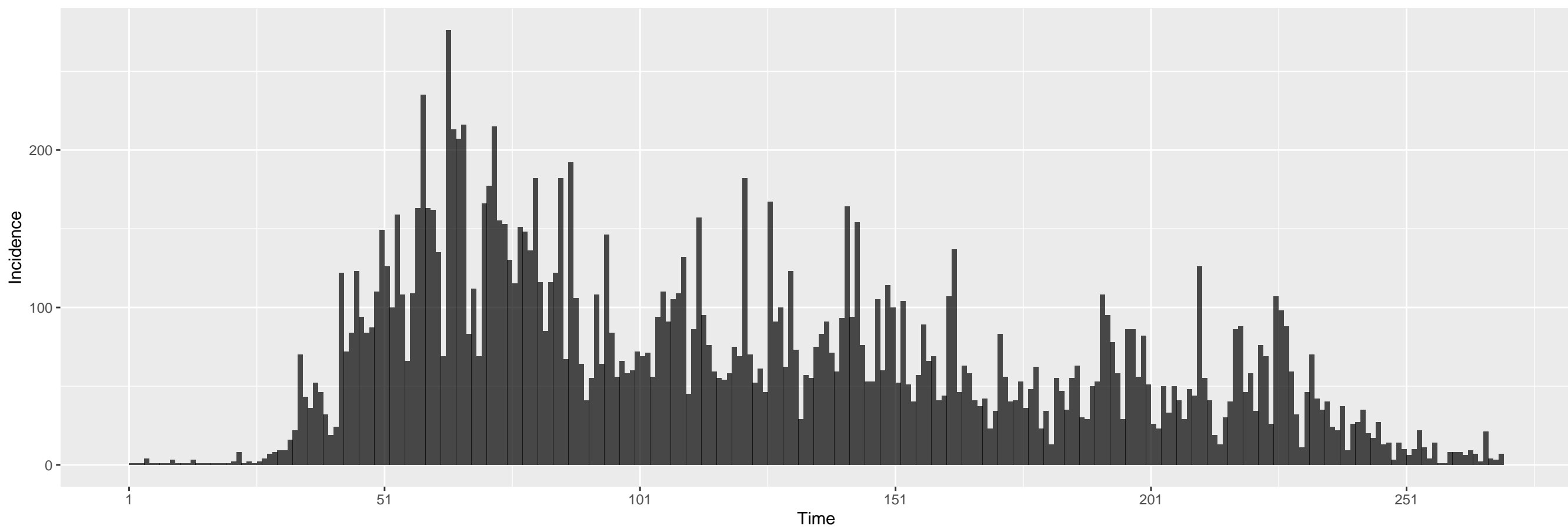

Supplement: Supplementary file 1 [file vaccines-09-00837-s001.zip › Supplementary_material/Supplementary Data S3/summary_plot/who-plot- CQ .pdf]

Estimated R

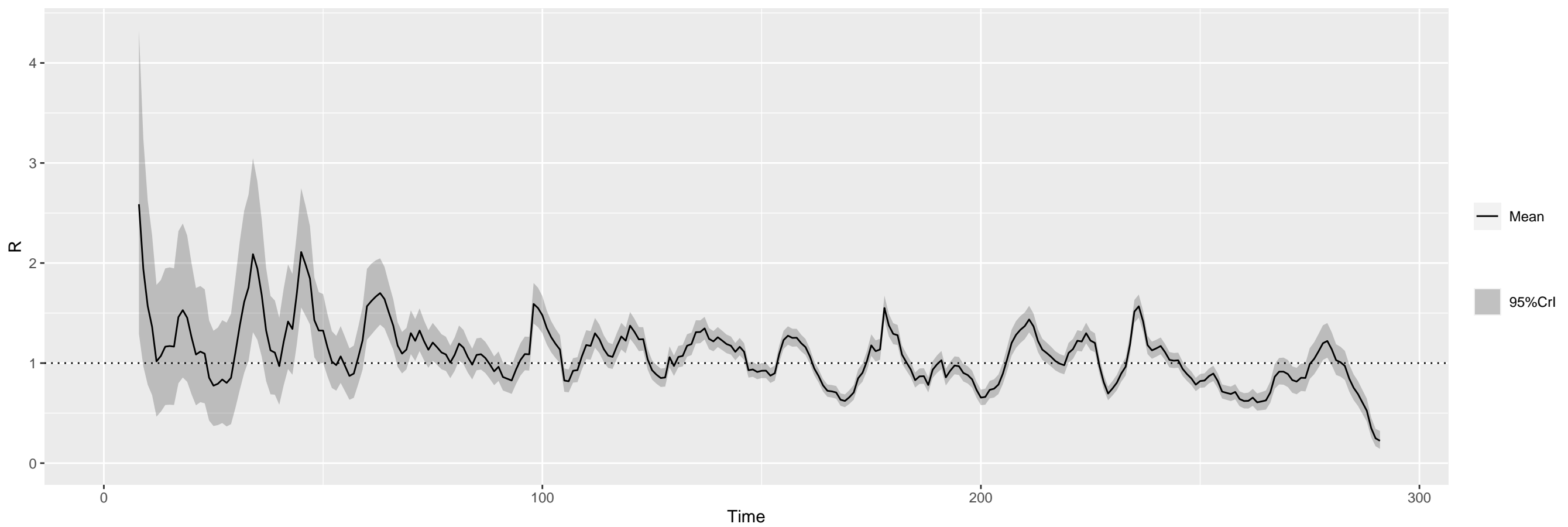

Epidemic curve

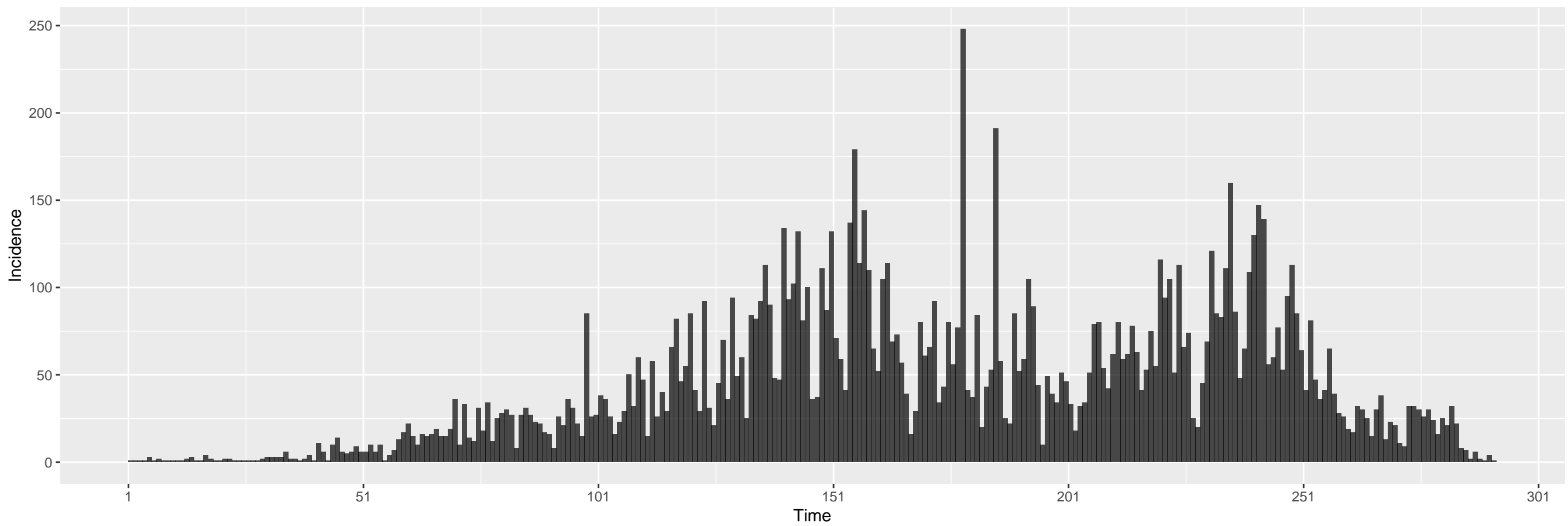

Supplement: Supplementary file 1 [file vaccines-09-00837-s001.zip › Supplementary_material/Supplementary Data S3/summary_plot/who-plot- CS .pdf]

Estimated R

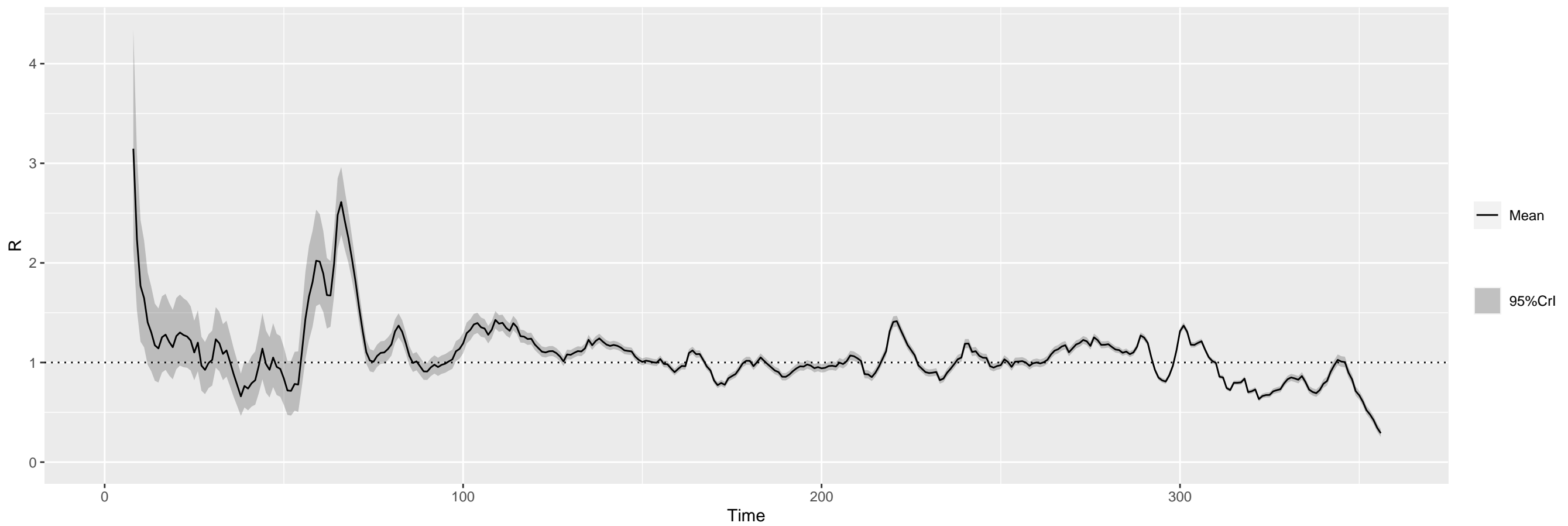

Epidemic curve

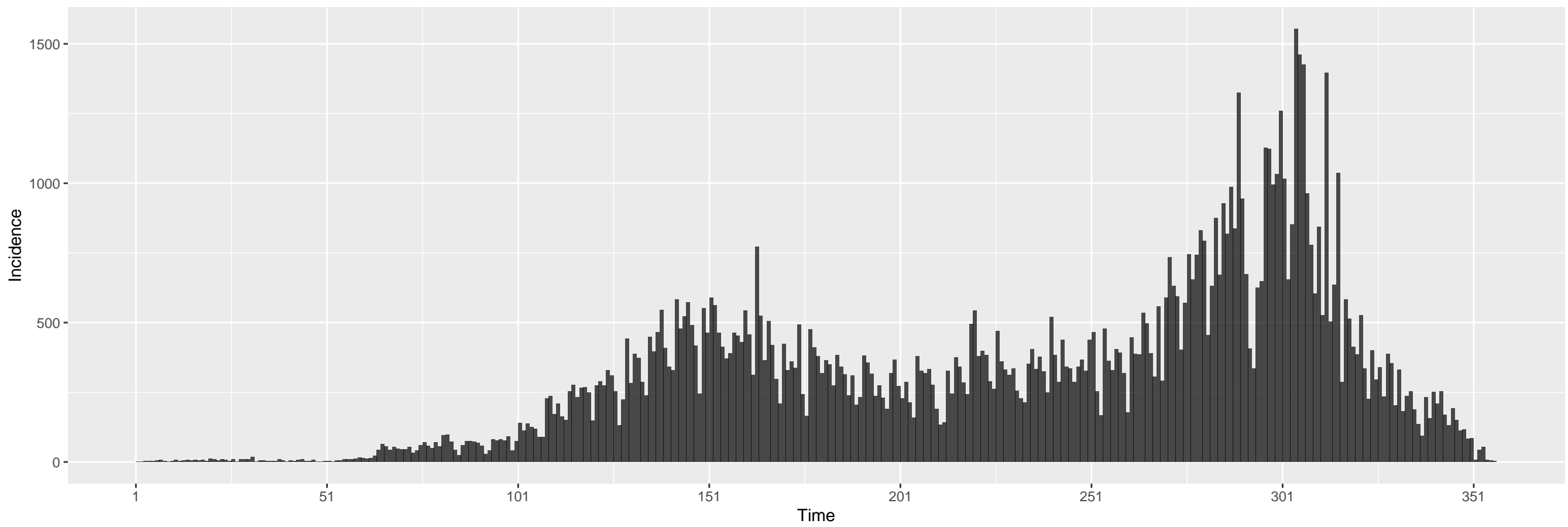

Supplement: Supplementary file 1 [file vaccines-09-00837-s001.zip › Supplementary_material/Supplementary Data S3/summary_plot/who-plot- CU .pdf]

Estimated R

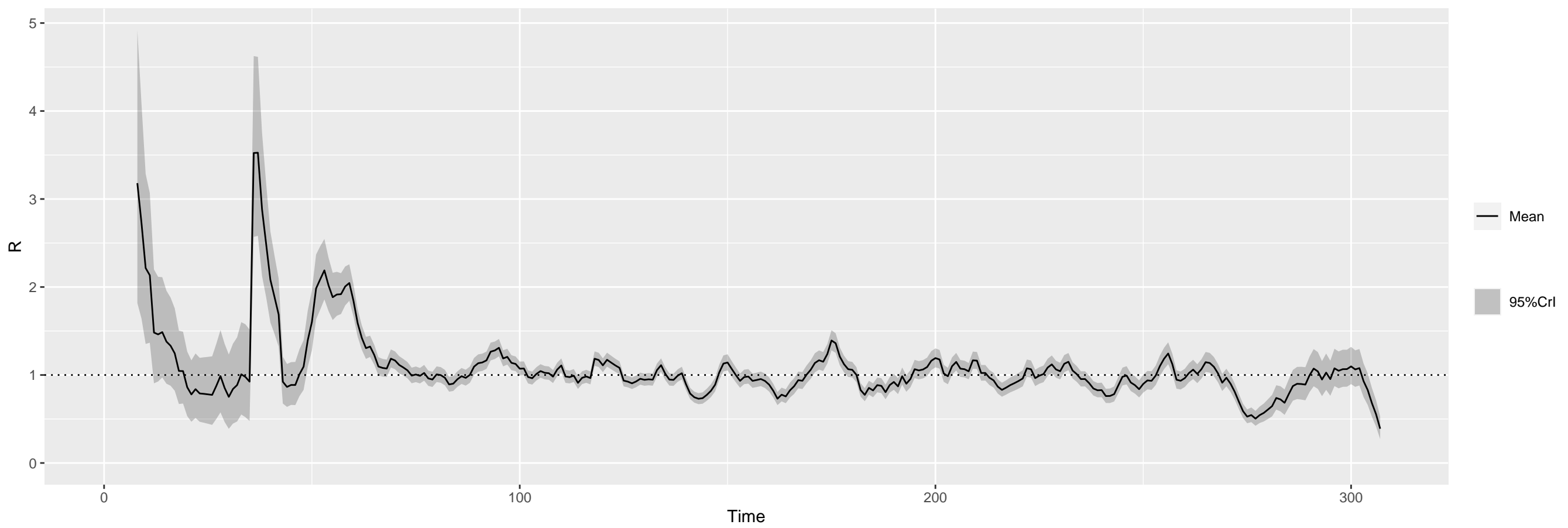

Epidemic curve

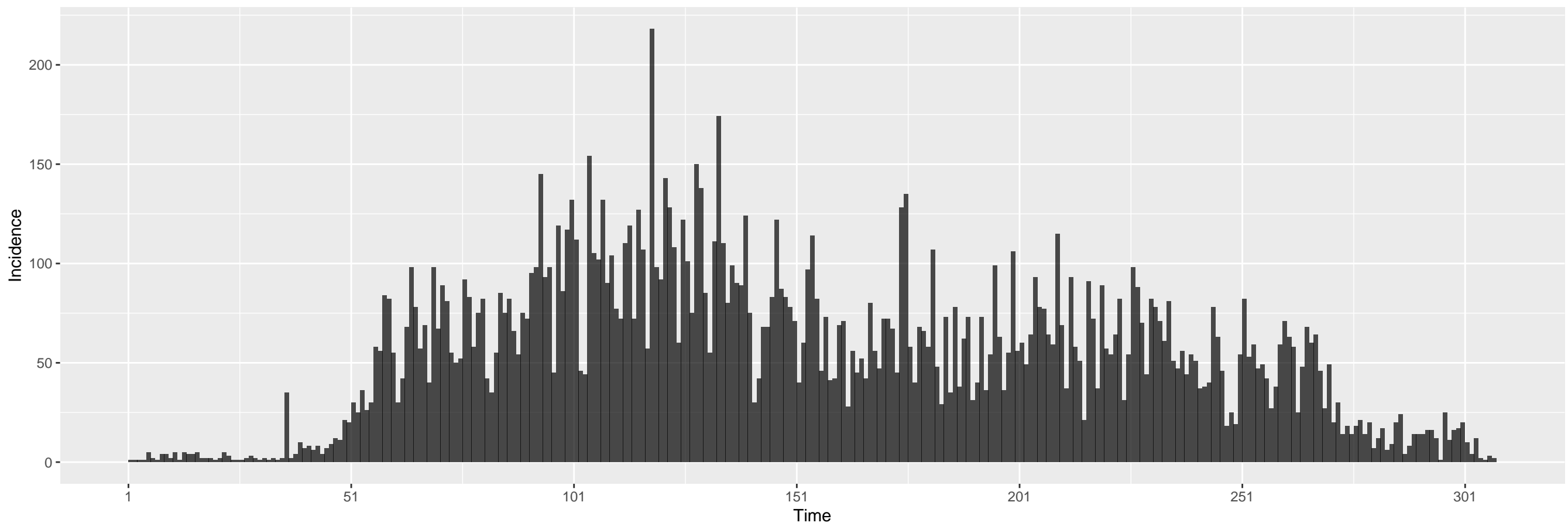

Supplement: Supplementary file 1 [file vaccines-09-00837-s001.zip › Supplementary_material/Supplementary Data S3/summary_plot/who-plot- GJ .pdf]

Estimated R

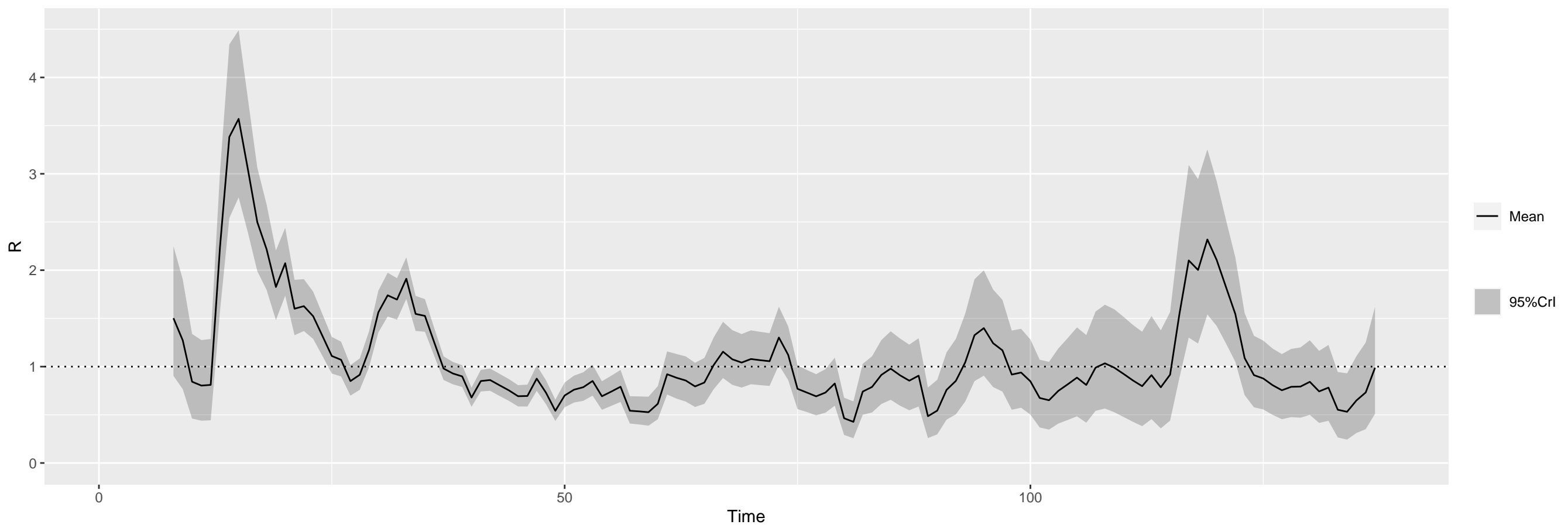

Epidemic curve

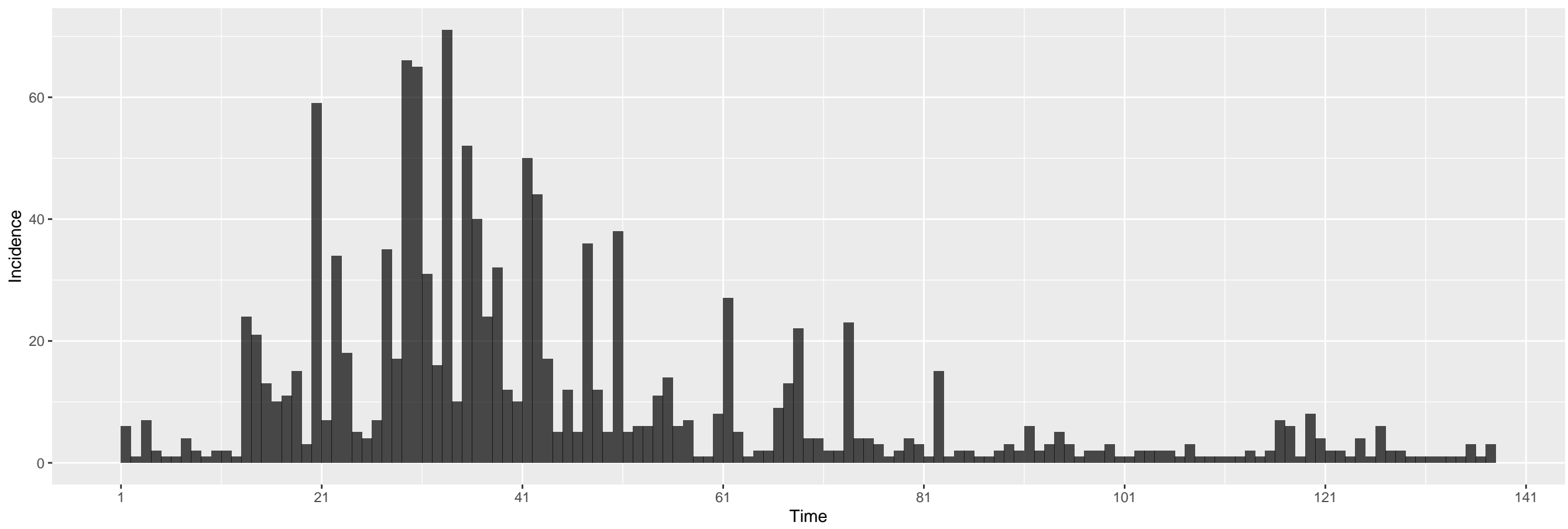

Supplement: Supplementary file 1 [file vaccines-09-00837-s001.zip › Supplementary_material/Supplementary Data S3/summary_plot/who-plot- GN .pdf]

Estimated R

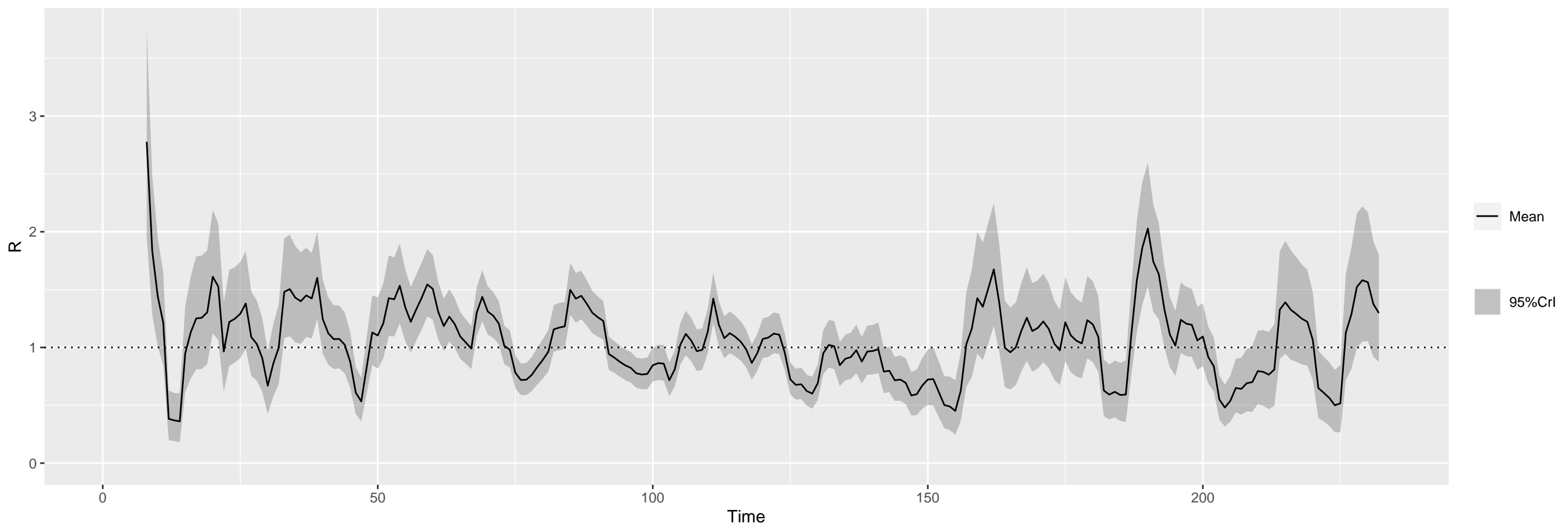

Epidemic curve

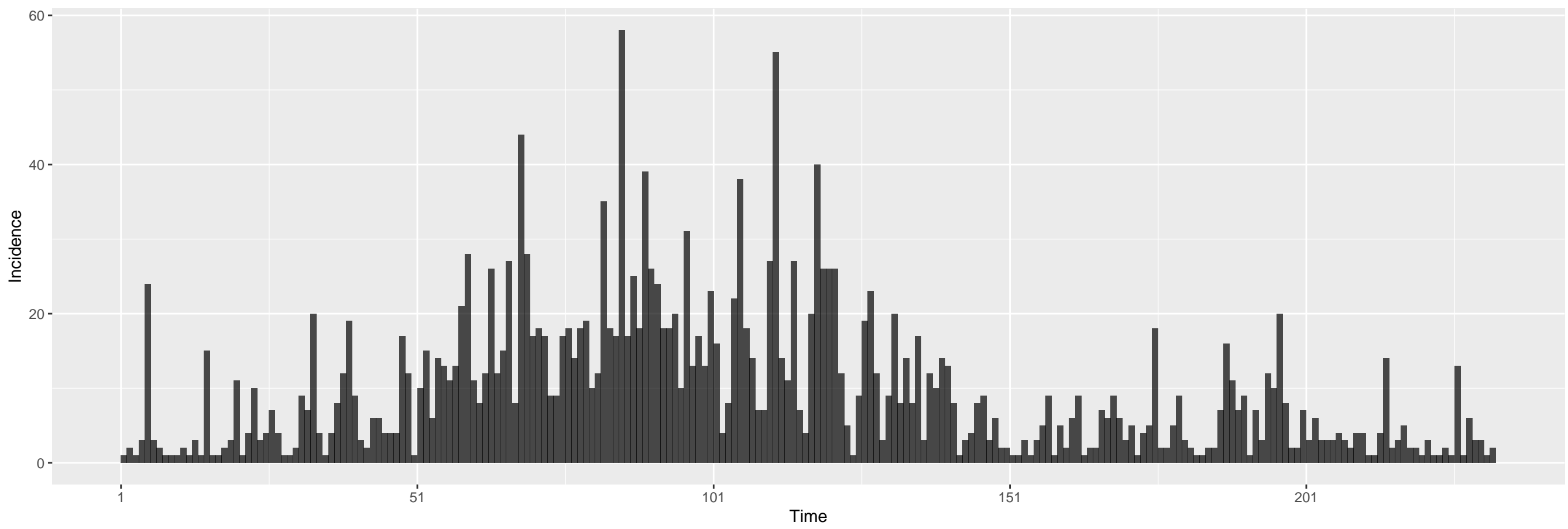

Supplement: Supplementary file 1 [file vaccines-09-00837-s001.zip › Supplementary_material/Supplementary Data S3/summary_plot/who-plot- GV .pdf]

Estimated R

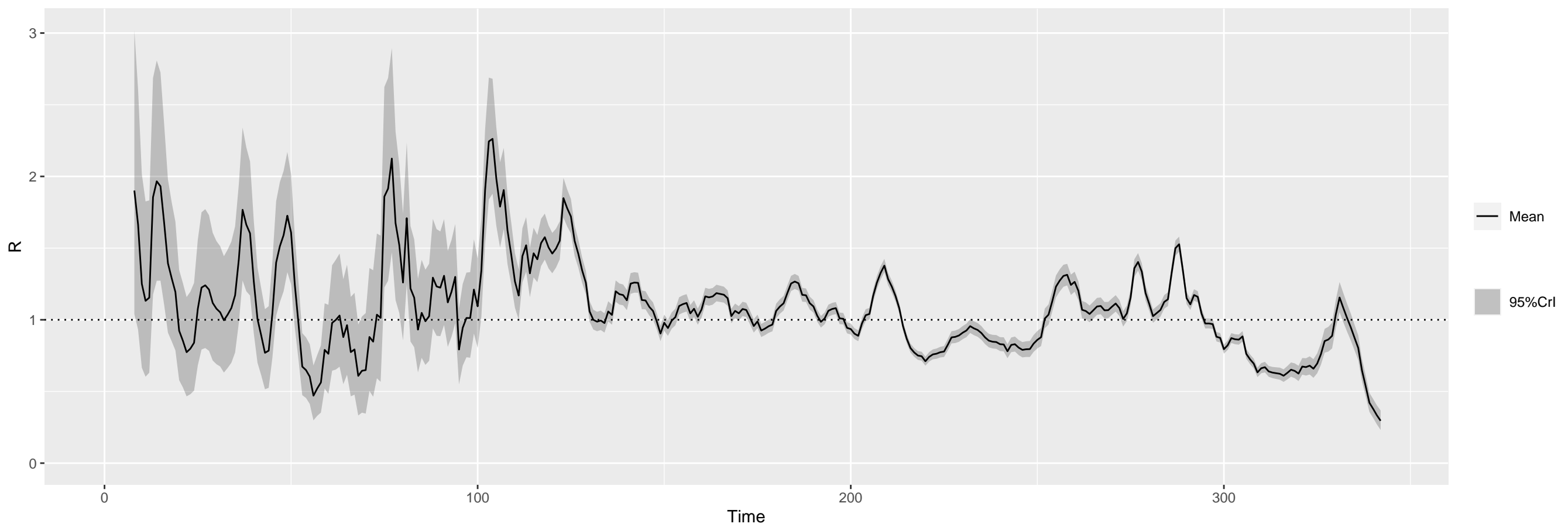

Epidemic curve

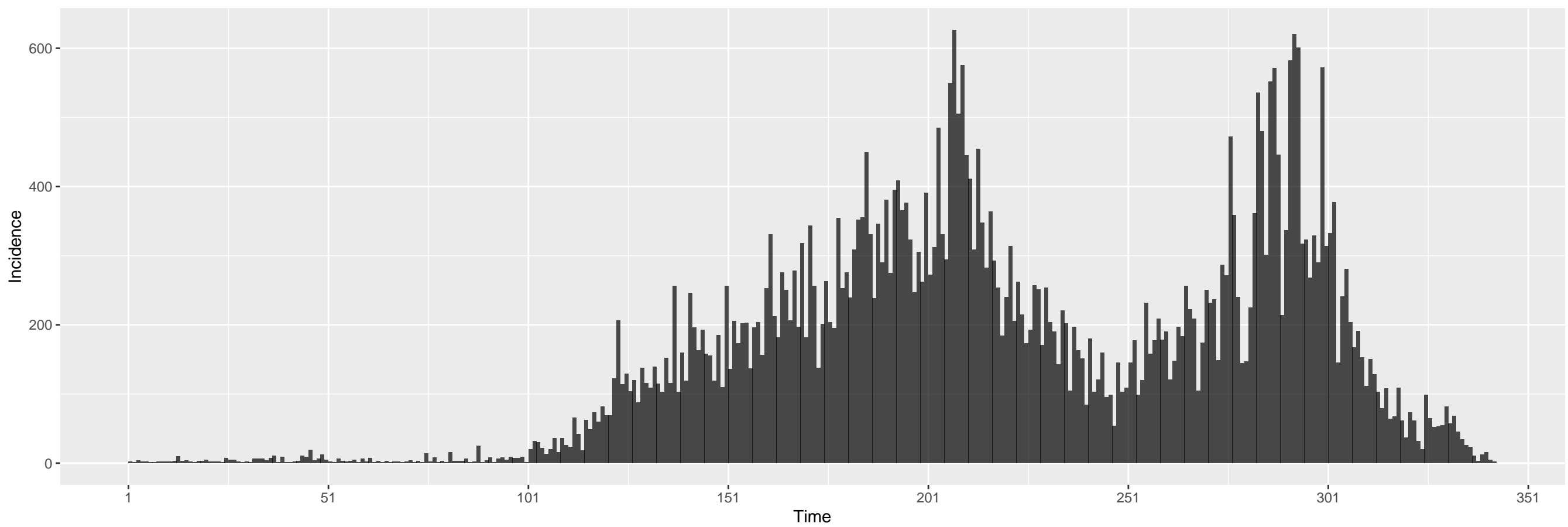

Supplement: Supplementary file 1 [file vaccines-09-00837-s001.zip › Supplementary_material/Supplementary Data S3/summary_plot/who-plot- HU .pdf]

Estimated R

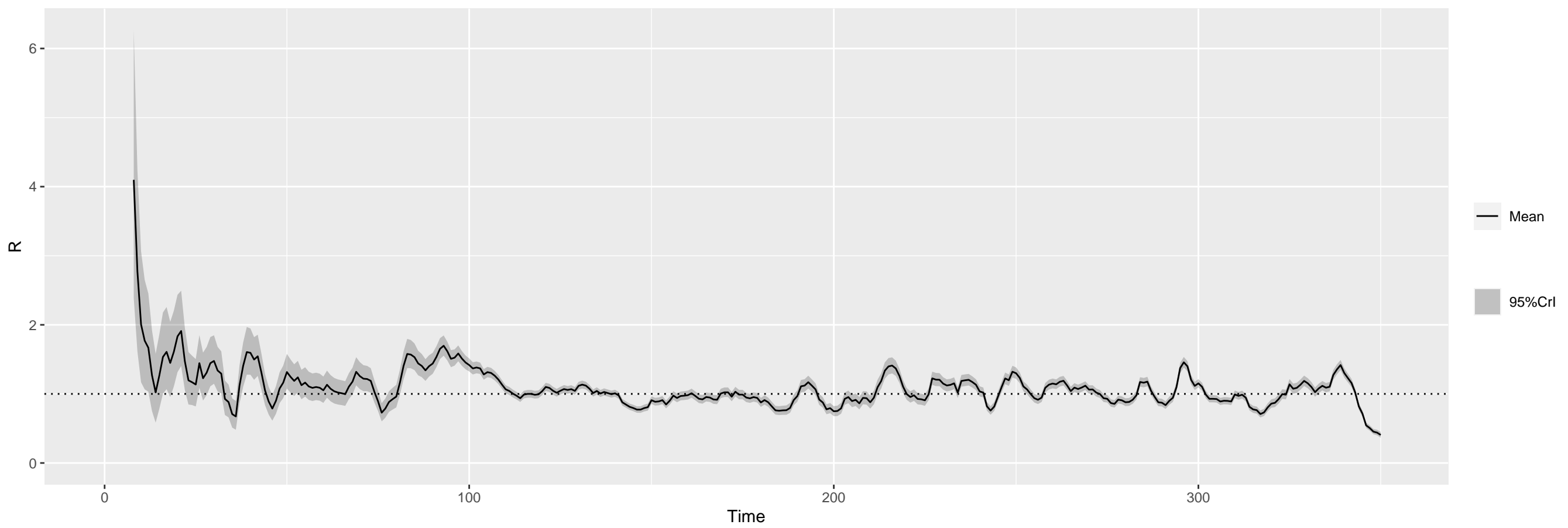

Epidemic curve

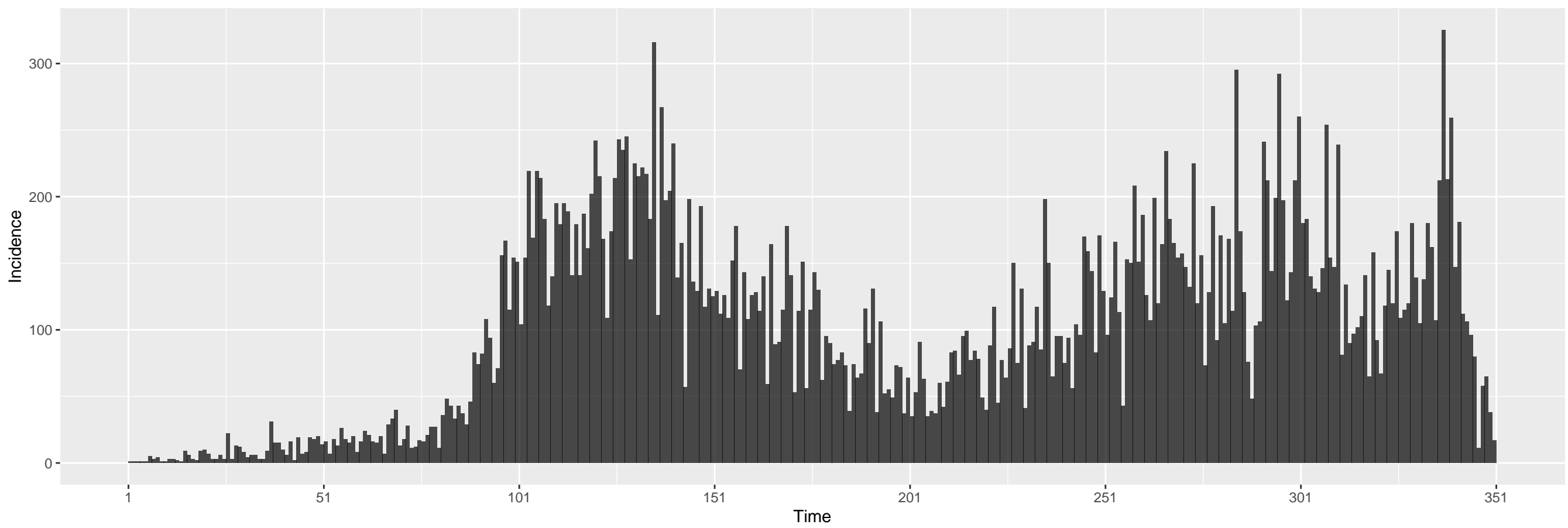

Supplement: Supplementary file 1 [file vaccines-09-00837-s001.zip › Supplementary_material/Supplementary Data S3/summary_plot/who-plot- MA .pdf]

Estimated R

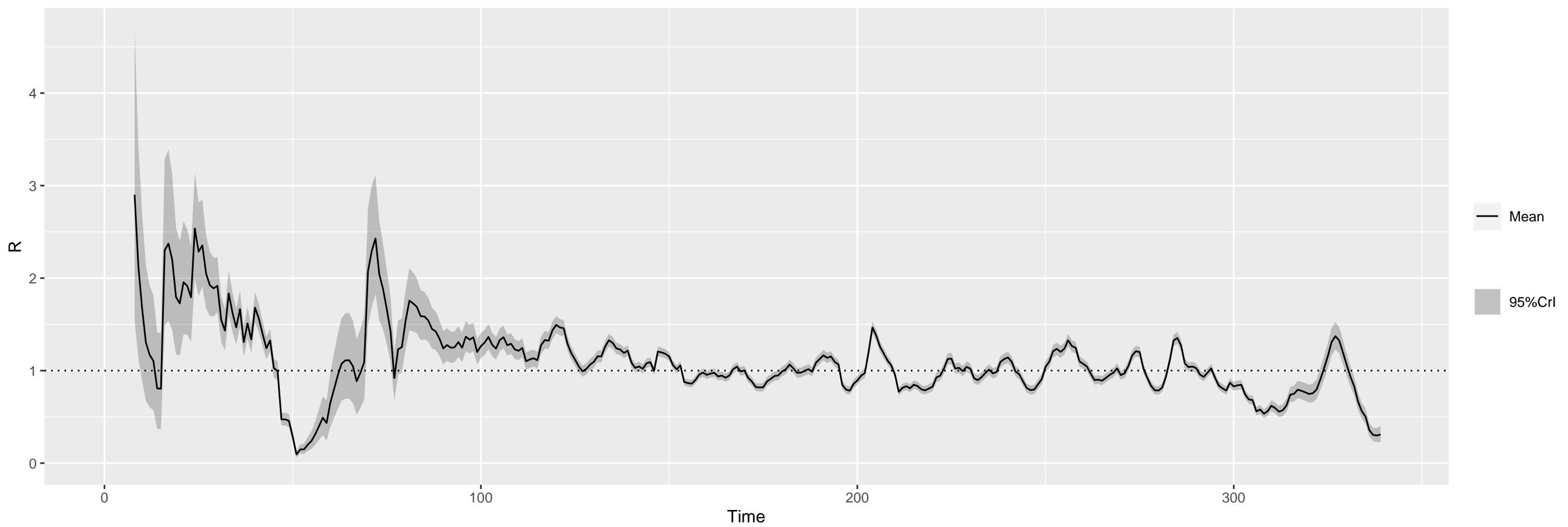

Epidemic curve

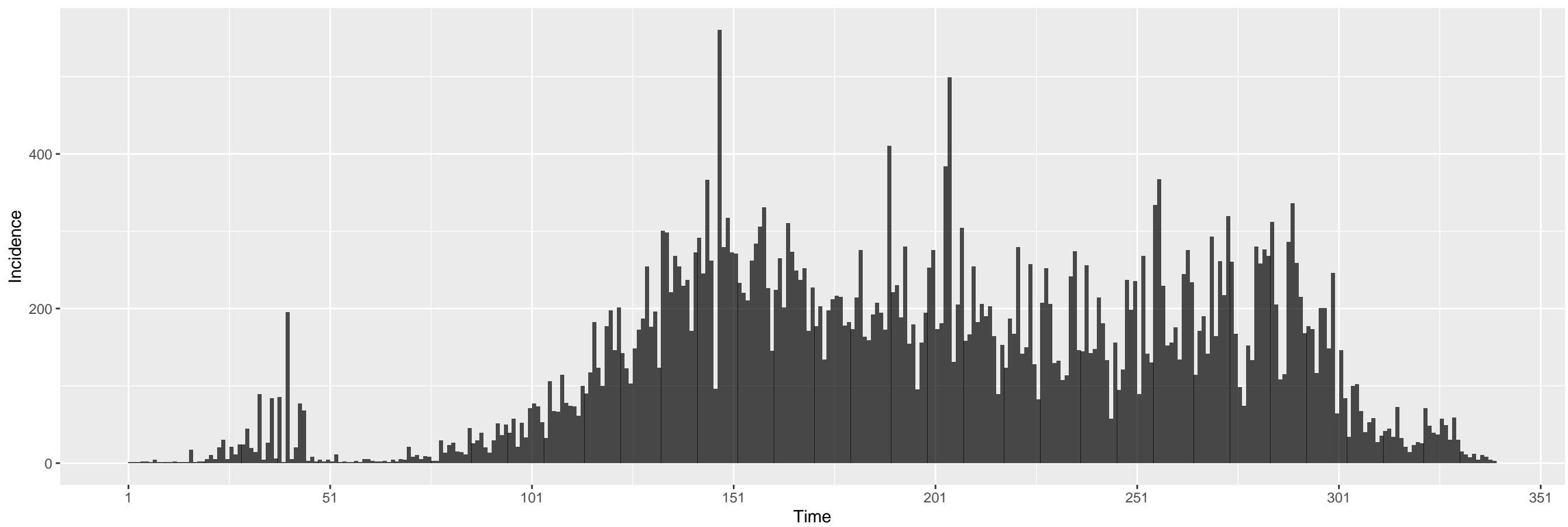

Supplement: Supplementary file 1 [file vaccines-09-00837-s001.zip › Supplementary_material/Supplementary Data S3/summary_plot/who-plot- ME .pdf]

Estimated R

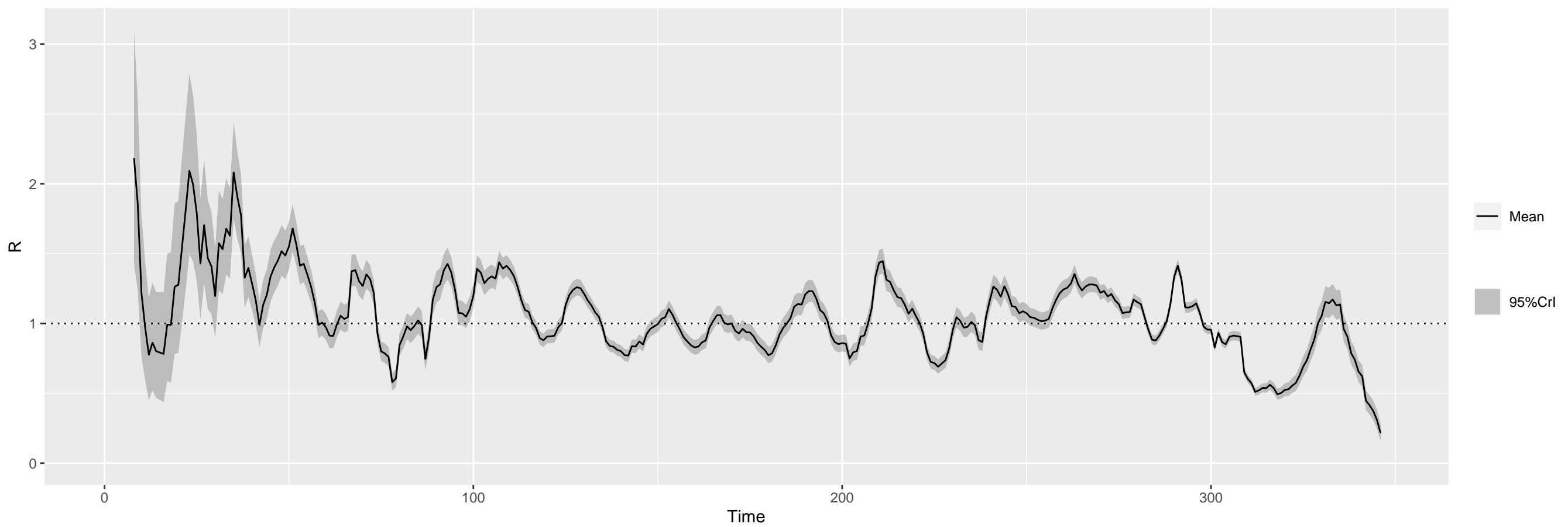

Epidemic curve

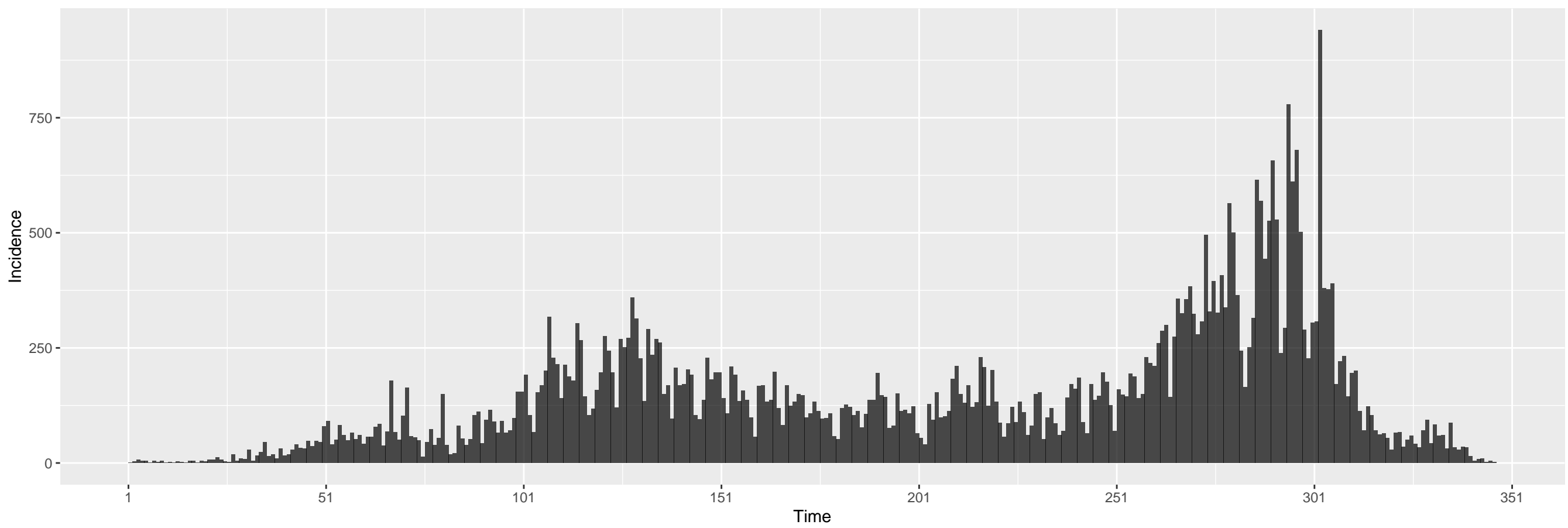

Supplement: Supplementary file 1 [file vaccines-09-00837-s001.zip › Supplementary_material/Supplementary Data S3/summary_plot/who-plot- NA .pdf]

Estimated R

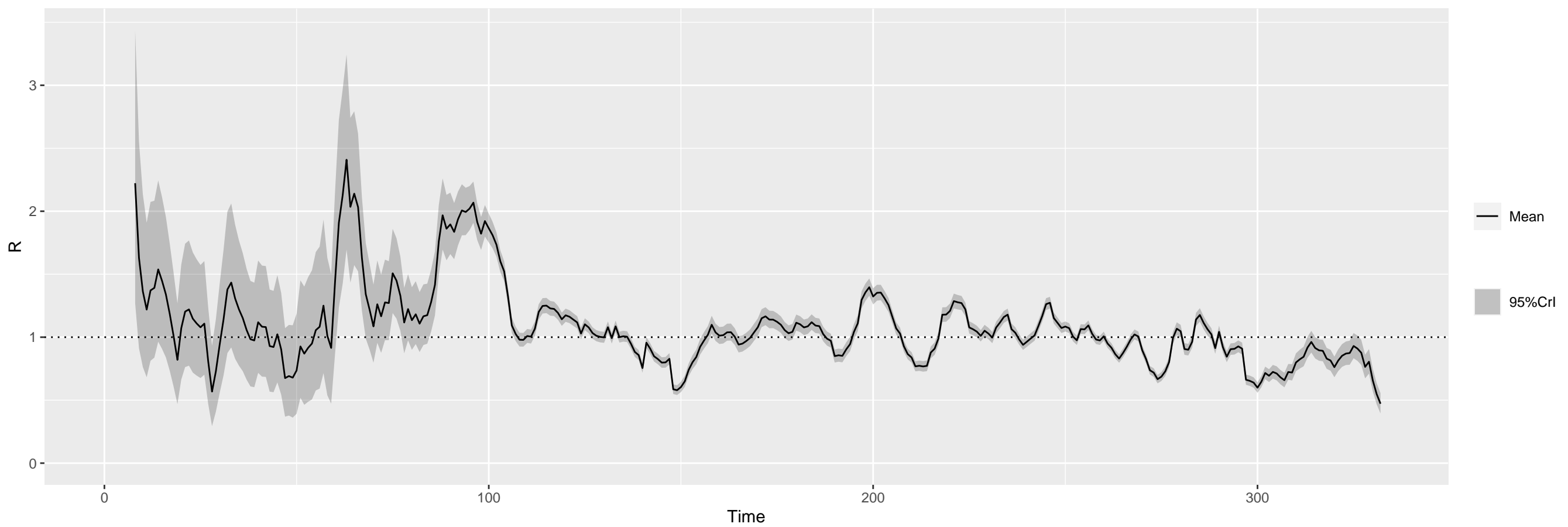

Epidemic curve

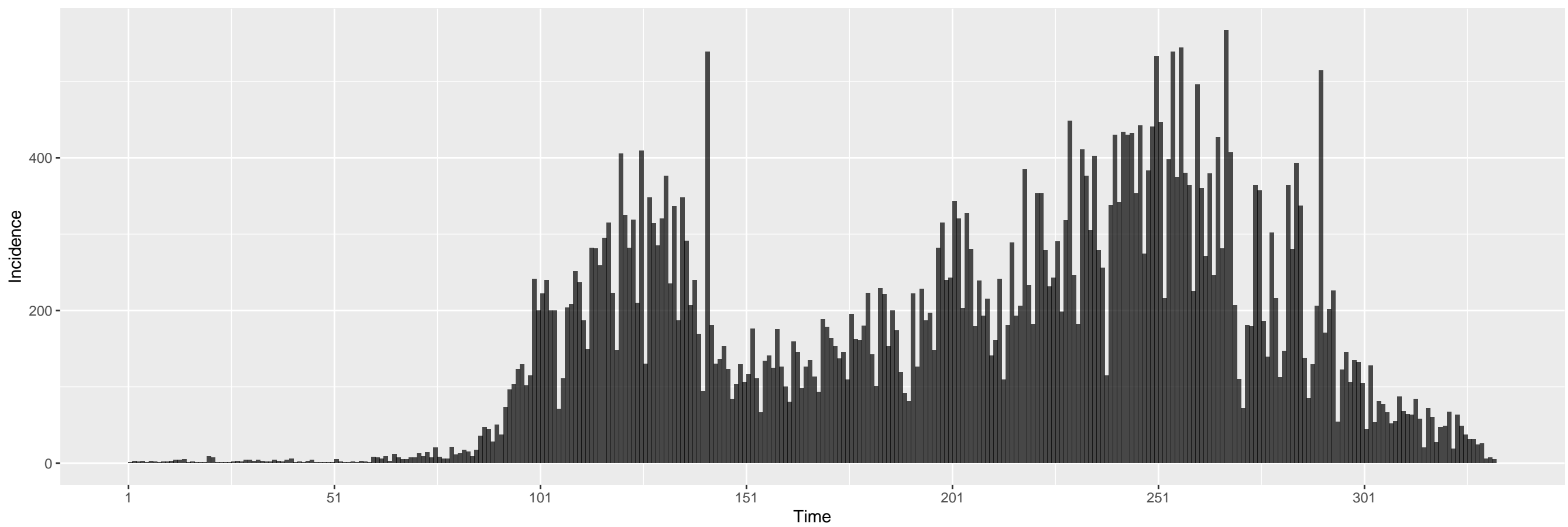

Supplement: Supplementary file 1 [file vaccines-09-00837-s001.zip › Supplementary_material/Supplementary Data S3/summary_plot/who-plot- NS .pdf]

Estimated R

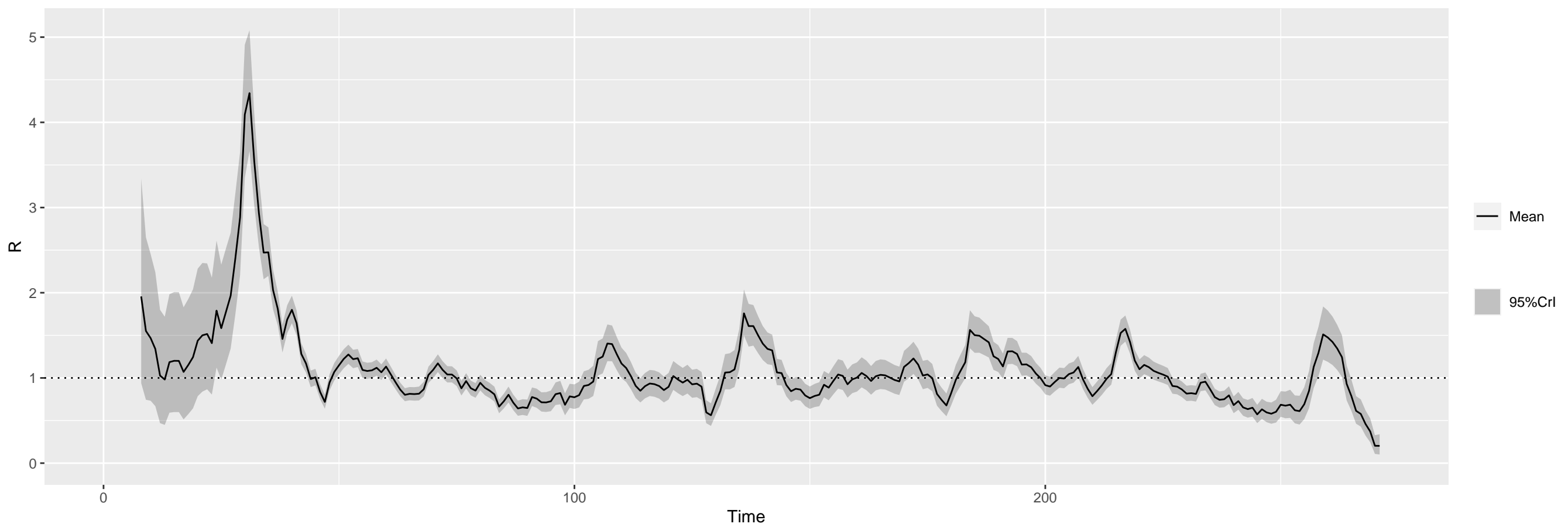

Epidemic curve

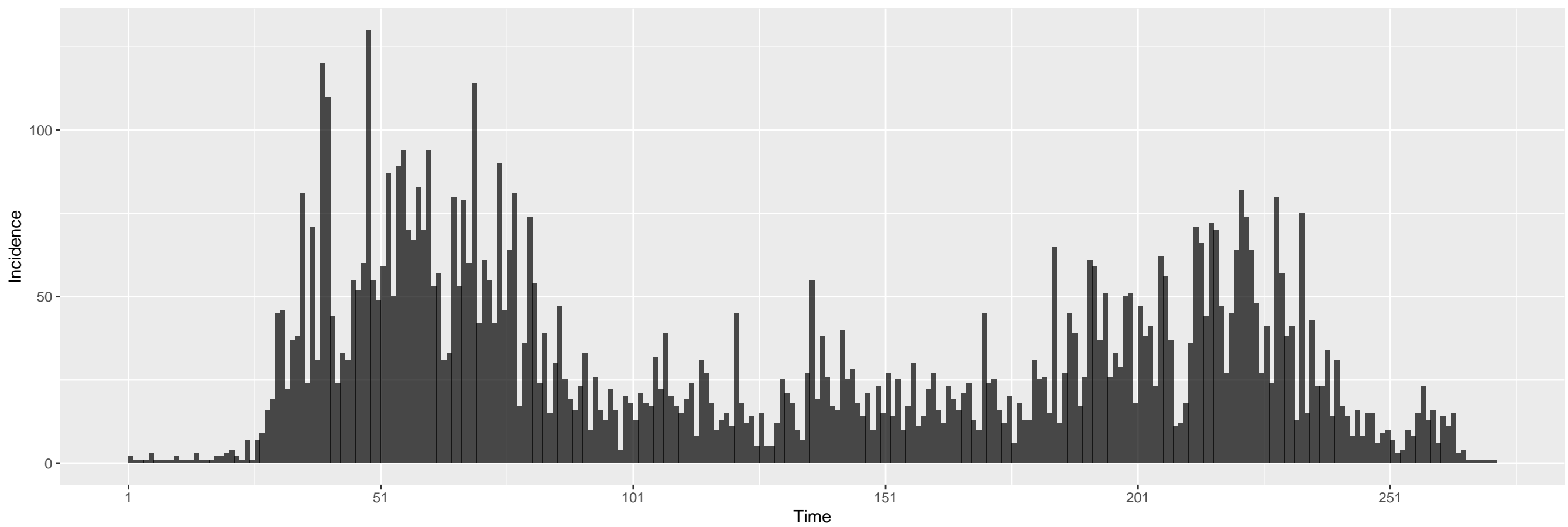

Supplement: Supplementary file 1 [file vaccines-09-00837-s001.zip › Supplementary_material/Supplementary Data S3/summary_plot/who-plot- PU .pdf]

Estimated R

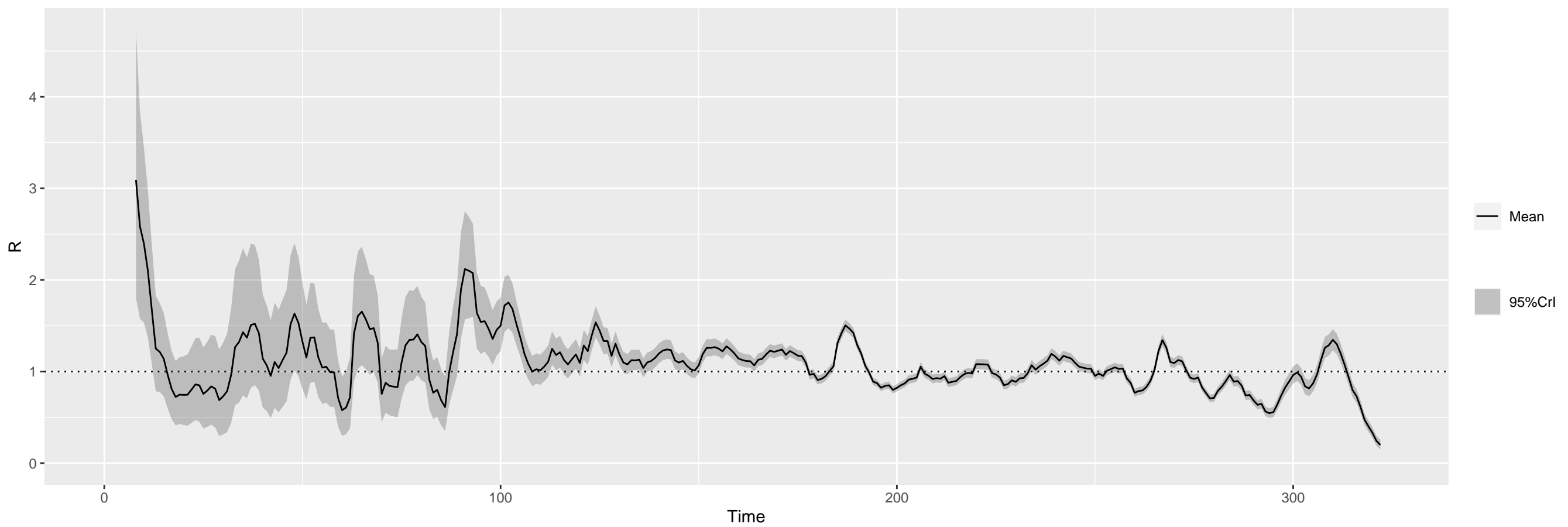

Epidemic curve

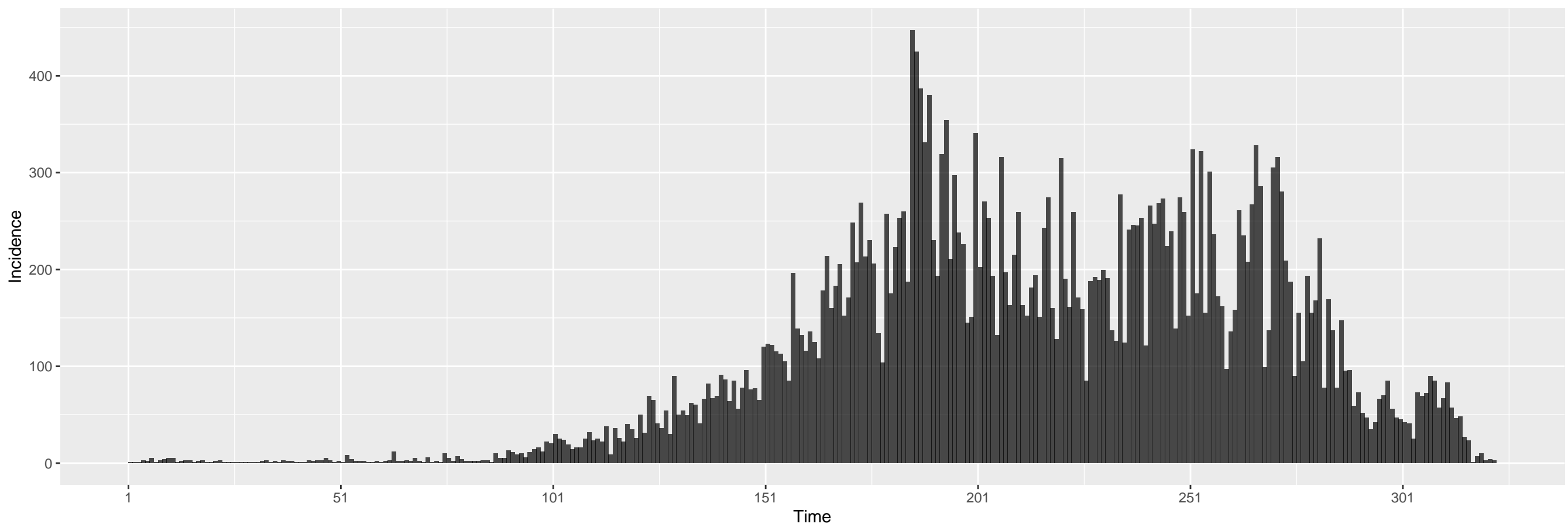

Supplement: Supplementary file 1 [file vaccines-09-00837-s001.zip › Supplementary_material/Supplementary Data S3/summary_plot/who-plot- QU .pdf]

Estimated R

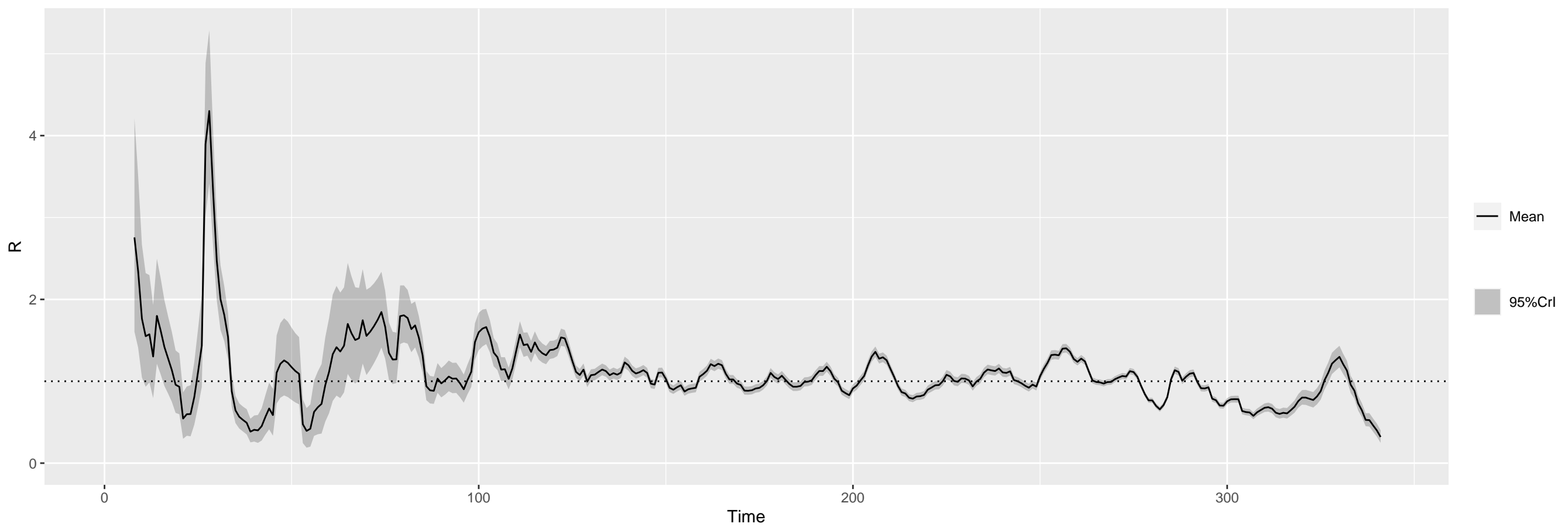

Epidemic curve

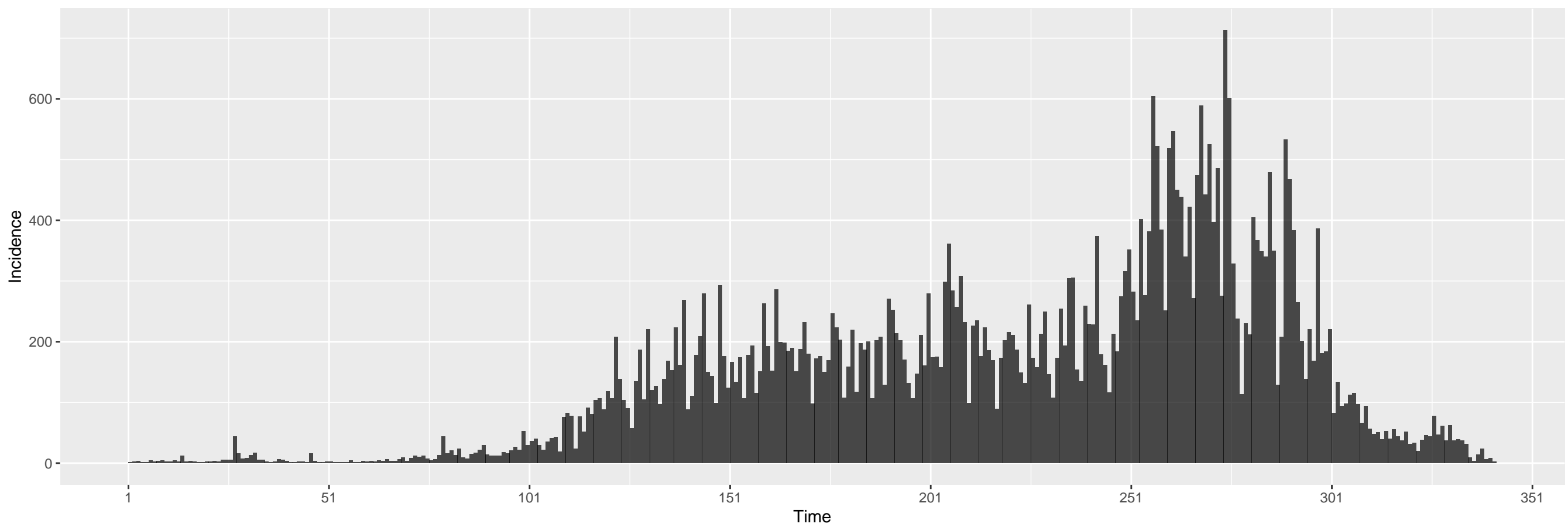

Supplement: Supplementary file 1 [file vaccines-09-00837-s001.zip › Supplementary_material/Supplementary Data S3/summary_plot/who-plot- RI .pdf]

Estimated R

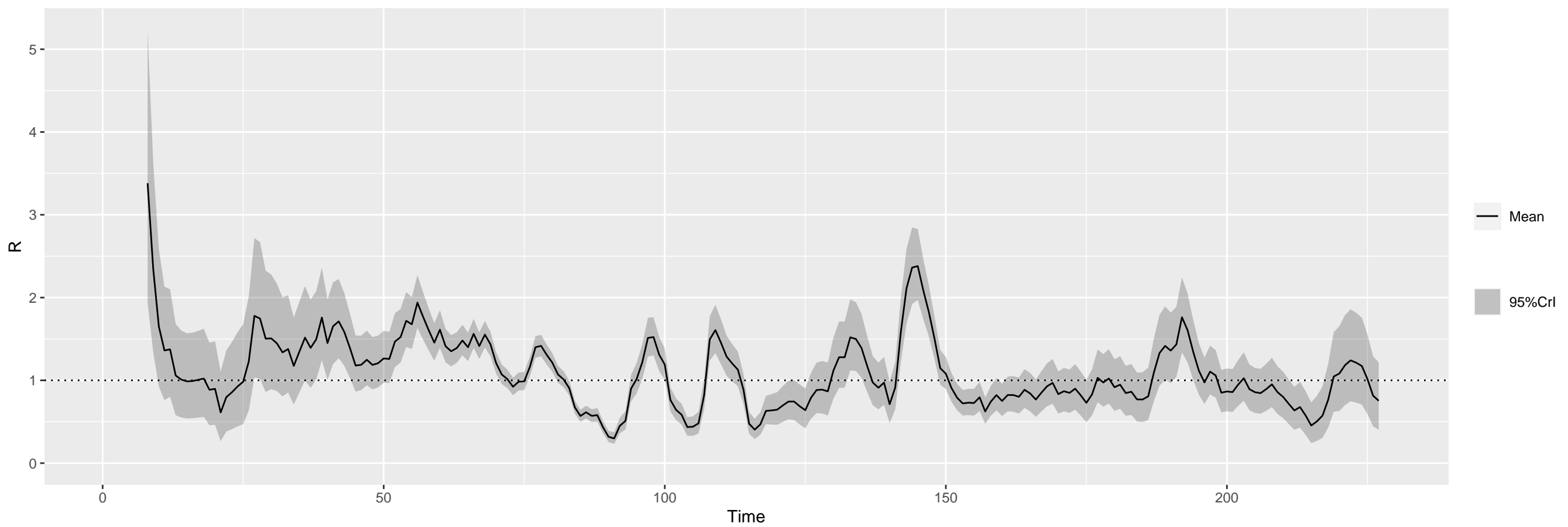

Epidemic curve

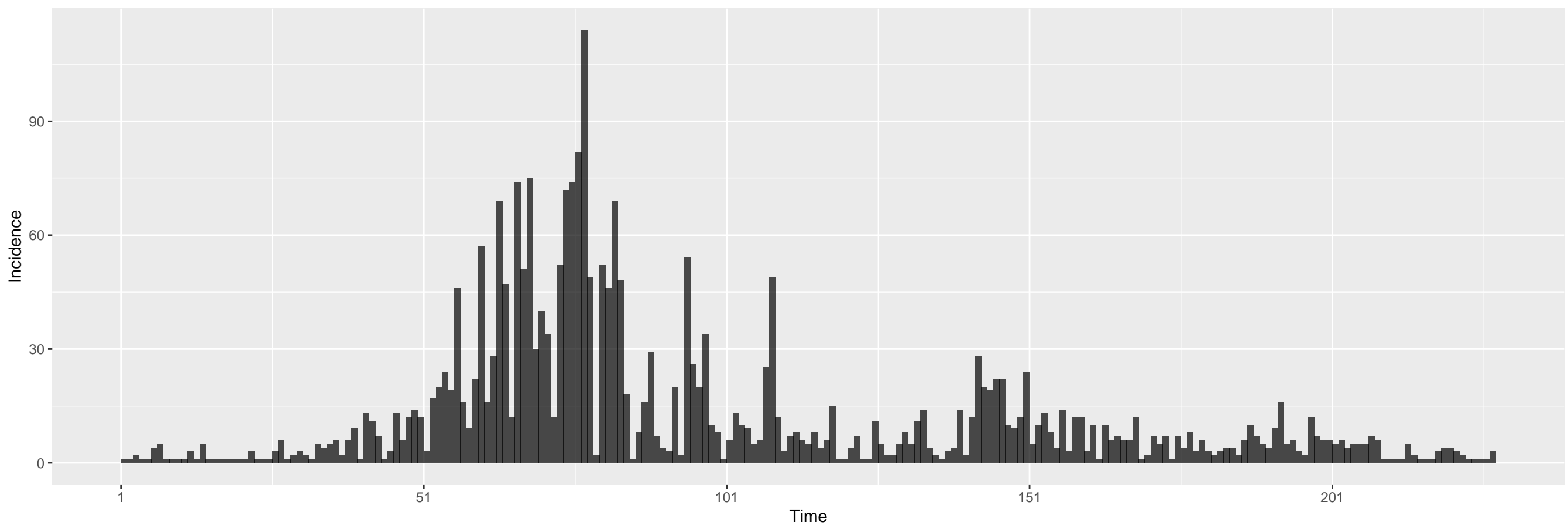

Supplement: Supplementary file 1 [file vaccines-09-00837-s001.zip › Supplementary_material/Supplementary Data S3/summary_plot/who-plot- SA .pdf]

Estimated R

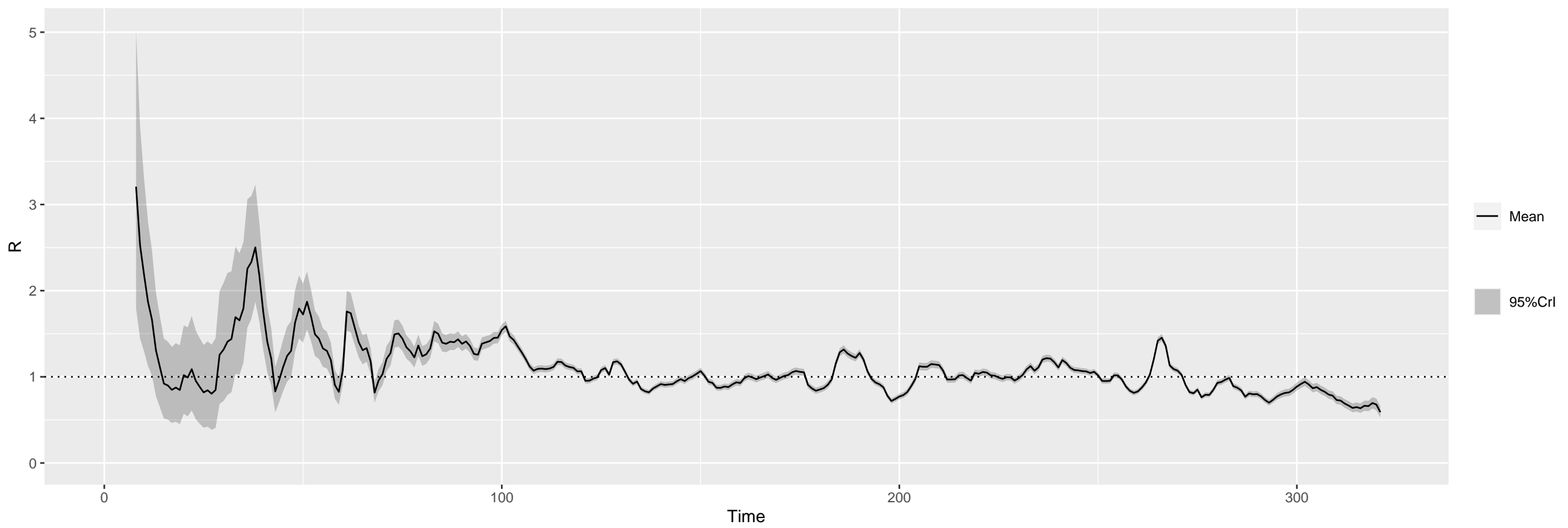

Epidemic curve

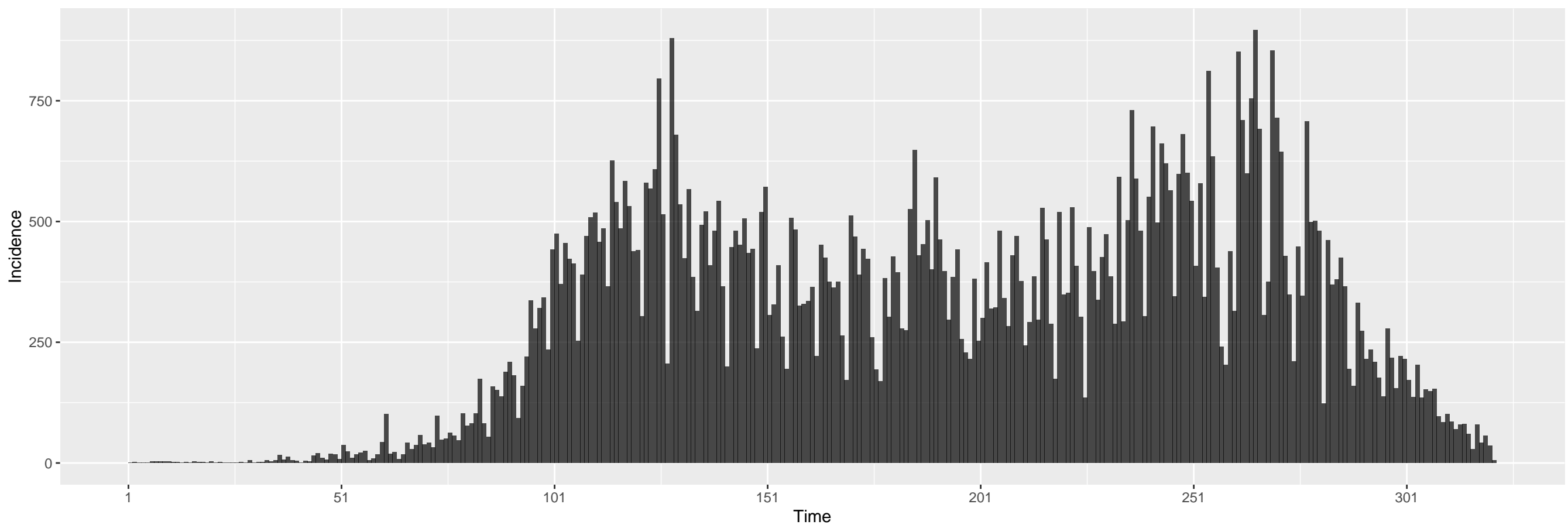

Supplement: Supplementary file 1 [file vaccines-09-00837-s001.zip › Supplementary_material/Supplementary Data S3/summary_plot/who-plot- SN .pdf]
