# Supplementary material for: Evolution and Epidemic Spread of SARS-CoV-2 in Colombia: A Year into the Pandemic
Source: Vaccines (Basel). 2021 Jul 30;9(8):837. doi: 10.3390/vaccines9080837 (PMC8402472; doi:10.3390/vaccines9080837)
Supplement: Supplementary file 1 [file vaccines-09-00837-s001.zip › Supplementary_material/Supplementary Data S3/summary_plot/who-plot- TO .pdf]

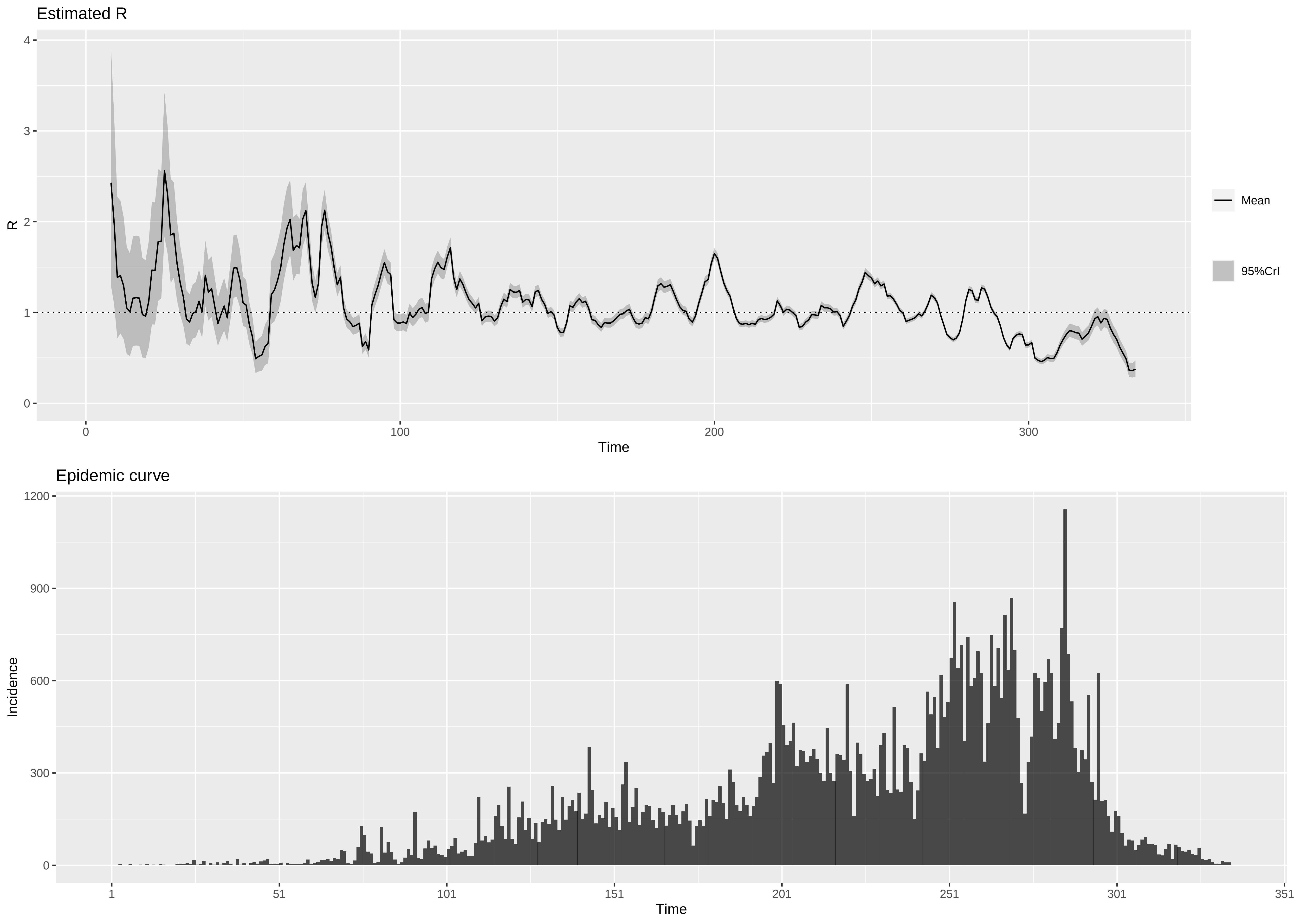
Estimated R
R
Time
Mean
95%CrI
0
1
2
3
4
0
100
200
300
Epidemic curve
Incidence
Time
0
300
600
900
1200
1
51
101
151
201
251
301
351
