# Supplementary material for: Evolution and Epidemic Spread of SARS-CoV-2 in Colombia: A Year into the Pandemic
Source: Vaccines (Basel). 2021 Jul 30;9(8):837. doi: 10.3390/vaccines9080837 (PMC8402472; doi:10.3390/vaccines9080837)
Supplement: Supplementary file 1 [file vaccines-09-00837-s001.zip › Supplementary_material/Supplementary Data S3/summary_plot/who-plot- VA .pdf]

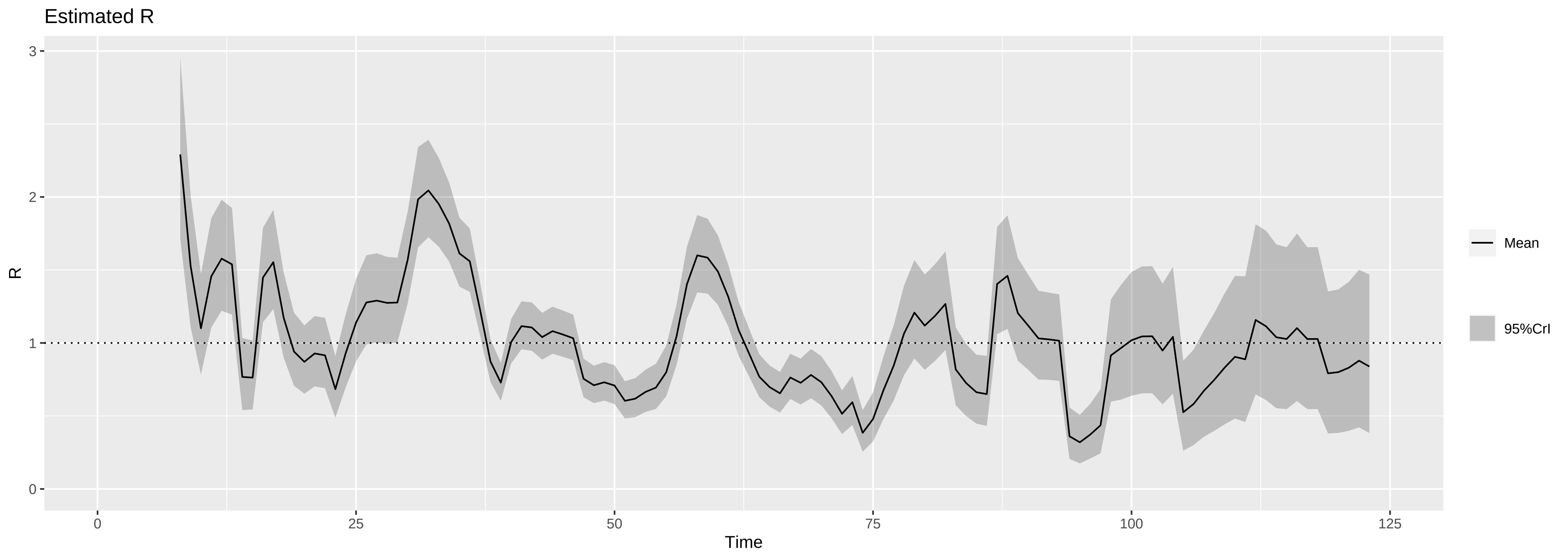
Estimated R
R
3
2
1
0
Time
0
25
50
75
100
125
Mean
95%CrI

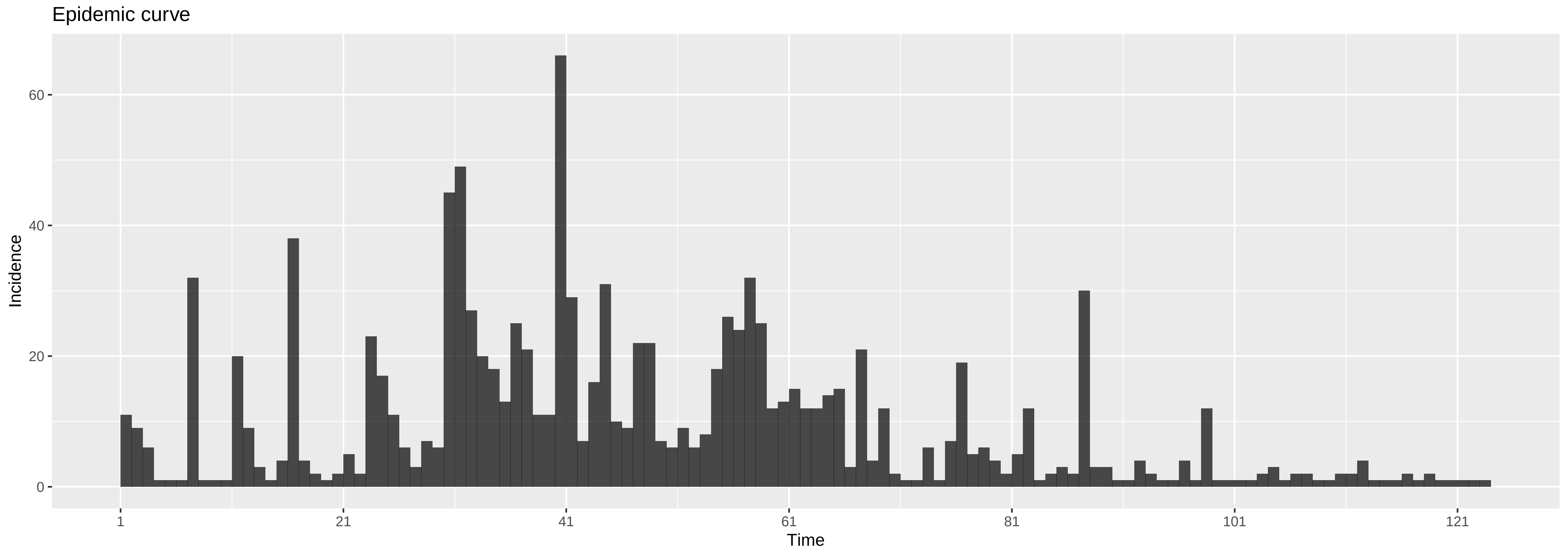
Epidemic curve
Incidence
60
40
20
0
Time
1
21
41
61
81
101
121
