# Supplementary material for: Evolution and Epidemic Spread of SARS-CoV-2 in Colombia: A Year into the Pandemic
Source: Vaccines (Basel). 2021 Jul 30;9(8):837. doi: 10.3390/vaccines9080837 (PMC8402472; doi:10.3390/vaccines9080837)
Supplement: Supplementary file 1 [file vaccines-09-00837-s001.zip › Supplementary_material/Supplementary Figs. S1.zip!/plots/COVID19-AM-Amazonas-06_03_2021.pdf]

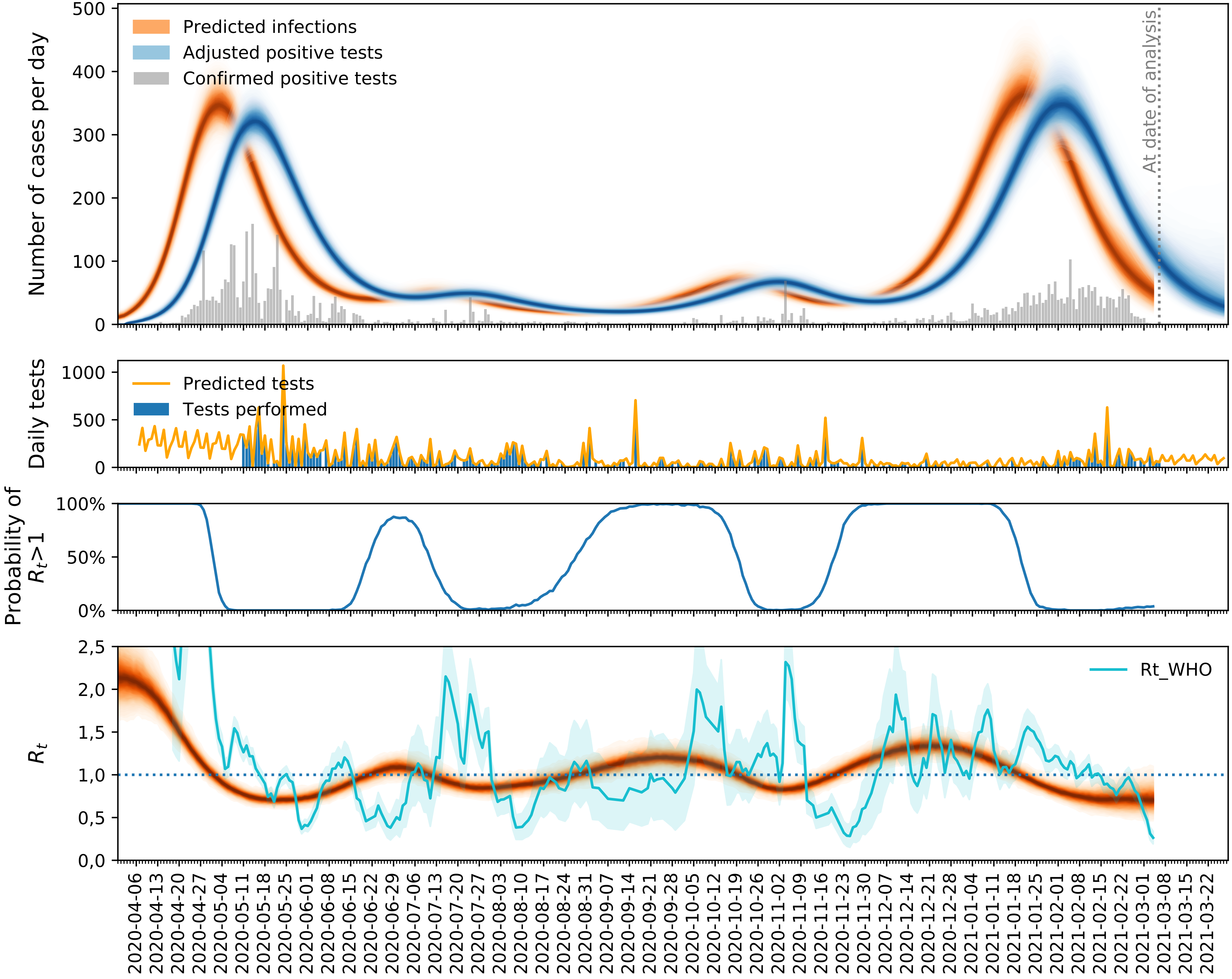

Number of cases per day
500
400
300
200
100
0
Predicted infections
Adjusted positive tests
Confirmed positive tests
At date of analysis
Daily tests
1000
500
0
Predicted tests
Tests performed
Probability of R_t>1
100%
50%
0%
R_t
2,5
2,0
1,5
1,0
0,5
0,0
Rt_WHO
2020-04-06
2020-04-13
2020-04-20
2020-04-27
2020-05-04
2020-05-11
2020-05-18
2020-05-25
2020-06-01
2020-06-08
2020-06-15
2020-06-22
2020-06-29
2020-07-06
2020-07-13
2020-07-20
2020-07-27
2020-08-03
2020-08-10
2020-08-17
2020-08-24
2020-08-31
2020-09-07
2020-09-14
2020-09-21
2020-09-28
2020-10-05
2020-10-12
2020-10-19
2020-10-26
2020-11-02
2020-11-09
2020-11-16
2020-11-23
2020-11-30
2020-12-07
2020-12-14
2020-12-21
2020-12-28
2021-01-04
2021-01-11
2021-01-18
2021-01-25
2021-02-01
2021-02-08
2021-02-15
2021-02-22
2021-03-01
2021-03-08
2021-03-15
2021-03-22
