# Supplementary material for: Evolution and Epidemic Spread of SARS-CoV-2 in Colombia: A Year into the Pandemic
Source: Vaccines (Basel). 2021 Jul 30;9(8):837. doi: 10.3390/vaccines9080837 (PMC8402472; doi:10.3390/vaccines9080837)
Supplement: Supplementary file 1 [file vaccines-09-00837-s001.zip › Supplementary_material/Supplementary Figs. S1.zip!/plots/COVID19-BL-Bolívar-06_03_2021.pdf]

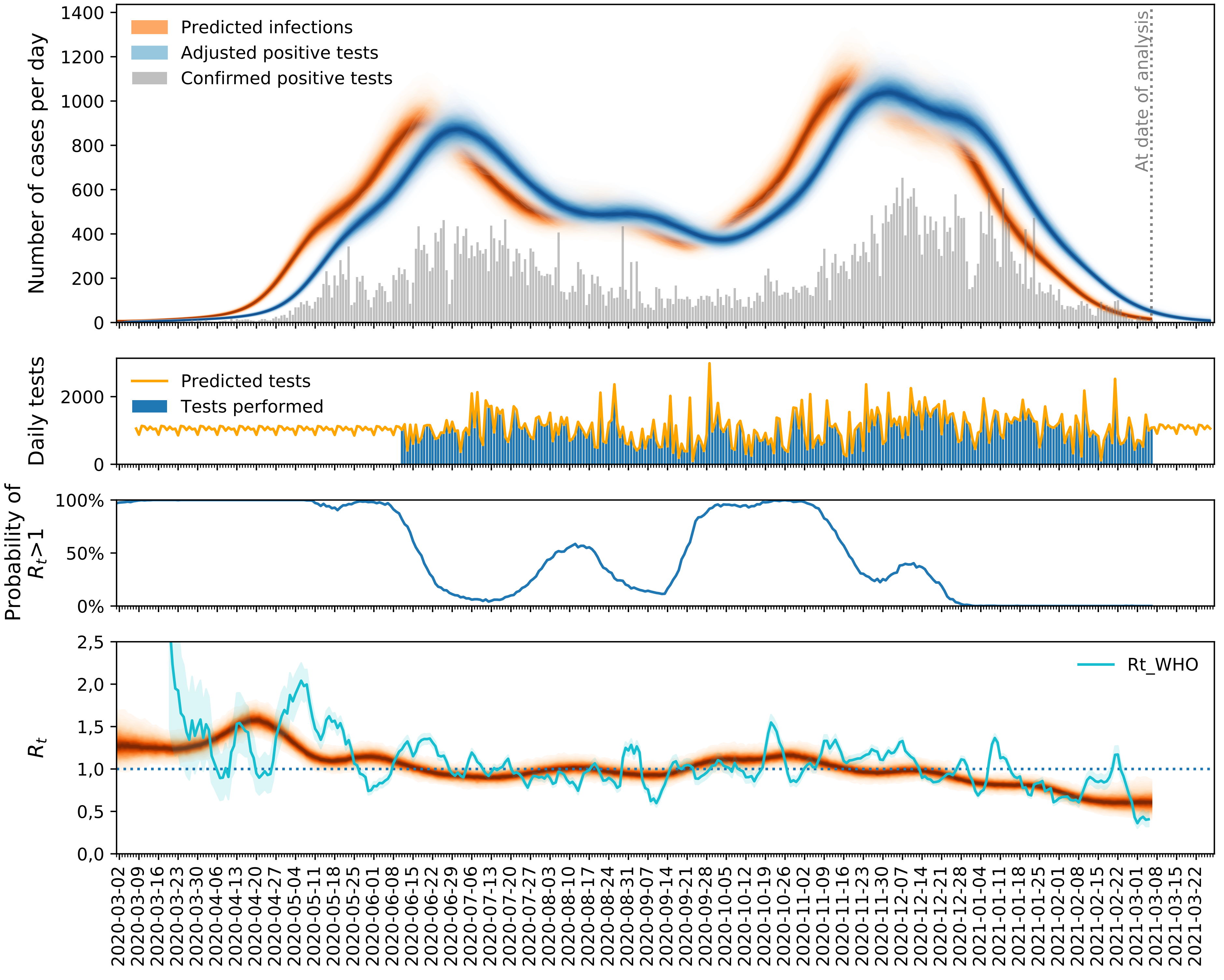

Number of cases per day
Predicted infections
Adjusted positive tests
Confirmed positive tests
At date of analysis
Daily tests
Predicted tests
Tests performed
Probability of R_t>1
R_t
Rt_WHO
